# H9N2 avian influenza virus dispersal along Bangladeshi poultry trading networks

**DOI:** 10.1093/ve/vead014

**Published:** 2023-02-25

**Authors:** L Carnegie, M Hasan, R Mahmud, M A Hoque, N Debnath, M H Uddin, N S Lewis, I Brown, S Essen, Md Giasuddin, D U Pfeiffer, M A Samad, P Biswas, J Raghwani, G Fournié, S C Hill

**Affiliations:** Department of Pathobiology and Population Sciences, Royal Veterinary College, University of London, Hatfield, Hertfordshire AL9 7TA, UK; Animal Health Research Division, Bangladesh Livestock Research Institute (BLRI), Dhaka 1341, Bangladesh; Department of Medicine & Surgery, Faculty of Veterinary Medicine, Chattogram Veterinary and Animal Sciences University (CVASU), Zakir Hossain Road, Khulshi, Chattogram 4202, Bangladesh; Department of Medicine & Surgery, Faculty of Veterinary Medicine, Chattogram Veterinary and Animal Sciences University (CVASU), Zakir Hossain Road, Khulshi, Chattogram 4202, Bangladesh; Department of Medicine & Surgery, Faculty of Veterinary Medicine, Chattogram Veterinary and Animal Sciences University (CVASU), Zakir Hossain Road, Khulshi, Chattogram 4202, Bangladesh; Department of Medicine & Surgery, Faculty of Veterinary Medicine, Chattogram Veterinary and Animal Sciences University (CVASU), Zakir Hossain Road, Khulshi, Chattogram 4202, Bangladesh; Department of Virology, Animal and Plant Health Agency (APHA), Woodham Lane, New Haw, Addlestone, Surrey KT15 3NB, UK; Department of Virology, Animal and Plant Health Agency (APHA), Woodham Lane, New Haw, Addlestone, Surrey KT15 3NB, UK; Department of Virology, Animal and Plant Health Agency (APHA), Woodham Lane, New Haw, Addlestone, Surrey KT15 3NB, UK; Animal Health Research Division, Bangladesh Livestock Research Institute (BLRI), Dhaka 1341, Bangladesh; Department of Pathobiology and Population Sciences, Royal Veterinary College, University of London, Hatfield, Hertfordshire AL9 7TA, UK; Department of Infectious Diseases and Public Health, City University of Hong Kong, 83 Tat Chee Ave, Kowloon Tong, Hong Kong SAR, PR China; Animal Health Research Division, Bangladesh Livestock Research Institute (BLRI), Dhaka 1341, Bangladesh; Department of Microbiology and Veterinary Public Health, Chattogram Veterinary and Animal Sciences University (CVASU), Zakir Hossain Road, Khulshi, Chattogram 4202, Bangladesh; Department of Pathobiology and Population Sciences, Royal Veterinary College, University of London, Hatfield, Hertfordshire AL9 7TA, UK; Department of Pathobiology and Population Sciences, Royal Veterinary College, University of London, Hatfield, Hertfordshire AL9 7TA, UK; Université de Lyon, INRAE, VetAgro Sup, UMR EPIA, Campus vétérinaire de VetAgro Sup, 1 avenue Bourgelat, Marcy, l’Etoile 69280, France; Université Clermont Auvergne, INRAE, VetAgro Sup, UMR EPIA, Centre INRAE Clermont-Auvergne-Rhône-Alpes, Saint Genes Champanelle 63122, France; Department of Pathobiology and Population Sciences, Royal Veterinary College, University of London, Hatfield, Hertfordshire AL9 7TA, UK

**Keywords:** avian influenza virus, Bangladesh, H9N2, phylodynamics, value chain, live bird market

## Abstract

Avian influenza virus subtype H9N2 is endemic in Bangladesh’s poultry population. The subtype affects poultry production and poses a potential zoonotic risk. Insufficient understanding of how the poultry trading network shapes the dissemination of avian influenza viruses has hindered the design of targeted interventions to reduce their spread. Here, we use phylodynamic analyses of haemagglutinin sequences to investigate the spatial spread and dispersal patterns of H9N2 viruses in Bangladesh’s poultry population, focusing on its two largest cities (Dhaka and Chattogram) and their poultry production and distribution networks. Our analyses suggest that H9N2 subtype avian influenza virus lineage movement occurs relatively less frequently between Bangladesh’s two largest cities than within each city. H9N2 viruses detected in single markets are often more closely related to viruses from other markets in the same city than to each other, consistent with close epidemiological connectivity between markets. Our analyses also suggest that H9N2 viruses may spread more frequently between chickens of the three most commonly sold types (sunali—a cross-bred of Fayoumi hen and Rhode Island Red cock, deshi—local indigenous, and exotic broiler) in Dhaka than in Chattogram. Overall, this study improves our understanding of how Bangladesh’s poultry trading system impacts avian influenza virus spread and should contribute to the design of tailored surveillance that accommodates local heterogeneity in virus dispersal patterns.

## Introduction

The endemic circulation of avian influenza viruses (AIVs) in Bangladesh’s poultry populations poses a threat to animal and human health (Marinova-Petkova et al. 2016; Turner et al. 2017). Low pathogenic AIV (LPAIV) subtype H9N2 was first detected in Bangladesh in 2006 (Shanmuganatham et al. 2014; Marinova-Petkova et al. 2016; Rimi et al. 2019) and is known to cause reduced egg-laying and hatching (Kariithi et al. 2020; Ripa et al. 2021). Viruses from this subtype are now common within Bangladesh at live bird markets (LBMs) where most consumers purchase poultry and are often detected at relatively lower prevalence in poultry farms or backyard rearing sites (Gerloff et al. 2016; Turner et al. 2017; Kim et al. 2018; Parvin et al. 2020; Gupta et al. 2021; Moyen et al. 2021). Close and frequent contact between birds and humans in these locations increases the risk of zoonotic spillover (El-Shesheny et al. 2020; Parvin et al. 2020; Bi, Li, and Shi 2022). Reducing virus spread is therefore particularly important for minimising pandemic emergence risk and protecting avian health (El-Shesheny et al. 2020; Parvin et al. 2020; Bi, Li, and Shi 2022).

Live poultry trading is known to contribute to AIV spread (Wu and Perrings 2018; Yang et al. 2020). Previous studies have identified broad associations between growth in poultry trade volumes and the likelihood of establishment and persistence of several infectious diseases (Wu and Perrings 2018). However, we often lack a more detailed understanding of the complexity of bird production and distribution networks and how these networks may influence AIV maintenance and spread (Gerloff et al. 2016; Parvin et al. 2020; Moyen et al. 2021). In many countries where AIVs are endemic in poultry, knowledge of viral prevalence is too limited to adequately explore how trading practices might impact circulation from infection data alone (Chattopadhyay et al. 2018; Parvin et al. 2020; Gupta et al. 2021; Moyen et al. 2021; Ripa et al. 2021). Even when surveillance is routinely conducted, LPAIV H9N2 can easily be missed because it rarely causes severe disease (Parvin et al. 2020).

Phylodynamics can reveal in-depth information about virus dynamics from virus genome sequences, including how an outbreak changes size over time, how a virus lineage spreads spatially, and what factors may influence viral dispersal patterns (Lu, Leigh Brown, and Lycett 2017; Yang et al. 2019; Kwon et al. 2020). For example, phylodynamic studies have demonstrated that live poultry trade networks shape AIV movement over large spatial scales in movement in China (H5N1, H7N9, and H5N6) (Yang et al. 2020) and that restricting duck transport and culling can suppress highly pathogenic AIV (HPAIV) H5N1 movement between regions in France (Chakraborty et al. 2022). Phylodynamic approaches, however, have rarely been applied to studying AIV in a disease-endemic poultry production system at high resolution (Yang et al. 2020). Within Bangladesh, specifically, the few published studies on the molecular epidemiology of AIVs have either relied on small numbers of samples (e.g. (Gerloff et al. 2016; Ripa et al. 2021)) or have not incorporated information on the precise sampling location or type of chicken (e.g. Marinova-Petkova et al. 2016; Parvin et al. 2019, 2020). Hence, they do not permit full exploration of how the virus dispersal patterns vary between different components of the poultry system (Marinova-Petkova et al. 2016; Parvin et al. 2019, 2020).

Within Bangladesh’s poultry production and distribution network, mobile poultry traders (‘middlemen’) collect and transport poultry from farms in rural and peri-urban areas to LBM vendors within urban areas. Here, poultry is either sold directly to end-users or may be traded further between market vendors before sale (Moyen et al. 2018, 2021; Moyen 2019; Høg et al. 2021). The most commonly sold poultry types are broiler (exotic, industrial chicken breeds), sunali (chicken crossbreed of Rhode Island Red cocks and Fayoumi hens), deshi (indigenous chicken breeds), and ducks (Moyen et al. 2018; Gupta et al. 2021). Broilers and sunalis are raised on commercial farms, whereas deshis and ducks are raised in a traditional scavenging system (‘backyard’) (Moyen et al. 2018). Recent studies show that poultry trading practices vary substantially across the network (Moyen et al. 2018, 2021; Moyen 2019). The two largest cities in Bangladesh, Dhaka and Chattogram, contain numerous LBMs where AIV infections have been consistently reported and receive poultry from largely non-overlapping regions of Bangladesh (henceforth, ‘production areas’) (Moyen et al. 2018). Trade practices differ greatly between these cities, with more frequent inter-market trading of birds in Dhaka than in Chattogram (Kim et al. 2018; Moyen et al. 2018). Broiler chickens are typically sold in LBMs close to the farm, whereas ducks and deshi chickens are generally moved over longer distances by more intermediaries (Moyen et al. 2018; Moyen 2019). Typical distance travelled from farm to market, level of intra-market trade, and the number of middlemen varies between LBMs (Kim et al. 2018; Moyen et al. 2018).

This study investigates the spatial spread and dispersal patterns of H9N2 viruses among poultry marketed in Dhaka and Chattogram cities and their respective supply networks. Unlike previous studies in Bangladesh, we curated and analysed high-resolution data on the location of sampling and type of chicken, enabling the first phylogenetic insights into how the complexities of poultry trading practices influence AIV dispersal there (Marinova-Petkova et al. 2016; Parvin et al. 2019, 2020). We use phylodynamic analyses of the rapidly evolving haemagglutinin (HA) gene segment to examine the dispersal of H9N2 virus lineages between cities and stages of the production chain. We explore the hypothesis that H9N2 virus genetic diversity is randomly distributed across LBMs. Furthermore, we query how often H9N2 lineages spread between chicken types or locations and whether observed patterns of dispersal are driven by the overlap between chicken type production areas. Finally, to investigate if AIVs with different subtypes in Bangladesh may have similar dispersal patterns to those of H9N2 in the country, we perform similar molecular clock phylogenetic analyses on Bangladeshi HPAIV H5NX sequences.

## Materials and methods

### Bangladeshi AIV sequences

Our study focuses on a dataset of AIV H9 HA segment sequences sampled between 2003 and 2019. The dataset includes eighty-two newly generated H9N2 HA sequences and H9 HA sequences for which only the HA segment was generated, but that are assumed henceforth to be from H9N2-infected birds (justification of this assumption is provided in Results and Discussion) (study sources in Table 1, accessions provided in Table S1). The dataset also includes 216 previously published sequences from both the Global Initiative on Sharing Avian Influenza Data (GISAID) (Shu and McCauley 2017) EpiFlu database (www.gisaid.org) (*n* = 211, Table S2) and from Ripa et al. (2021) (*n* = 5). Newly generated sequences were sampled through observational studies and routine surveillance in Dhaka and Chattogram from 2016 to 2018 (details in Table 1), one of which has been previously described (Kim et al. 2018).

**Table 1.** Study source of newly reported AIV HA (H9, *n* = 82; H5, *n* = 29) and NA (H5, *n* = 15) segment sequences.

Dataset and study design	Sequencing method	Available metadata	Sequences per subtype
Cross-sectional dataset: cross-sectional survey into AIV prevalence in live bird markets in Dhaka and Chattogram, during which sixty birds and fifty environmental sites were sampled at each of forty live bird markets in February to March 2016 [8]. Twenty-six of these markets were in Dhaka district and fourteen were in Chattogram district. Study previously described [8], but genomes not yet reported.	RNA was extracted using the MagMAX RNA Isolation Kit (QIAGEN, Hilden, Germany) and RT-PCR conducted using the AgPath-ID One-Step RT-PCR kit (ThermoFisher Scientific, Waltham, MA, USA) [8]. PCR amplicons spanning the HA segment were generated from positive samples using four primers for HA1 and six for HA2 (HA1: F308, R308, F754,R754; HA2: F723,R723,F531,R531,F299,R299) and were sequenced using Sanger sequencing (www.dnaseq.co.uk).	Host (species: chicken type (sunali, deshi, broiler)), market location, and sampling date.	H9; 70 HA sequences(accession numbers: Table S1)
Longitudinal dataset: A longitudinal study into AIV prevalence in live bird markets in Dhaka and Chattogram, during which sixty live birds and any observed dead birds were sampled monthly in two markets (one in Dhaka and one in Chattogram). The study ran from July 2017 and July 2018.	RNA was extracted using QIAamp Viral RNA Mini Kit (Qiagen) [27] then reverse transcribed and amplified using RT-PCR. Positive samples underwent whole genome sequencing (WGS) at APHA using an Illumina NextSeq 500/550. Consensus sequences were assembled using Velvet [28] v-1.2.10 and SPAdes [29], following the pipeline https://github.com/ellisrichardj/FluSeqID.	Host species, market location, and sampling date.	H9N2; 11 HA sequences (accession numbers: Table S1). H5NX; 24 HA sequences (accession numbers: Table S1), 10 NA sequences (accession numbers: Table S1)
Market surveillance dataset: A market surveillance study performed by the Bangladesh Livestock Research Institute (BLRI). Samples were collected during eight sampling events between November 2016 and February 2018 across thirteen live bird markets (eight in Dhaka district and five in the Gazipur district).			H9N2; 1 HA sequence (accession numbers: Table S1). H5NX; 5 HA sequences (accession numbers: Table S1), 5 NA sequences (accession numbers: Table S1)

Whilst we focus on HA here, sequences from other segments were generated for a subset of our H9N2 samples. NA gene sequences from Ripa et al. (2021) were combined with NA sequences from GISAID (Shu and McCauley 2017) EpiFlu database (H9N2; *n* = 200) corresponding to those viruses for which HA was available (Table S2) and used for a subset of analyses.

Associated information on species, location (either market- or farm-level), and sampling date was available for all newly generated sequences (Table 1). The type of chicken (either broiler, deshi, or sunali) was also available for seventy newly reported H9N2 virus genome sequences generated through a cross-sectional study of Dhaka and Chattogram’s LBMs during February–March 2016 (henceforth, the ‘cross-sectional H9N2 dataset’) (Kim et al. 2018) (Table 1). For sequences accessed from public databases, we extracted available corresponding location metadata from the Influenza Research Database (Squires et al. 2012) animal surveillance database (www.fludb.org) (Table S3). We also obtained unpublished LBM characteristics for 184 of the publicly available sequences in the H9N2 HA dataset from Professor Richard Webby (St. Jude Research Center of Excellence for Influenza Research and Surveillance) (Accession numbers in Table S4).

In addition to the H9N2 data described above, H5NX sequences were generated via next-generation sequencing through two of the three surveillance studies in Table 1. HA sequences (*n* = 29) and available corresponding NA gene segment sequences (*n* = 15) (Table 1) were combined into datasets with previously published H5NX sequences from GISAID EpiFlu (HA, *n* = 175; NA, *n* = 172) (Table S2) and Ripa et al. (2021).

### Sequence alignments and clade selection

We aligned sequences using MAFFT (Katoh et al. 2002) v7.453. We removed sequences from the alignment that either were duplicated, short (<70 per cent of the total sequence length), or indicative of containing sequencing or assembly errors.

We used an alignment of all H9NX sequences available on GISAID and our newly generated sequences to estimated preliminary phylogenies with Fasttree v2.1.11 (Price, Dehal, and Arkin 2010). All but seven HA H9 sequences from Bangladesh, including all sequences generated in this study, fell within a monophyletic clade containing H9N2 virus sequences sampled from poultry in India and Bangladesh (*n* = 333). We retained sequences from that monophyletic clade where exact sampling dates and at least district-level location data were known, resulting in 298 HA sequences in the H9N2 virus alignment (Bangladesh: *n* = 284; India: *n* = 14). We confirmed the presence of an appropriate temporal signal for both subtypes using TempEst (Rambaut et al. 2016) v1.5.3 (Figures S1 and S2). Finally, for the respective available NA sequence dataset (H9N2, *n* = 205; H5N1, *n* = 191), we repeated all alignment and temporal signal checks as detailed above.

### Molecular clock phylogenies

We investigated the introduction date of H9N2 to sampled locations using the Bayesian phylogenetic package BEAST (Drummond and Rambaut 2007; Suchard et al. 2018) v1.10.4. First, we compared four possible models: pairwise combinations of two molecular clock models (uncorrelated lognormal relaxed clock (Drummond et al. 2006) and strict clock (Marco and Ferreira 2008)) and two coalescent models (constant size (Kingman 1982; Griffiths and Tavaré 1994) and Bayesian skygrid (Gill et al. 2013)), all using an SRD06 substitution model (Shapiro, Rambaut, and Drummond 2006). We executed multiple Markov Chain Monte Carlo (MCMC) chains comprising 100 million steps and sampling every 10,000 steps. As identified using path-sampling (Lartillot and Philippe 2006) and stepping-stone-sampling analyses (Fan et al. 2011; Xie et al. 2011), the best model used the uncorrelated relaxed clock (Drummond et al. 2006) and a Bayesian skygrid coalescent prior (Gill et al. 2013). The posterior tree distributions for H9N2 and H5NX HA alignments were each obtained from two parallel MCMC chains with 250 million steps, sampling every 25,000 steps. We assessed the convergence of each run using Tracer (Rambaut et al. 2018) v1.7.1 (http://tree.bio.ed.ac.uk/software/tracer/) and confirmed the presence of appropriate parameter convergence by visually inspecting and then log combining multiple parallel runs. We summarised the information on maximum clade credibility (MCC) trees using TreeAnnotator (Drummond and Rambaut 2007) v1.10.4, with the first 10 per cent discarded as burn-in. We repeated all BEAST analyses for the NA sequence alignments using similar methods and parameters.

We used a generalised linear model (GLM) extension of the skygrid coalescent model (Gill et al. 2016; Dellicour et al. 2020) (henceforth, ‘skygrid-GLM’) to determine whether the effective population size (*N*_e_) of the H9N2 HA lineage was associated with the tonnage of chicken or duck meat production in Bangladesh. Data were available yearly for each predictor from FAOSTAT (Food and Agriculture Organization of the United Nations, 2020) statistical database. To provide correspondence with the estimation of virus effective population size at two-month intervals, we used the ‘zoo’ package (Zeileis and Grothendieck 2005) in R (RStudio Team 2020) v4.1.2 to estimate values for each predictor every two months between 2000 and 2020 using linear interpolation. We then conducted the same analysis for the H5 HA dataset.

### AIV transmission dynamics in live bird markets

We investigated whether individual H9N2 virus clades tend to be either randomly distributed across LBMs in a city or associated with specific markets. First, we identified genetically diverse ‘clusters’ (henceforth known as ‘clades’) in the HA H9N2 MCC tree using Clusterpicker (Ragonnet-Cronin et al. 2013) v1.2.3. These clades (*n* = 32) were identified according to a clade support threshold (0.7 posterior probability), which is consistent with values chosen in previous AIV phylogenetic clustering analyses (Gerloff et al. 2013; Lee et al. 2018) and a maximum sequence identity threshold (4.5 per cent genetic distance) that provided a consistent aggregation of closely related sequences (i.e. with most sequences into a specific well-supported clade). Next, to quantify lineage diversity within and between markets, we used the adjusted Rand index (Rand 1971) by means of the ‘phyclust’ package (Chen 2011) in R (RStudio Team 2020) v4.1.2 to determine the similarity of clustering by market and clade in the cross-sectional H9N2 dataset. To determine if there was a significant difference in the observed clustering pattern to that expected based on random viral movement, we compared the estimated median adjusted Rand index with a median adjusted Rand index calculated by permuting the market location 10,000 times while controlling for the city. We further generated binary adjacency networks in R (RStudio Team 2020) v4.1.2, in which the edges linked market nodes if they shared a genetically defined clade. To further test whether AIV clades are randomly distributed across markets, we determined the density of the observed network using the ‘sna’ package (Butts 2008) in R (RStudio Team 2020) v4.1.2 and subsequently compared it to the density value from permuting (*n* = 10,000) market location with respect to city. This density metric is the ratio of observed edges to the number of possible edges for the given network.

### H9N2 dispersal between different chicken types

To determine whether H9N2 virus genetic diversity is structured according to Bangladesh’s three main chicken types (broiler, sunali, and deshi) and two main cities (Dhaka and Chattogram), we used Bayesian Tip-association Significance Testing (BaTS) (Parker, Rambaut, and Pybus 2008) v1.0. We removed tips in each tree in the HA H9N2 posterior tree distribution when tip information on either chicken type and sampling city was unavailable, using the R (RStudio Team 2020) v4.1.2 library, ‘ape’ (Paradis and Schliep 2019) package. This generated a distribution of downsampled posterior trees each containing seventy tips, corresponding to those sequences in the cross-sectional dataset (Table 1). The first BaTS analyses we performed assessed if sequences tended to cluster based on the sampling city (Dhaka and Chattogram). The second set of BaTS analyses determined if sequences tended to cluster by chicken type (broiler, sunali, and deshi); these analyses were conducted separately for each city because significant clustering of sequences by the city was observed in the first BaTS analysis. For each BaTS analysis, we computed median empirical values for each BaTS statistic (association index, AI; the parsimony score, PS) (Parker, Rambaut, and Pybus 2008) using 1,000 subsampled post-burn-in posterior trees from the respective phylogeny. We calculated *P* values by permuting the market location within each city 1,000 times to determine if empirical estimates significantly differed from the null expectation (based on random viral movement).

We also used a GLM extension of a phylogeographic discrete trait analysis (DTA) in BEAST v1.10.4 (Drummond and Rambaut 2007; Faria et al. 2013; Suchard et al. 2018) to determine whether covariates of the trading network predicted the H9N2 viral lineage movement. We used the six pairwise combinations of chicken types (sunali, deshi, and broiler) sold in Dhaka and Chattogram as the six discrete traits. The GLM predictors included (i) the number of sequences associated with each of the six categories as a proxy for sampling effort (both origin and destination), (ii) the weekly estimated sales at LBMs for all six categories (both origin and destination) as detailed in Moyen (2019) and Moyen et al. (2021), (iii) a binary chicken type similarity index of the six discrete traits (i.e. same chicken type = 1 and different chicken type = 0), (iv) an equivalent city similarity index, and (v) the extent of overlap between production areas from which birds are sourced (1, Pianka index) between each discrete trait. These production areas are the set of Upazilas (sub-district) where farms supplying markets within a city are located, which were previously computed from reconstructed transaction networks based on traders’ interviews as described in Moyen et al. (2021). Descriptions for all GLM predictors included are summarised in greater detail in Table S5. We completed four separate MCMC analyses of 100 million steps sampling every 10,000 steps using the HA H9N2 empirical tree distribution downsampled to tips from the cross-sectional dataset (i.e. those tips with all appropriate metadata). The first model, termed model A, used all covariates except the chicken type and city similarity matrices. Models B and C were the same as model A but excluded the number of sequences or the weekly sales number, respectively. Model D used all five predictors. Model D was ran to check whether results obtained regarding the importance of production catchment area from models A–C were sensitive to the inclusion of chicken type and city similarity matrices, as these are somewhat correlated with production area matrices (Moyen et al. 2021). For each run, the presence of appropriate parameter convergence was confirmed visually in Tracer (Rambaut et al. 2018) v1.7.1 and multiple parallel runs for each MCMC analysis compared.

## Results

### Dynamics of avian influenza virus in Bangladesh

**Figure 1.**
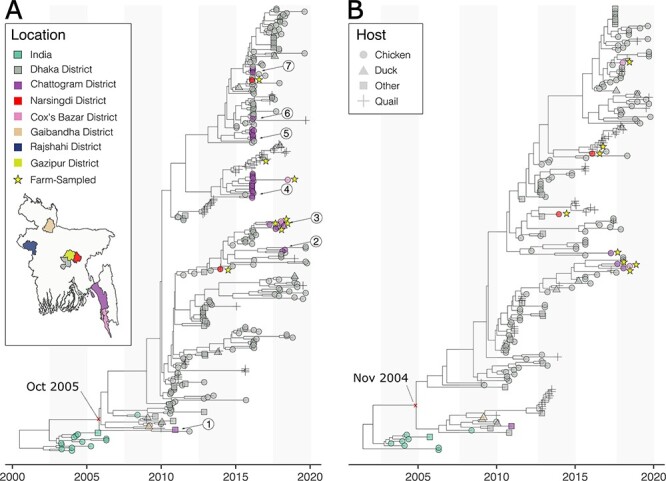
The estimated time-scaled MCC phylogenies of (A) HA H9N2 and (B) NA H9N2. Tips are coloured by the sampling location as indicated on the map and shaped by host type. Numbered labels (1–7) in the HA tree (left) designate clades of intermixing of Dhaka- and Chattogram-sampled sequences.

All but seven H9N2 sequences from Bangladesh (of which six were sampled from ducks) fell within a H9NX monophyletic clade containing virus sequences sampled from poultry in India and Bangladesh, and this clade (*n* = 298 sequences with appropriate metadata) was therefore selected for further analysis. All sequences in this clade that had a respective NA sequence were identified as being H9N2 subtype, thus strongly indicating that any HA-only sequences (i.e. cross-sectional dataset) were also H9N2 (Price, Dehal, and Arkin 2010). The most recent common ancestor of all Bangladeshi HA sequences in this major H9N2 clade was estimated around October 2005 (December 2004–July 2006: 95 per cent highest posterior distribution (HPD)) (Fig. 1A), concurrent with the countries’ first reported H9N2 cases in early 2006 (Marinova-Petkova et al. 2016). The 95 per cent HPD interval of root dates for the Bangladeshi sequences in the NA MCC tree (November 2003–November 2005) (Fig. 1B) overlaps with the respective distribution in the HA MCC trees (Fig. 1A).

Bayesian skygrid reconstructions from the HA segment (Figures S3A and B) indicate that the effective population size of H9N2 may have increased in Bangladesh since it was first detected, but neither chicken meat nor duck meat production was found to be a significant predictor of the effective population size (Figures S3C and D).

Several clades in the HA H9N2 tree contain sequences from only Dhaka or from only Chattogram, suggesting that LPAIV transmission might occur preferentially between birds sold in each city (Fig. 1A). However, there are seven instances where sequence(s) from one of the cities fall within a clade where the basal sequence(s) to the clade were sampled in the other city (Fig. 1A). This is consistent with recurrent sharing of virus lineages between Dhaka and Chattogram or their introduction from the same source. The NA phylogeny contains very few sequences sampled in Chattogram (H9N2; Chattogram *n* = 4), making it difficult to assess whether H9N2 virus NA lineages are similarly structured between the two cities (Fig. 1B).

Only around 3 per cent of H9N2 HA sequences (8/298) were recorded as being sampled from farms. Farms have been sampled less intensively than LBMs in Bangladesh, in part because lower AIV prevalence and greater distance from urban research laboratories makes surveillance more challenging than at LBMs (Kim et al. 2018; Parvin et al. 2020; Moyen et al. 2021; Ripa et al. 2021). The farm-sampled HA sequences fall throughout the phylogeny (Fig. 1A), sometimes within clades containing sequences from both farms and markets sampled within similar time periods (Fig. S4). Farm-sampled genomes are too rare here to make robust conclusions regarding the direction of viral dispersal between farms and markets.

To begin to explore whether AIVs of different subtypes may show similar patterns to those of H9N2, we attempted similar analyses on HPAIV H5NX. The 95 per cent HPD interval of root dates for the H5 Bangladeshi sequences in both the HA and NA MCC trees overlap (Fig. S5). H5 HA sequences are proportionally less well sampled in Chattogram than in Dhaka (Chattogram, *n* = 6; Dhaka, *n* = 185) (Figures S5A and S6). However, the six H5 sequences sampled in the Chattogram fall into three different clades (Fig. S5A). Only 2 per cent of H5NX HA sequences were sampled from farms, but again these fell throughout the respective trees (Fig. S5A). Neither chicken nor duck meat production was a significant predictor of the effective population size of H5NX (Fig. S3). Whilst these results are extremely limited, there is therefore no clear indication from currently available data that H5 HPAIV exhibits strongly different patterns to H9N2.

### Distribution of virus clades across LBMs

**Figure 2.**
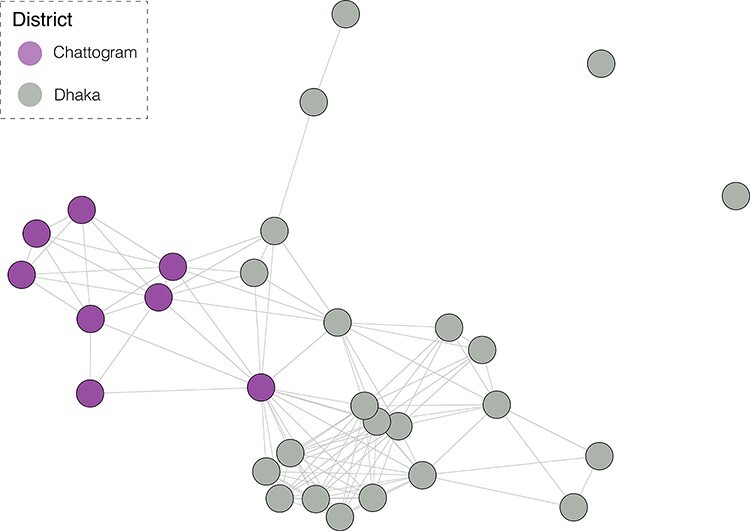
Binary adjacency network of Dhaka and Chattogram markets in the cross-sectional H9N2 dataset. Nodes represent markets, each containing a unique market ID number in white text. Nodes are linked if samples from those markets occur in the same genetically defined cluster. Nodes are coloured by city, with purple nodes indicating markets in Chattogram and grey nodes indicating markets in Dhaka.

We investigated whether individual H9N2 HA clades tended to be randomly distributed across LBMs in a city or associated with specific markets. We quantified the diversity of viruses within and between markets in a city by employing the adjusted Rand index to measure the similarity of clustering by market and virus clade for the cross-sectional H9N2 dataset. We found no significant difference (*P* = 0.993) between the median adjusted Rand index calculated from the empirical and permuted data (Table S6). Therefore, differences in viral genetic diversity between and within markets were not significantly different. The binary adjacency network analysison the same cross-sectional H9N2 dataset found no significant difference (*P* = 0.881) between the median network density estimated from the permuted data and the median network density calculated from the empirical data (Table S7 and  Fig. 2). Therefore, we found no significant difference in the sharing of clades between markets in a city.

### Phylogenetic clustering by city and chicken types

To identify possible subnational differences in AIV dynamics between different major cities, we investigated whether HA H9N2 virus sequences sampled from Dhaka and Chattogram phylogenetically clustered by city and chicken type (deshi, sunali, and broiler) with BaTs (Parker, Rambaut, and Pybus 2008) v 1.0 (Fig. S7). The analyses demonstrate significant clustering of sequences by city based on AI and PS statistics (*P* < 0.001) (Table S8). The AI statistic suggested sequences clustered significantly by chicken types in Chattogram (*P* = 0.036) but not in Dhaka (*P* = 0.278) (Table S9). In contrast to the respective AI statistic, the PS statistic did not significantly support clustering by chicken type in Chattogram (*P* = 0.071) (Table S9). This discrepancy could reflect the lower statistical power of the PS compared to the AI test (Parker, Rambaut, and Pybus 2008).

**Figure 3.**
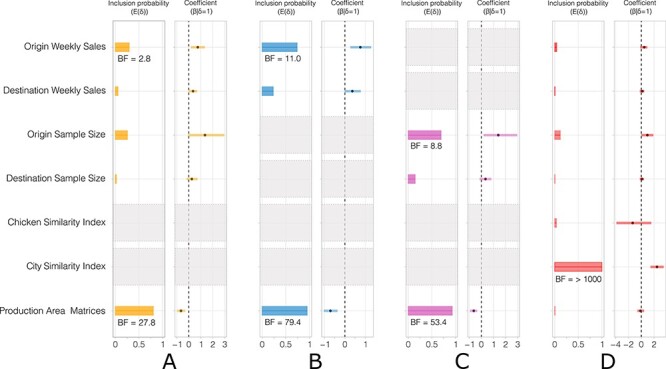
Predictors of HA H9N2 dispersal among the six pairwise combinations of three main chicken types sold in Bangladesh (sunali, deshi, and broiler) and the two largest cities (Dhaka and Chattogram) in four separate DTA-GLM analyses (A–D). Inclusion probability is an estimate of the posterior expectation for the indicator variable associated with each predictor *E*(*δ*). It suggests the likelihood that the predictor has a meaningful impact on viral diffusion. Bayes Factor (BF) support values for predictors (when >3 BF) are indicated by black text annotations. Coefficient (*β*|*δ* = 1) represents the contribution of each predictor on a log scale conditional when the predictor is included in the model, with the 95 per cent highest posterior density interval of the log GLM coefficients (*β*) represented by horizontal lines from the mean. Grey boxes indicate that the predictor was not included in the specific DTA-GLM analyses.

A GLM extension of discrete trait phylogeography was used to determine whether different features of the trading network predicted HA H9N2 virus dispersal between six pairwise combinations of chicken type (sunali, deshi, and broiler) and city (Dhaka and Chattogram). The results are summarised in  Fig. 3 and Table S10. Our initial analyses (model A; Fig. 3A and Table S10A) involving all covariates bar the chicken type and city similarity matrices showed very strong support (Bayes factor (BF) = 27.8; following Stefan et al. 2019) for greater virus dispersal as the overlap in production areas from which chickens are sourced before being sold increased. This finding was consistent to subsequent analyses that excluded covariates for the number of sequences (model B; Fig. 3B and Table S10B) or the weekly sales number (model C; Fig. 3C and Table S10C) (BF > 70 and >50, respectively). However, when the city and chicken type similarity matrices were also included as predictors (model D; Fig. 3D and Table S10D), the association of greater production overlap with greater virus dispersal was no longer observed (production area matrices; BF < 1). Instead, the higher diffusion was strongly associated (city similarity index BF > 1,000; following Stefan et al. 2019) with the chicken presence in the same city. Thus, whether the bird was sampled in the same city is likely the primary predictor of virus dispersal of all covariates considered here (Moyen et al. 2018, 2021).

## Discussion

Controlling AIV transmission in Bangladesh is complicated by our lack of understanding of whether virus spread disproportionately occurs within specific components of the poultry system (e.g., with farms, markets, or different chicken types as more important sources of infection). Here, we undertook a phylodynamic investigation to understand H9N2 viral spread in Bangladesh’s poultry system, focusing on Dhaka and Chattogram markets and their respective production areas. For H9N2 analyses, we first identified a monophyletic HA H9NX clade containing sequences sampled from poultry in Bangladesh and India, for which all available respective NA segment sequence were H9N2. Our analyses of this clade show that most H9N2 dispersal likely occurs between birds within the same major city, but that virus lineages are shared between Dhaka and Chattogram. H9N2 viruses from different lineages appear to be randomly distributed across a city’s markets. We found regional differences in H9N2 virus spread suggesting more frequent viral transmission between chickens of different types in Dhaka compared to in Chattogram.

Our analyses suggest that H9N2 subtype lineage movement occurs less frequently between Bangladesh’s two largest cities than within each city. Whilst present, virus genetic structuring by city appears less strong than previously hypothesised based on low levels of overlap in the geographic regions from which markets in Dhaka and Chattogram source birds (Moyen 2019). It is perhaps most likely that small overlaps in production areas allow for shared H9N2 lineage introductions to both cities identified here, given the frequent movement of birds from farm to markets and the relatively high prevalence of infection among traded chickens. However, more extensive and reliable data on mobile poultry traders’ movements are required to robustly rule out possible alternative explanations (Moyen 2019; Høg et al. 2021; Moyen et al. 2021; Ripa et al. 2021), as all current estimates for chicken type production areas are based on reporting data that may be subject to memory recall errors (Høg et al. 2019; Moyen 2019; Moyen et al. 2021). Alternative or complementary explanations include rapid, direct viral movement between Dhaka and Chattogram (perhaps associated with direct poultry trading between individuals in each city) and slower indirect virus spread across unsampled intermediate locations (Høg et al. 2019; Moyen 2019; Moyen et al. 2021). Although the lack of wild bird sequences in our datasets prevents any robust conclusions of the contribution of wild birds to H9N2 mobility here, if wild birds played an important role in such viral movements, we may expect Bangladeshi sequences to be more frequently intermixed with samples from locations linked to the country via wild migration routes (e.g. China and Mongolia) (Tian et al. 2015; Lycett, Duchatel, and Digard 2019).

H9N2 viruses detected within one market are often interspersed phylogenetically with those from other markets, and genetically different viruses from this subtype appear randomly distributed across markets. This may indicate strong epidemiological connections between these markets, leading to frequent virus spread between them or from shared sources, as suggested by the highly connected trading network captured by Moyen et al. (2018). Our observations suggest that viral genetic diversity within a given LBM might not be hugely dissimilar to viral genetic diversity across all markets in a city, meaning surveillance of only a few markets within a city may be sufficient to capture viral diversity. This pattern could be generated in several ways, including frequent introduction of viral clades to LBMs (as suggested by Moyen et al. 2021) followed by limited intra-market persistence. However, we could not test this or other hypotheses regarding lineage introduction and persistence here because many markets in our dataset were represented by only one sequence.

Our analyses indicate that the H9N2 viruses generally cluster more frequently by chicken type in Chattogram than in Dhaka, suggesting that it may be necessary to consider subnational variation in production and trading processes when evaluating AIV dispersal. This finding could be consistent with several non-exclusive scenarios. In the first scenario, farms in different geographic regions might harbour geographically distinct viruses, which are then imported regularly to LBMs (Ripa et al. 2021). In that case, more significant overlap in the production areas from which different bird types are typically sourced could lead to greater mixing of viruses between those bird types. Weak evidence that H9N2 dispersal between different bird types in each city is associated with their degree of production area overlap somewhat supports this hypothesis. However, this finding is not robust to the inclusion of additional predictors. In the second scenario, our finding may result from differences in the trading practices between cities. Specifically, in Chattogram, middlemen typically sell poultry directly to stallholders (retailers or wholesaler), whereas in Dhaka, inter-market transactions are relatively more common (Moyen 2019; Moyen et al. 2021). As middlemen rarely supply more than one chicken type, there is less opportunity for direct viral transmission between chicken types during middlemen-facilitated transport to Chattogram’s LBMs. In contrast, LBMs often sell multiple chicken types, so higher rates of market-to-market trading in Dhaka may increase opportunities for AIV dispersal between different bird types (Moyen 2019; Moyen et al. 2021).

Patterns of AIV dispersal identified here via H9N2 analyses may be different for non-H9N2 subtypes. For instance, HPAIV lineages may be relatively more detectable by poultry traders as host infections would often be more symptomatic (Parvin et al. 2018; Ripa et al. 2021). This could result in contrasting management practices for birds infected with LPAI and HPAI viruses and subsequently differing viral dispersal patterns (Rimi et al. 2019). Although our limited analyses of H5NX Bangladeshi sequences did suggest that similar patterns of intermixing of viral lineages between cities to those observed for H9N2 may exist, we could not repeat all analyses for H5 as for H9 due to a lack of available sequences and chicken type level metadata for the former subtype. Equally, while our H9N2 analyses focus mainly on sequences obtained from chickens, which are the hosts that H9N2 is more commonly found in and are the poultry type that accounts for more than 97 per cent of poultry moving through Dhaka and Chattogram’s market stalls (Moyen, 2021), HPAIV lineages such as H5N1 are relatively more frequently detected in ducks (Kim et al. 2018; Kwon et al. 2020). Differences in trading patterns between chickens and ducks may, therefore, result in possible divergent virus transmission dynamics between such AIV lineages (Moyen et al. 2018, 2021; Moyen 2019).

Our study has several limitations. First, virus genomic surveillance in Bangladesh is likely biased relative to infection distribution. Few sequences are available before 2013 in Dhaka or before 2016 in Chattogram, and change in genomic surveillance over time may therefore bias our results. There is a lack of AIV sequences from farms. Whilst this may be partly a true reflection of higher AIV prevalence at LBMs than at farms (Gupta et al. 2021; Ripa et al. 2021), this may also reflect surveillance bias towards LBMs, which are often in easier-to-reach locations than farms and where AIV is easier to detect. Finally, genomic sampling of H9N2 in Bangladesh is heavily biased towards Dhaka, and to a lesser extent, Chattogram, and therefore possible important sources and sinks of infection outside of these regions may be missed. Although H9 and H5 subtypes have been repeatedly detected in LBMs in both Dhaka and Chattogram (Sayeed et al. 2017; Biswas et al. 2018; Kim et al. 2018; Rimi et al. 2019; Hassan et al. 2020), the relatively lower number of Chattogram sequences may somewhat reflect both the higher number of live birds traded in Dhaka than in Chattogram (Moyen et al. 2021) as well as the greater quantity of mixed bird-type markets in Dhaka than Chattogram, which are generally associated with higher prevalence of infection (Kim et al. 2018). Biased sampling over space and time is less problematic for our analyses based on the cross-sectional dataset than those based on all publicly available data, as the cross-sectional dataset was generated through the same observational study conducted in both Chattogram and Dhaka. Our results show that subnational variation in production and trading processes may affect AIV dispersal between chicken types in Dhaka and Chattogram, and hence our results should be considered geographically specific and should not be extrapolated to other regions with production differences.

Second, for some analyses, we were limited to using only seventy sequences associated with known chicken types (Kim et al. 2018). This likely limited statistical power of our analyses of both market viral movement analyses and of city and chicken type clustering, relative to our analyses that did not use chicken type. Likewise, as these seventy sequences were generated in a single cross-sectional study (Kim et al. 2018) spanning only two months, we could not describe and explore seasonal variations in H9N2 infection patterns in such analyses. The dataset used for this analysis also contained only HA H9 sequences, meaning that we could not determine whether other segments or AIV subtypes may be similarly shaped by poultry trading practices in Bangladesh.

Improving insight into how viruses spread at a range of spatial scales could help guide improvements in AIV control. Our study shows the importance of recording accurate information on chicken type and highlights the need for greater surveillance on farms to understand viral epidemiology in Bangladesh. Our results suggest that nationally uniform interventions to reduce AIV prevalence may be unlikely to provide optimal effectiveness. Instead, actions should be tailored to the specific local structural characteristics of the poultry trading network and AIV dispersal patterns but could be made more efficient through targeted surveillance of a small number of key sites (Moyen et al. 2021). Any recommendations to improve AIV control in Bangladesh should adopt a multi-sector One Health approach to ensure proper consideration of health, social, and economic impacts (Chattopadhyay et al. 2018; Mackenzie and Jeggo 2019).

## Supplementary Material

vead014_Supp

## Data Availability

The datasets and BEAST XML files used in this study can be found at https://github.com/lorcancarnegie/H9N2_Bangladesh.git; GenBank Accession numbers for newly reported genetic sequence data are available under the accession numbers detailed in Table S1. Supplementary Data are available as separate files.

## References

[R1] Bi Y. , Li J., and Shi W. (2022) ‘The Time Is Now: A Call to Contain H9N2 Avian Influenza Viruses’, *The Lancet Microbe*, 3: E804–5. 36115378 10.1016/S2666-5247(22)00232-4

[R2] Biswas P. K. et al. (2018) ‘Incidence of Contamination of Live Bird Markets in Bangladesh with Influenza A Virus and Subtypes H5, H7 and H9’, *Transboundary and Emerging Diseases*, 65: 687–95. 29226568 10.1111/tbed.12788

[R3] Butts C. (2008) ‘Social Network Analysis with SNA’, *Journal of Statistical Software* 24: 1–51. 18618019 10.18637/jss.v024.i01 PMC2447931

[R4] Chakraborty D. et al. (2022) ‘Phylodynamic Analysis of the Highly Pathogenic Avian Influenza H5N8 Epidemic in France, 2016–17’, *Transboundary and Emerging Diseases*, 69: e1574–83. 35195353 10.1111/tbed.14490 PMC9790735

[R5] Chattopadhyay K. et al. (2018) ‘A Qualitative Stakeholder Analysis of Avian Influenza Policy in Bangladesh’, *EcoHealth*, 15: 63–71. 29134437 10.1007/s10393-017-1285-2 PMC6003964

[R6] Chen W.-C. (2011) ‘Overlapping Codon Model, Phylogenetic Clustering, and Alternative Partial Expectation Conditional Maximization Algorithm’, USA: Iowa State University.

[R8] Dellicour S. et al. (2020) ‘Epidemiological Hypothesis Testing Using a Phylogeographic and Phylodynamic Framework’, *Nature Communications*, 11: 5620. 10.1038/s41467-020-19122-z PMC7648063 33159066

[R9] Drummond A. J. et al. (2006) ‘Relaxed Phylogenetics and Dating with Confidence’, *PLoS Biology*, 4: e88. 10.1371/journal.pbio.0040088 PMC1395354 16683862

[R10] Drummond A. J. , and Rambaut A. (2007) ‘BEAST: Bayesian Evolutionary Analysis by Sampling Trees’, *BMC Evolutionary Biology*, 7: 214. 10.1186/1471-2148-7-214 PMC2247476 17996036

[R11] El-Shesheny R. et al. (2020) ‘Continued Evolution of H5Nx Avian Influenza Viruses in Bangladeshi Live Poultry Markets: Pathogenic Potential in Poultry and Mammalian Models’, *Journal of Virology*, 94: e01141-20. 32907981 10.1128/JVI.01141-20 PMC7654280

[R12] Fan Y. et al. (2011) ‘Choosing among Partition Models in Bayesian Phylogenetics’, *Molecular Biology and Evolution*, 28: 523–32. 20801907 10.1093/molbev/msq224 PMC3002242

[R13] Faria N. R. et al. (2013) ‘Simultaneously Reconstructing Viral Cross-species Transmission History and Identifying the Underlying Constraints’, *Philosophical Transactions of the Royal Society B: Biological Sciences*, 368: 20120196. 10.1098/rstb.2012.0196 PMC3678322 23382420

[R14] Food and Agriculture Organization of the United Nations . (2020) ‘FAOSTAT Statistical Database’, Rome: FAO, c1997.

[R15] Gerloff N. A. et al. (2013) ‘A High Diversity of Eurasian Lineage Low Pathogenicity Avian Influenza A Viruses Circulate among Wild Birds Sampled in Egypt’, *PLoS One*, 8: e68522–e68522. 23874653 10.1371/journal.pone.0068522 PMC3710070

[R16] Gerloff N. A. et al. (2016) ‘Genetically Diverse Low Pathogenicity Avian Influenza A Virus Subtypes Co-Circulate among Poultry in Bangladesh.’, *PLoS One*, 11: e0152131. 10.1371/journal.pone.0152131 PMC4806916 27010791

[R17] Gill M. S. et al. (2013) ‘Improving Bayesian Population Dynamics Inference: A Coalescent-based Model for Multiple Loci’, *Molecular Biology and Evolution*, 30: 713–24. 23180580 10.1093/molbev/mss265 PMC3563973

[R18] Gill M. S. et al. (2016) ‘Understanding past Population Dynamics: Bayesian Coalescent-based Modeling with Covariates’, *Systematic Biology*, 65: 1041–56. 27368344 10.1093/sysbio/syw050 PMC5066065

[R19] Griffiths R. C. , and Tavaré S. (1994) ‘Sampling Theory for Neutral Alleles in a Varying Environment’, *Philosophical Transactions of the Royal Society of London Series B, Biological Sciences*, 344: 403–10. 7800710 10.1098/rstb.1994.0079

[R20] Gupta S. D. et al. (2021) ‘Patterns of Avian Influenza A (H5) and A (H9) Virus Infection in Backyard, Commercial Broiler and Layer Chicken Farms in Bangladesh’, *Transboundary and Emerging Diseases*, 68: 137–51. 32639112 10.1111/tbed.13657

[R21] Hassan M. M. et al. (2020) ‘Prevalence and Diversity of Avian Influenza Virus Hemagglutinin Sero-Subtypes in Poultry and Wild Birds in Bangladesh’, *Veterinary Sciences*, 7: 73. 10.3390/vetsci7020073 PMC7355479 32492967

[R22] Høg E. et al. (2019) ‘Competing Biosecurity and Risk Rationalities in the Chittagong Poultry Commodity Chain, Bangladesh’, *BioSocieties*, 14: 368–92.

[R23] Høg E. et al. (2021) Avian Influenza Risk Environment: Live Bird Commodity Chains in Chattogram, Bangladesh. *Frontiers in Veterinary Science*, 8: 694753. 10.3389/fvets.2021.694753 PMC8489835 34616791

[R24] Kariithi H. M. et al. (2020) ‘Genetic Characterization and Pathogenesis of the First H9N2 Low Pathogenic Avian Influenza Viruses Isolated from Chickens in Kenyan Live Bird Markets’, *Infection, Genetics and Evolution: Journal of Molecular Epidemiology and Evolutionary Genetics in Infectious Diseases*, 78: 104074. 10.1016/j.meegid.2019.104074 31634645

[R25] Katoh K. et al. (2002) ‘MAFFT: A Novel Method for Rapid Multiple Sequence Alignment Based on Fast Fourier Transform’, *Nucleic Acids Research*, 30: 3059–66. 12136088 10.1093/nar/gkf436 PMC135756

[R26] Kim Y. et al. (2018) ‘Prevalence of Avian Influenza A(H5) and A(H9) Viruses in Live Bird Markets, Bangladesh’, *Emerging Infectious Diseases*, 24: 2309–16. 30457545 10.3201/eid2412.180879 PMC6256373

[R27] Kingman J. F. C. (1982) ‘The Coalescent’, *Stochastic Processes and Their Applications*, 13: 235–48.

[R28] Kwon J.-H. et al. (2020) ‘Genetic Evolution and Transmission Dynamics of Clade 2.3.2.1a Highly Pathogenic Avian Influenza A/H5N1 Viruses in Bangladesh’, *Virus Evolution*, 6: veaa046. 10.1093/ve/veaa046 PMC7474931 34127940

[R29] Lartillot N. , and Philippe H. (2006) ‘Computing Bayes Factors using Thermodynamic Integration’, *Systematic Biology*, 55: 195–207. 16522570 10.1080/10635150500433722

[R30] Lee Y.-N. et al. (2018) ‘Pathogenesis and Genetic Characteristics of Novel Reassortant Low-pathogenic Avian Influenza H7 Viruses Isolated from Migratory Birds in the Republic of Korea in the Winter of 2016-2017’, *Emerging Microbes & Infections*, 7: 182. 10.1038/s41426-018-0181-3 PMC6237977 30442892

[R31] Lu L. , Leigh Brown A. J., and Lycett S. J. (2017) ‘Quantifying Predictors for the Spatial Diffusion of Avian Influenza Virus in China’, *BMC Evolutionary Biology*, 17: 16. 10.1186/s12862-016-0845-3 PMC5237338 28086751

[R32] Lycett S. J. , Duchatel F., and Digard P. (2019) ‘A Brief History of Bird Flu’, *Philosophical Transactions of the Royal Society B: Biological Sciences*, 374: 20180257. 10.1098/rstb.2018.0257 PMC6553608 31056053

[R33] Mackenzie J. S. , and Jeggo M. (2019) ‘The One Health Approach—Why Is It So Important?’, *Tropical Medicine and Infectious Disease*, 4: 88. 10.3390/tropicalmed4020088 PMC6630404 31159338

[R34] Marco A. R. , and Ferreira M. A. S. (2008) ‘Bayesian Analysis of Elapsed Times in Continuous-time Markov Chains’, *Canadian Journal of Statistics*, 36: 355–68.

[R35] Marinova-Petkova A. et al. (2016) ‘The Continuing Evolution of H5N1 and H9N2 Influenza Viruses in Bangladesh between 2013 and 2014’, *Avian Diseases*, 60: 108–17. 27309046 10.1637/11136-050815-Reg PMC5479493

[R36] Moyen N. (2019) ‘The Potential Role of Live Bird Trade in Avian Influenza Virus Transmission in Bangladesh’, Royal Veterinary College, University of London.

[R37] Moyen N. et al. (2018) ‘A Large-scale Study of A Poultry Trading Network in Bangladesh: Implications for Control and Surveillance of Avian Influenza Viruses’, *BMC Veterinary Research*, 14: 12. 10.1186/s12917-018-1331-5 PMC5767022 29329534

[R38] Moyen N. et al. (2021) ‘Avian Influenza Transmission Risk along Live Poultry Trading Networks in Bangladesh’, *Scientific Reports*, 11: 19962. 10.1038/s41598-021-98989-4 PMC8497497 34620890

[R39] Paradis E. , and Schliep K. (2019) ‘Ape 5.0: An Environment for Modern Phylogenetics and Evolutionary Analyses in R’, *Bioinformatics*, 35: 526–8. 30016406 10.1093/bioinformatics/bty633

[R40] Parker J. , Rambaut A., and Pybus O. G. (2008) ‘Correlating Viral Phenotypes with Phylogeny: Accounting for Phylogenetic Uncertainty’, *Infection, Genetics and Evolution: Journal of Molecular Epidemiology and Evolutionary Genetics in Infectious Diseases*, 8: 239–46. 17921073 10.1016/j.meegid.2007.08.001

[R41] Parvin R. et al. (2018) ‘Review Analysis and Impact of Co-circulating H5N1 and H9N2 Avian Influenza Viruses in Bangladesh’, *Epidemiology and Infection*, 146: 1259–66. 29781424 10.1017/S0950268818001292 PMC9134290

[R42] Parvin R. et al. (2019) ‘Co-subsistence of Avian Influenza Virus Subtypes of Low and High Pathogenicity in Bangladesh: Challenges for Diagnosis, Risk Assessment and Control’, *Scientific Reports*, 9: 8306. 10.1038/s41598-019-44220-4 PMC6549172 31165743

[R43] Parvin R. et al. (2020) ‘Controlling Avian Influenza Virus in Bangladesh: Challenges and Recommendations’, *Viruses*, 12: 751. 10.3390/v12070751 PMC7412482 32664683

[R44] Price M. N. , Dehal P. S., and Arkin A. P. (2010) ‘FastTree 2—Approximately Maximum-likelihood Trees for Large Alignments’, *PLoS One*, 5: e9490. 10.1371/journal.pone.0009490 PMC2835736 20224823

[R45] Ragonnet-Cronin M. et al. (2013) ‘Automated Analysis of Phylogenetic Clusters’, *BMC Bioinformatics*, 14: 317. 10.1186/1471-2105-14-317 PMC4228337 24191891

[R46] Rambaut A. et al. (2016) ‘Exploring the Temporal Structure of Heterochronous Sequences Using TempEst (Formerly Path-O-Gen)’, *Virus Evolution*, 2: vew007. 10.1093/ve/vew007 PMC4989882 27774300

[R47] Rambaut A. et al. (2018) ‘Posterior Summarization in Bayesian Phylogenetics Using Tracer 1.7’, *Systematic Biology*, 67: 901–4. 29718447 10.1093/sysbio/syy032 PMC6101584

[R48] Rand W. M. (1971) ‘Objective Criteria for the Evaluation of Clustering Methods’, *Journal of the American Statistical Association*, 66: 846–50.

[R49] Rimi N. A. et al. (2019) ‘A Decade of Avian Influenza in Bangladesh: Where are We Now?’, *Tropical Medicine and Infectious Disease*, 4: 119. 10.3390/tropicalmed4030119 PMC6789720 31514405

[R50] Ripa R. N. et al. (2021) ‘Molecular Epidemiology and Pathogenicity of H5N1 and H9N2 Avian Influenza Viruses in Clinically Affected Chickens on Farms in Bangladesh’, *Emerging Microbes & Infections*, 10: 2223–34. 34753400 10.1080/22221751.2021.2004865 PMC8635652

[R51] RStudio Team (2020) ‘RStudio: Integrated Development for R. RStudio’, PBC, Boston, MA.

[R52] Sayeed M. A. et al. (2017) ‘Assessment of Hygienic Conditions of Live Bird Markets on Avian Influenza in Chittagong Metro, Bangladesh’, *Preventive Veterinary Medicine*, 142: 7–15. 28606367 10.1016/j.prevetmed.2017.04.009

[R53] Shanmuganatham K. et al. (2014) ‘Genesis of Avian Influenza H9N2 in Bangladesh’, *Emerging Microbes & Infections*, 3: e88. 10.1038/emi.2014.84 PMC4317637 26038507

[R54] Shapiro B. , Rambaut A., and Drummond A. J. (2006) ‘Choosing Appropriate Substitution Models for the Phylogenetic Analysis of Protein-coding Sequences’, *Molecular Biology and Evolution*, 23: 7–9. 16177232 10.1093/molbev/msj021

[R55] Shu Y. , and McCauley J. (2017) ‘GISAID: Global Initiative on Sharing All Influenza Data - from Vision to Reality’, *Eurosurveillance*, 22: 30494. 10.2807/1560-7917.ES.2017.22.13.30494 PMC5388101 28382917

[R56] Squires R. B. et al. (2012) ‘Influenza Research Database: An Integrated Bioinformatics Resource for Influenza Research and Surveillance’, *Influenza and Other Respiratory Viruses*, 6: 404–16. 22260278 10.1111/j.1750-2659.2011.00331.x PMC3345175

[R57] Stefan A. M. et al. (2019) ‘A Tutorial on Bayes Factor Design Analysis Using an Informed Prior’, *Behavior Research Methods*, 51: 1042–58. 30719688 10.3758/s13428-018-01189-8 PMC6538819

[R58] Suchard M. A. et al. (2018) ‘Bayesian Phylogenetic and Phylodynamic Data Integration Using BEAST 1.10’, *Virus Evolution*, 4: vey016. 10.1093/ve/vey016 PMC6007674 29942656

[R59] Tian H. et al. (2015) ‘Avian Influenza H5N1 Viral and Bird Migration Networks in Asia’, *Proceedings of the National Academy of Sciences of the United States of America*, 112: 172–7. 25535385 10.1073/pnas.1405216112 PMC4291667

[R60] Turner J. C. M. et al. (2017) ‘Insight into Live Bird Markets of Bangladesh: An Overview of the Dynamics of Transmission of H5N1 and H9N2 Avian Influenza Viruses’, *Emerging Microbes & Infections*, 6: e12. 10.1038/emi.2016.142 PMC5378921 28270655

[R61] Wu T. , and Perrings C. (2018) ‘The Live Poultry Trade and the Spread of Highly Pathogenic Avian Influenza: Regional Differences between Europe, West Africa, and Southeast Asia’, *PLoS One*, 13: e0208197. 10.1371/journal.pone.0208197 PMC6300203 30566454

[R62] Xie W. et al. (2011) ‘Improving Marginal Likelihood Estimation for Bayesian Phylogenetic Model Selection’, *Systematic Biology*, 60: 150–60. 21187451 10.1093/sysbio/syq085 PMC3038348

[R63] Yang J. et al. (2019) ‘Bayesian Phylodynamics of Avian Influenza A Virus H9N2 in Asia with Time-dependent Predictors of Migration’, *PLoS Computational Biology*, 15: e1007189. 10.1371/journal.pcbi.1007189 PMC6684064 31386651

[R64] Yang Q. et al. (2020) ‘Assessing the Role of Live Poultry Trade in Community-structured Transmission of Avian Influenza in China’, *Proceedings of the National Academy of Sciences*, 117: 5949–5954. 10.1073/pnas.1906954117 PMC7084072 32123088

[R65] Zeileis A. , and Grothendieck G. (2005) ‘Zoo: S3 Infrastructure for Regular and Irregular Time Series’, *Journal of Statistical Software*, 14: 1–27.

