# Supplementary material for: H9N2 avian influenza virus dispersal along Bangladeshi poultry trading networks
Source: Virus Evol. 2023 Feb 25;9(1):vead014. doi: 10.1093/ve/vead014 (PMC10032359; doi:10.1093/ve/vead014)
Supplement: vead014_Supp [file vead014_supp.zip › suppl_data/Supplementary_Data_Final.pdf]

Supplementary data

Table **S1.** GenBank accession numbers for newly generated HA (n = 111) and NA (n = 15) sequences [Separate file]

Table **S2.** GISAID acknowledgement table for HA (n = 211 H9N2, n = 175 H5) and NA (n = 200 H9N2, n = 172 H5) sequences. [Separate file]

Table **S3.** IRD acknowledgement table for HA (n = 115 H9N2, n = 132 H5) sequences and bird surveillance metadata. [Separate file]

Table **S4**. Accession numbers of 184 H9 sequences for which unpublished live bird market locations were obtained via Professor Richard Webby (St. Jude Research Center of Excellence for Influenza Research and Surveillance). [Separate file]

Table S5. Descriptions of predictors included in the discrete trait analyses with generalised linear models

Data	Source	Summary
Weekly Sales (Origin & Destination)	(Moyen 2019)	The estimated number of poultry sold through market vendors over a week in Dhaka and Chattogram.
Sample Size (Origin & Destination)	NA	Number of sequences per discrete trait (pairwise combination of city and chicken of sampling) in the 70 cross-sectional H9N2 dataset.
Chicken Similarity Index	NA	Square matrices of the six discrete traits (pairwise combination of city and chicken of sampling) in which 1 designated a shared chicken type host, and 0 designated a differing chicken type host.
City Similarity Index	NA	Square matrices of the six discrete traits (pairwise combination of city and chicken of sampling) in which 1 designated a shared city of sampling, and 0 designated a differing city of sampling.
Production Area Matrices	(Moyen et al. 2021)	The extent of overlap between production areas from which birds are sourced (1 - [Pianka index]; 0: production areas completely similar; 1: entirely dissimilar). The degree of overlap was based on reconstructed transaction networks, which themselves were estimated using trader interview data.

Table S6. Results of the adjusted Rand index analyses assessing the similarity of clustering by market and by genetically defined cluster for the cross-sectional H9N2 dataset.

	**Adjusted Rand Index**
Both Cities (statistic observed)	-0.031
Both Cities (median statistic permutation)	0.014
Both Cities (***p*** value)	0.993

Table S7. Results of the density analyses performed on the binary adjacency network generated using the cross-sectional H9N2 dataset.

	Density
Both Cities (statistic observed)	0.262
Both Cities (median statistic permutation)	0.314
Both Cities (***p*** value)	0.881

Table S8. Results of the BaTS v1.0 analysis assessing phylogeny-trait clustering by the city of sampling.

The 5% and 95% intervals for permuted statistics are included in brackets.

	PS	AI
City (statistic observed)	4	0.35
City (median statistic permutation)	22 (19 - 25)	3.83 (2.80 - 4.76)
City (***p*** value)	<0.001*	<0.001*

Table S9. Results of the BaTS v1.0 analysis assessing phylogeny-trait clustering by three chicken types, explored for each city separately. The 5% and 95% intervals for permuted statistics are included in brackets.

	PS	AI
Dhaka (statistic observed)	19	3.04
Dhaka (median statistic permutation)	20 (17-23)	3.29 (2.49 - 4.02)
Dhaka (***p*** value)	0.353	0.278
Chattogram (statistic observed)	10	1.30
Chattogram (median statistic permutation)	12 (10-14)	1.98 (1.33 - 2.51)
Chattogram (***p*** value)	0.071	0.036*

Table S10. Bayes factor results for the discrete trait analyses with generalised linear models.

	A	B	C	D
Origin Weekly Sales	2.8	11.0	NA	<1
Destination Weekly Sales	<1	1.2	NA	<1
Origin Sample Size	2.3	NA	8.8	1.3
Destination Sample Size	<1	NA	<1	<1
Catchment Area Matrices	27.8	79.4	53.4	<1
City Similarity Index	NA	NA	NA	>1000
Chicken Similarity Index	NA	NA	NA	<1

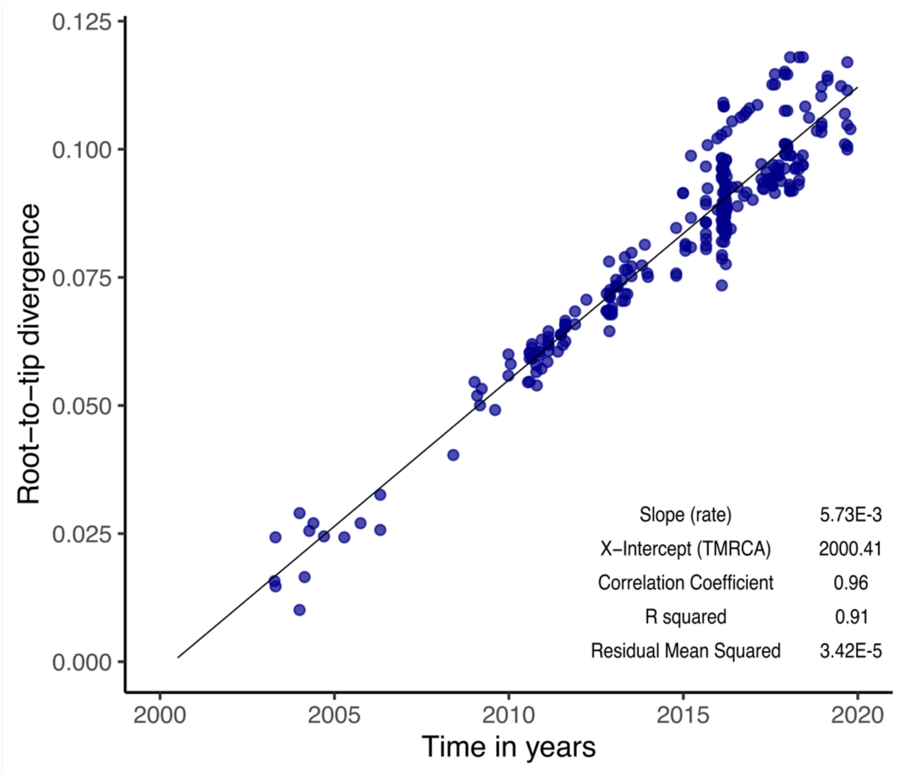

Figure S1. Root-to-tip divergence plots of the HA H9N2 dataset and associated statistics.

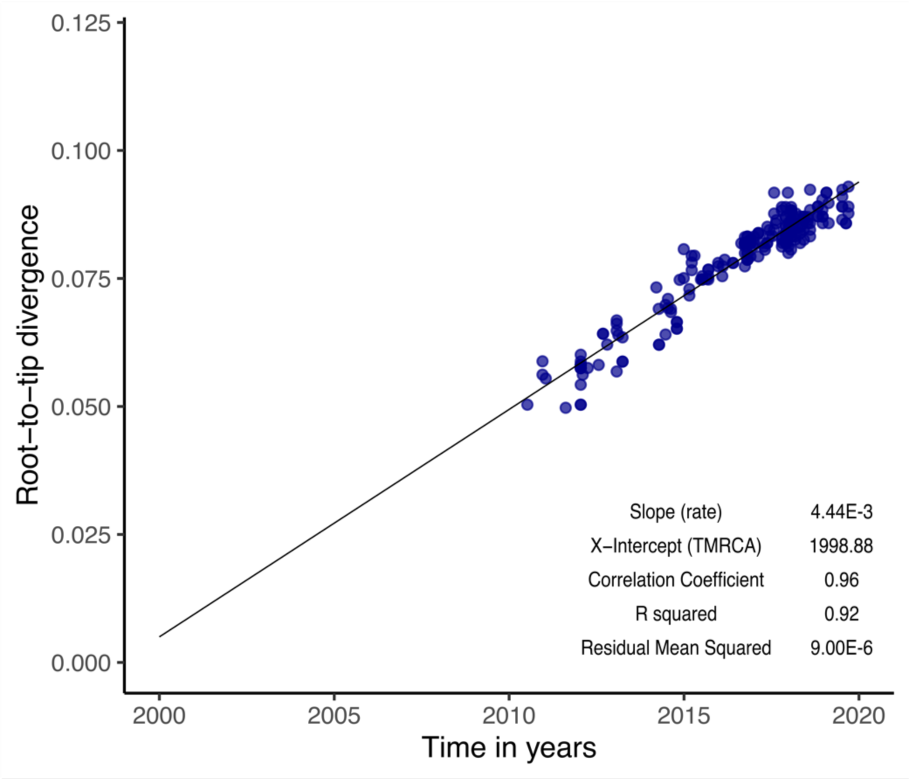

Figure S2. Root-to-tip divergence plots of the HA H5NX dataset and associated statistics.

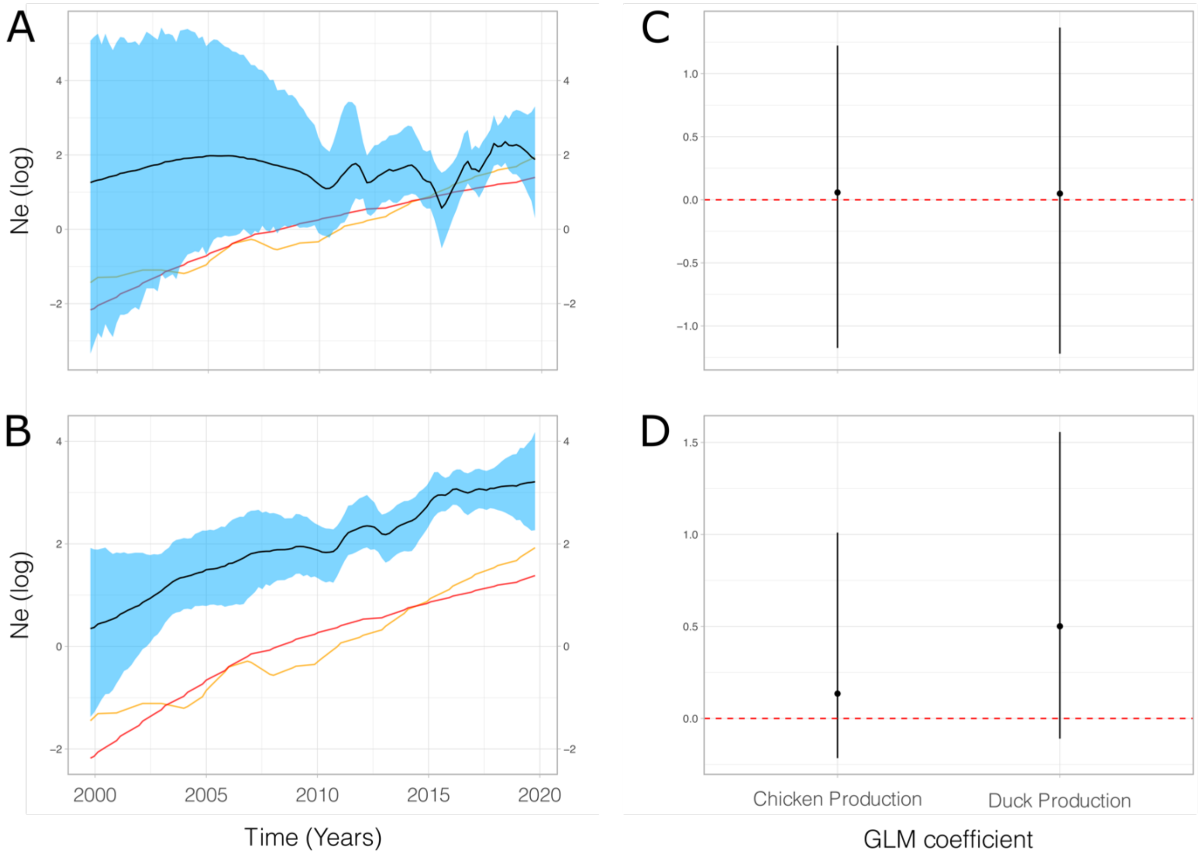

Figure S3. Skygrid plots showing the viral effective population size (N_e) of H5N1 (**A**) and H9N2 (**B**) through time, compared to estimated metrics of Bangladeshi chicken production (orange points) or duck production (red points) (log tonnes). The black line indicates the median N_e and the blue ribbon indicates the 95% highest posterior density values (HPD) intervals. **C** (H5N1) and **D** (H5N2) show mean GLM coefficient values (blue dots) and 95% HPD intervals (bars) for the two covariates.

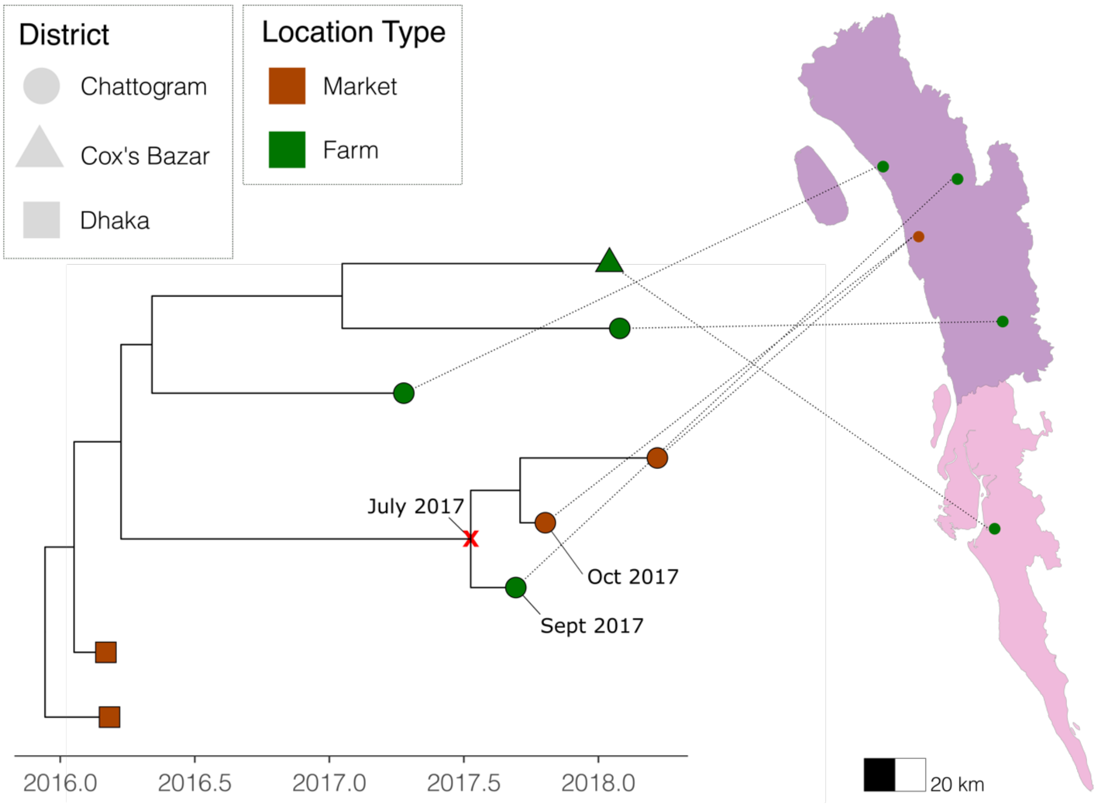

Figure S4. Extract of the HA H9N2 MCC tree showing phylogenetic evidence for potential virus movement between farms and LBMs. Tips are coloured by the sampling location and shaped by host species. The sampling location of the sequences is indicated on a map of Chattogram District and Cox's Bazar District.

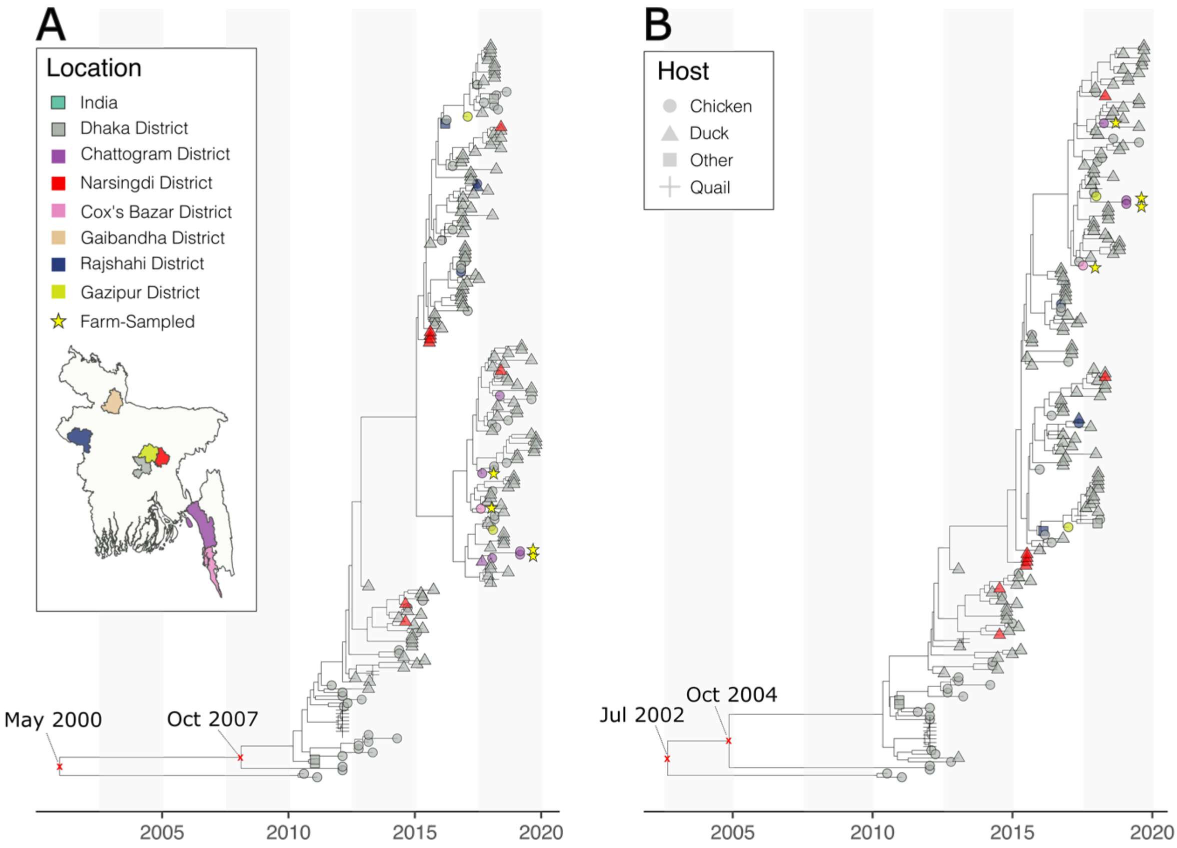

Figure S5. The estimated time-scaled MCC phylogenies of HA H5NX (**A**) and NA H5N1 (**B**). Tips are coloured by the sampling location as indicated on the map, and shaped by host type. Molecular clock phylogenies show that the time of the most recent common ancestor (TMRCA) for the Bangladeshi H5NX sequences circulating in Bangladesh was May 2000 (February 1994 – January 2006: 95% highest posterior density (HPD)) for the HA segment (**Figure S3A**), and July 2002 (January 2001 – March 2005: 95% highest posterior density (HPD)) for NA sequences (**Figure S3B**).

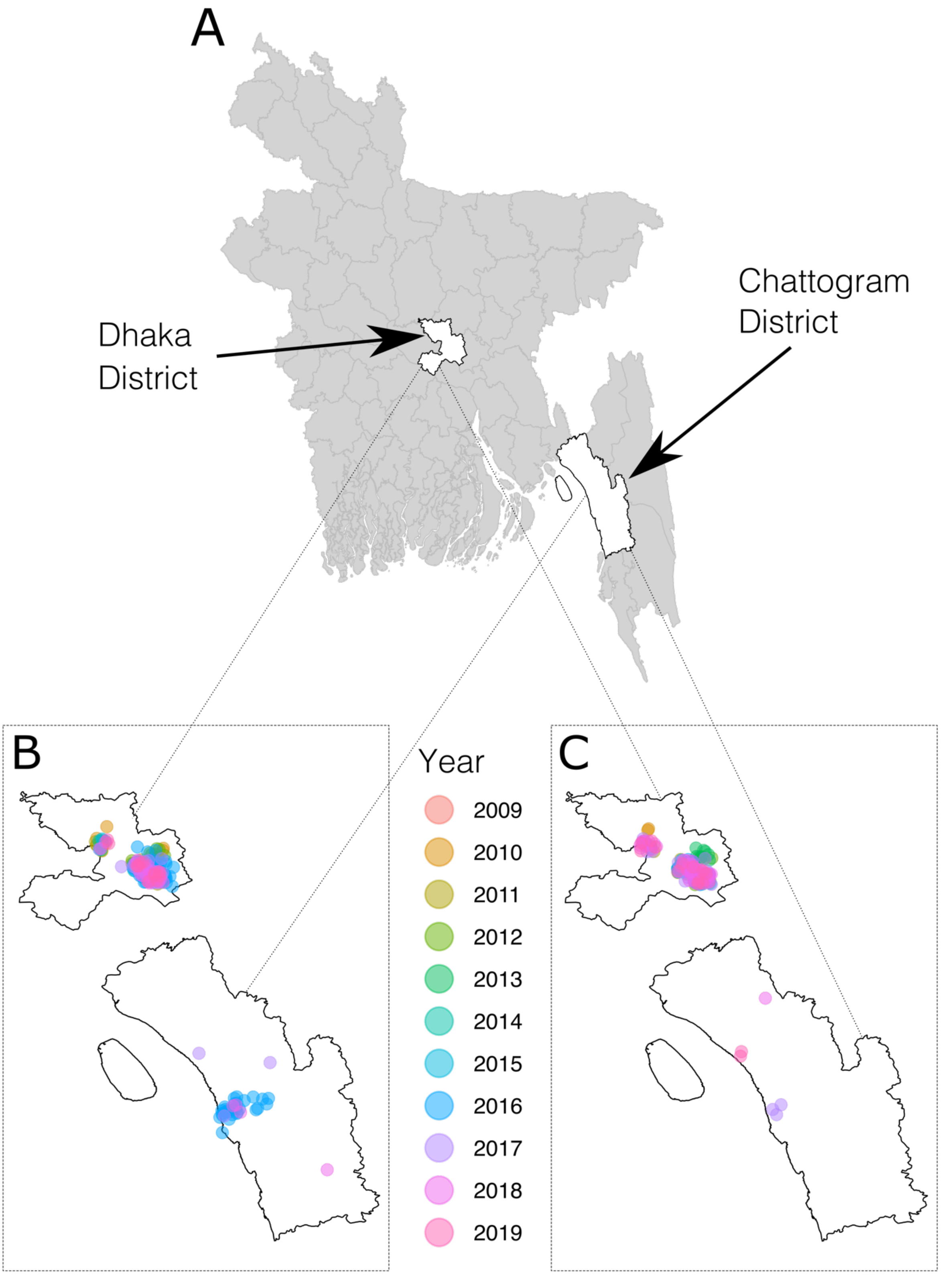

Figure S6. Maps of Bangladesh (**A**) detailing the sampling location of AIV (**B**; H9N2, C; H5NX) sequences are included here. Points are coloured by year of sampling, and Dhaka and Chattogram districts are

shaded in light grey and outlined in blue. A jitter of 0.025 degrees was applied to allow clearer display of samples from identical sampling locations. Districts in figures **B** and **C** are shown much closer than their true proximity for plotting purposes.

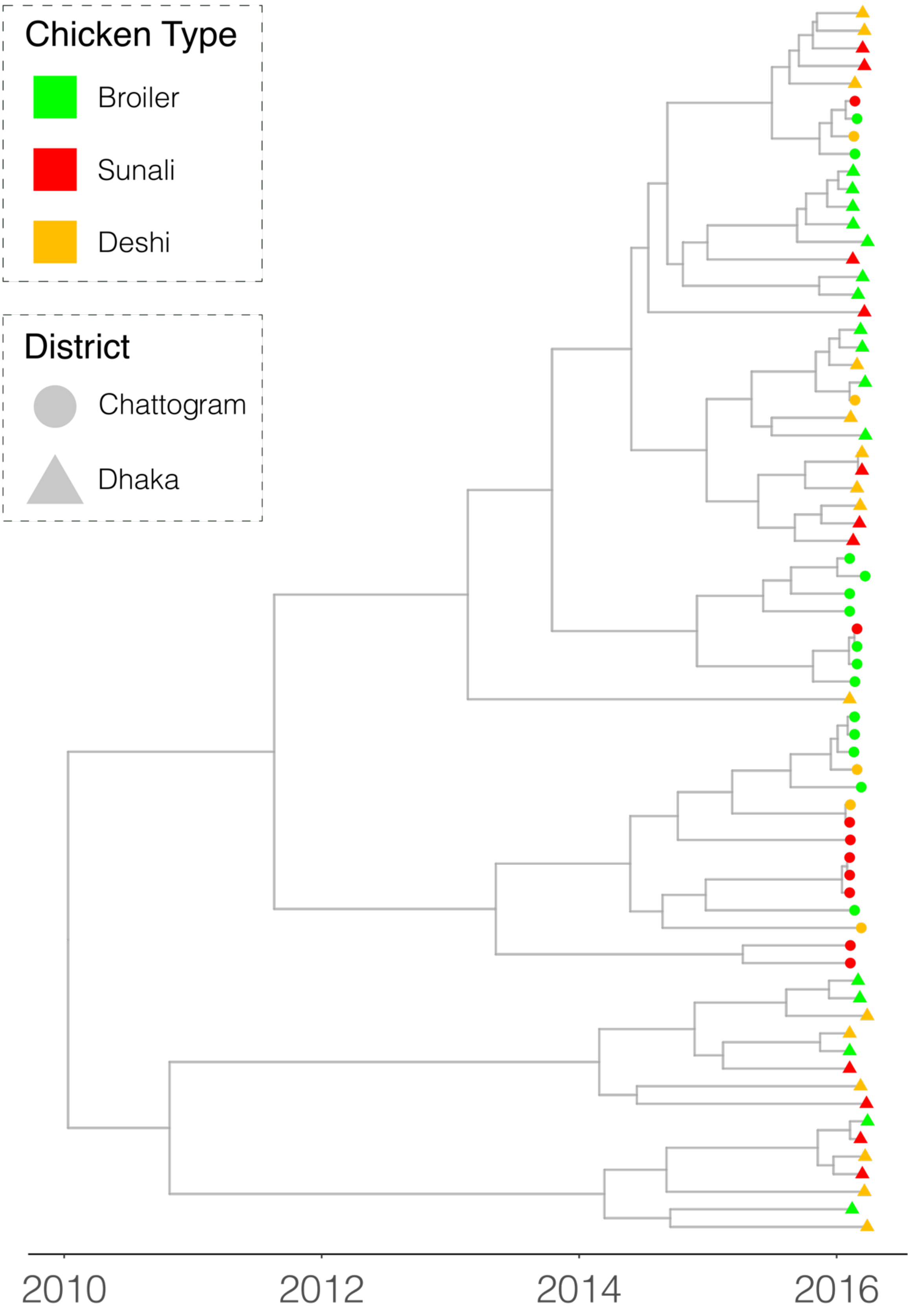

Figure S7. Molecular clock phylogeny of sequences from the cross-sectional H9N2 dataset extracted from the H9N2 MCC tree. Tips are coloured by the host chicken type and shaped by sampling location.

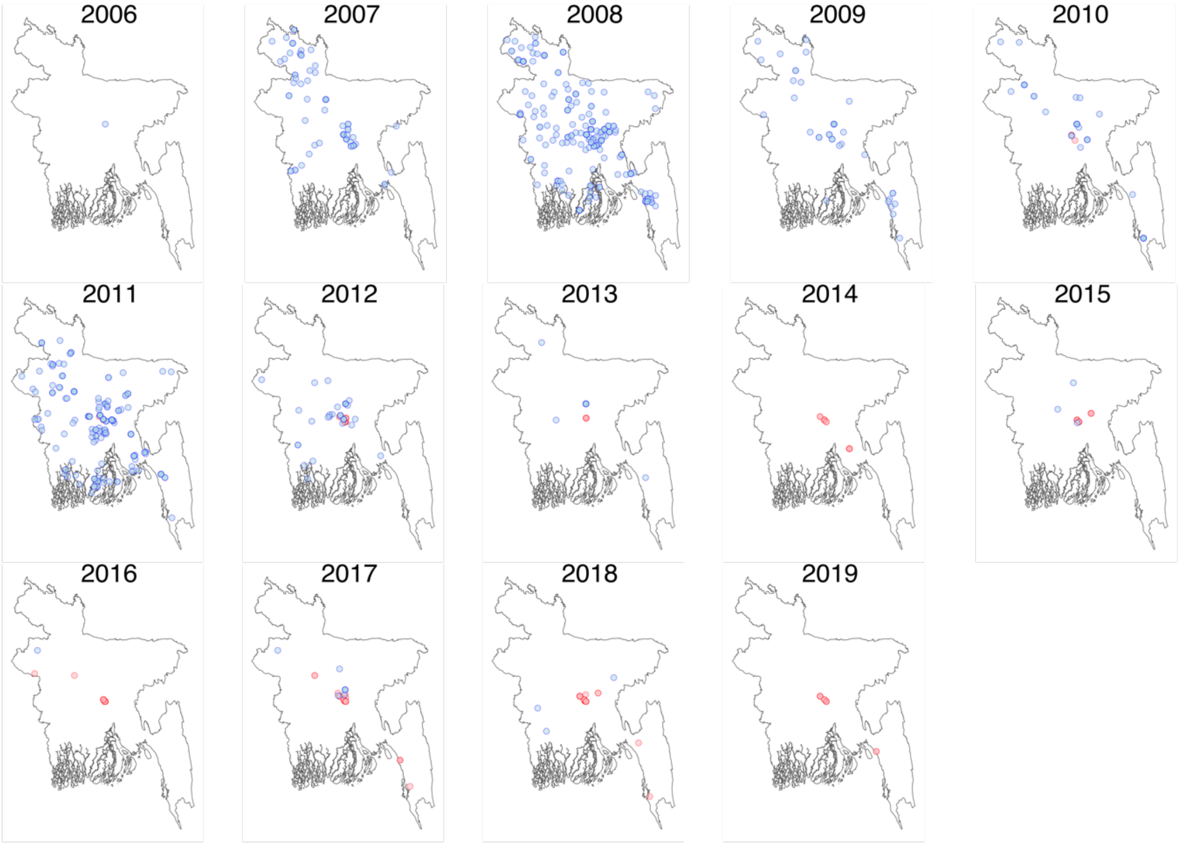

Figure S8. Maps of Bangladesh from 2006 to 2019 each containing i) all AIV confirmed infection locations, as recorded by the Global Animal Health Information System EMPRES-i of the FAO (blue points) (Claes et al. 2014), and ii) the sampling location for the H5NX sequences used in this study (red points).

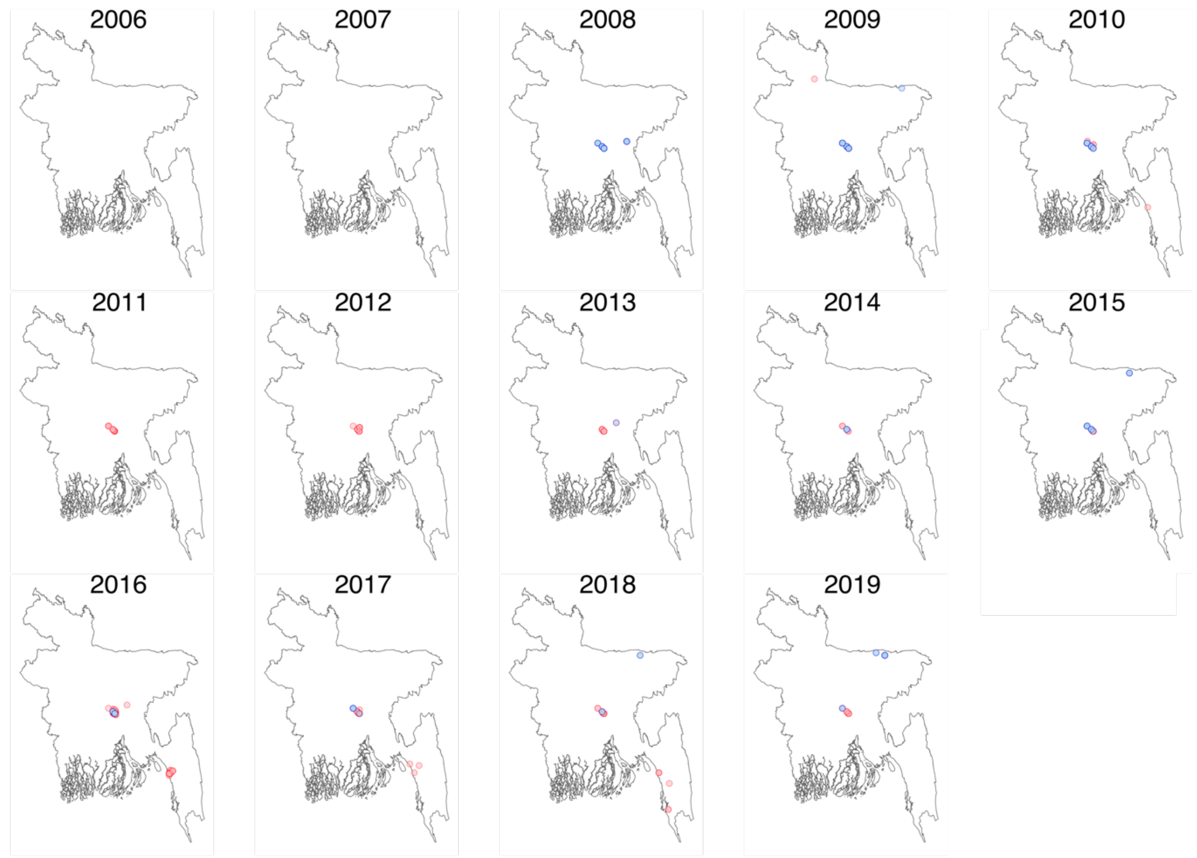

Figure S9. Maps of Bangladesh from 2006 to 2019 each containing i) the sampling location of H9NX and non-H9 LPAIV confirmed infections as recorded that year by recorded by the Influenza Research Database (IRD) (Squires et al. 2012) represented by blue points, and ii) the sampling location for the H9N2 sequences that were included in this study represented by red points.
