# Supplementary material for: H9N2 avian influenza virus dispersal along Bangladeshi poultry trading networks
Source: Virus Evol. 2023 Feb 25;9(1):vead014. doi: 10.1093/ve/vead014 (PMC10032359; doi:10.1093/ve/vead014)
Supplement: vead014_Supp [file vead014_supp.zip › TableS1.pdf]

#Accession	Subtype	Segment
OQ202254	H5	HA
OQ202255	H5	HA
OQ202256	H5	HA
OQ202257	H5	HA
OQ202258	H5	HA
OQ202259	H5	HA
OQ202260	H5	HA
OQ202261	H5	HA
OQ202262	H5	HA
OQ202263	H5	HA
OQ202264	H5	HA
OQ202265	H5	HA
OQ202266	H5	HA
OQ202267	H5	HA
OQ202268	H5	HA
OQ202269	H5	HA
OQ202270	H5	HA
OQ202271	H5	HA
OQ202272	H5	HA
OQ202273	H5	HA
OQ202274	H5	HA
OQ202275	H5	HA
OQ202276	H5	HA
OQ202277	H5	HA
OQ202278	H5	HA
OQ202279	H5	HA
OQ202280	H5	HA
OQ202281	H5	HA
OQ202282	H5	HA
OQ202238	H5	NA
OQ202239	H5	NA
OQ202240	H5	NA
OQ202241	H5	NA
OQ202242	H5	NA
OQ202243	H5	NA
OQ202244	H5	NA
OQ202245	H5	NA
OQ202246	H5	NA
OQ202247	H5	NA
OQ202248	H5	NA
OQ202249	H5	NA
OQ202250	H5	NA
OQ202251	H5	NA
OQ202252	H5	NA
OQ211613	H9	HA
OQ211614	H9	HA
OQ211615	H9	HA

OQ211616	H9	HA
OQ211617	H9	HA
OQ211618	H9	HA
OQ211619	H9	HA
OQ211620	H9	HA
OQ211621	H9	HA
OQ211622	H9	HA
OQ211623	H9	HA
OQ234982	H9	HA
OQ234983	H9	HA
OQ234984	H9	HA
OQ234985	H9	HA
OQ234986	H9	HA
OQ234987	H9	HA
OQ234988	H9	HA
OQ234989	H9	HA
OQ234990	H9	HA
OQ234991	H9	HA
OQ234992	H9	HA
OQ234993	H9	HA
OQ234994	H9	HA
OQ234995	H9	HA
OQ234996	H9	HA
OQ234997	H9	HA
OQ234998	H9	HA
OQ234999	H9	HA
OQ235000	H9	HA
OQ235001	H9	HA
OQ235002	H9	HA
OQ235003	H9	HA
OQ235004	H9	HA
OQ235005	H9	HA
OQ235006	H9	HA
OQ235007	H9	HA
OQ235008	H9	HA
OQ235009	H9	HA
OQ235010	H9	HA
OQ235011	H9	HA
OQ235012	H9	HA
OQ235013	H9	HA
OQ235014	H9	HA
OQ235015	H9	HA
OQ235016	H9	HA
OQ235017	H9	HA
OQ235018	H9	HA
OQ235019	H9	HA
OQ235020	H9	HA
OQ235021	H9	HA

OQ235022	H9	HA
OQ235023	H9	HA
OQ235024	H9	HA
OQ235025	H9	HA
OQ235026	H9	HA
OQ235027	H9	HA
OQ235028	H9	HA
OQ235029	H9	HA
OQ235030	H9	HA
OQ235031	H9	HA
OQ235032	H9	HA
OQ235033	H9	HA
OQ235034	H9	HA
OQ235035	H9	HA
OQ235036	H9	HA
OQ235037	H9	HA
OQ235038	H9	HA
OQ235039	H9	HA
OQ235040	H9	HA
OQ247794	H9	HA
OQ247795	H9	HA
OQ247796	H9	HA
OQ247797	H9	HA
OQ247798	H9	HA
OQ247799	H9	HA
OQ247800	H9	HA
OQ247801	H9	HA
OQ247802	H9	HA
OQ247803	H9	HA
OQ247804	H9	HA
OQ350862	H9	HA
