# Supplementary material for: H9N2 avian influenza virus dispersal along Bangladeshi poultry trading networks
Source: Virus Evol. 2023 Feb 25;9(1):vead014. doi: 10.1093/ve/vead014 (PMC10032359; doi:10.1093/ve/vead014)
Supplement: vead014_Supp [file vead014_supp.zip › TableS2.pdf]

GISAID acknowledgement table

We gratefully acknowledge the authors, originating and submitting laboratories of the sequences from GISAID's EpiFlu™ Database on which this research is based. The list is detailed below.

All submitters of data may be contacted directly via www.gisaid.org

Isolate-ID	Segment	Country	Collection date	Isolate name	Originating Lab	Subtype	Submitting Lab	Authors
EPI_ISL_257103	HA	Bangladesh	2015-Jan-23	A/quail/Bangladesh/24225/2015	NA	H9	Import from public-domain	Shanmuganatham,K.; Barman,S.; Marinova-Petkova,A.; Hasan,M.K.; Akhtar,S.; Turner,J.C.; Franks,J.; Walker,D.; Seiler,P.; Friedman,K.; Jones-Engel,L.; McKenzie,P.; Krauss,S.; Webby,R.J.; Feeroz,M.M.; Webster,R.G.
EPI_ISL_284715	HA	Bangladesh	2016-Dec-28	A/chicken/Bangladesh/31624/2016	NA	H9	Import from public-domain	Barman,S.; Turner,J.C.; Hasan,M.K.; Akhtar,S.; Franks,J.; El-Shesheny,R.; Walker,D.; Seiler,P.; Friedman,K.; Kercher,L.; Kayali,G.; Jones-Engel,L.; McKenzie,P.; Krauss,S.; Webby,R.J.; Feeroz,M.M.; Webster,R.G.
EPI_ISL_144264	HA	Bangladesh	2011-Aug-14	A/environment/Bangladesh/12116/2011	NA	H9	Import from public-domain	Shanmuganatham,K.; Feeroz,M.; Jones-Engel,L.; Smith,G.J.D.; Fourment,M.; Walker,D.; McClenaghan,L.; Rabiul Alam,S.M.; Hasan,K.; Seiler,P.; Franks,J.; Danner,A.; Barman,S.; McKenzie,P.; Krauss,S.; Webby,R.J.; Webster,R.G.; Engel,L.J.; Alam,S.M.R.; Ferguson,A.
EPI_ISL_306670	HA	Bangladesh	2017-Apr-19	A/quail/Bangladesh/32525/2017	NA	H9	Import from public-domain	Barman,S.; Turner,J.C.; Hasan,M.K.; Akhtar,S.; Franks,J.; El-Shesheny,R.; Walker,D.; Seiler,P.; Friedman,K.; Kercher,L.; Kayali,G.; Jones-Engel,L.; McKenzie,P.; Krauss,S.; Webby,R.J.; Feeroz,M.M.; Webster,R.G.
EPI_ISL_387985	HA	Bangladesh	2018-Dec-18	A/chicken/Bangladesh/35959/2018	NA	H9	Import from public-domain	Barman,S.; Turner,J.C.; Hasan,M.; Akhtar,S.; Franks,J.; El-Shesheny,R.; Walker,D.; Seiler,P.; Friedman,K.; Kercher,L.; McKenzie,P.; Webby,R.J.; Feeroz,M.; Webster,R.G.
EPI_ISL_379698	HA	Bangladesh	2017-Dec-25	A/environment/Bangladesh/34270/2017	NA	H9	Import from public-domain	Barman,S.; Turner,J.C.; Hasan,M.K.; Akhtar,S.; Franks,J.; El-Shesheny,R.; Walker,D.; Seiler,P.; Friedman,K.; Kercher,L.; Kayali,G.; Jones-Engel,L.; McKenzie,P.; Krauss,S.; Webby,R.J.; Feeroz,M.M.; Webster,R.G.
EPI_ISL_144255	HA	Bangladesh	2011-Feb-19	A/chicken/Bangladesh/10401/2011	NA	H9	Import from public-domain	Shanmuganatham,K.; Feeroz,M.; Jones-Engel,L.; Smith,G.J.D.; Fourment,M.; Walker,D.; McClenaghan,L.; Rabiul Alam,S.M.; Hasan,K.; Seiler,P.; Franks,J.; Danner,A.; Barman,S.; McKenzie,P.; Krauss,S.; Webby,R.J.; Webster,R.G.; Engel,L.J.; Alam,S.M.R.; Ferguson,A.
EPI_ISL_161228	HA	Bangladesh	2012-Nov-25	A/quail/Bangladesh/18514/2012	NA	H9	Import from public-domain	Shanmuganatham,K.; Feeroz,M.; Jones Engel,L.; Walker,D.; McClenaghan,L.; Alam,R.S.; Hasan,K.; McKenzie,P.; Webby,R.J.; Webster,R.G.
EPI_ISL_161238	HA	Bangladesh	2013-Jul-08	A/environment/Bangladesh/20199/2013	NA	H9	Import from public-domain	Shanmuganatham,K.; Feeroz,M.; Jones Engel,L.; Walker,D.; McClenaghan,L.; Alam,R.S.; Hasan,K.; McKenzie,P.; Webby,R.J.; Webster,R.G.
EPI_ISL_144279	HA	Bangladesh	2010-Nov-15	A/environment/Bangladesh/9306/2010	NA	H9	Import from public-domain	Shanmuganatham,K.; Feeroz,M.; Jones-Engel,L.; Smith,G.J.D.; Fourment,M.; Walker,D.; McClenaghan,L.; Rabiul Alam,S.M.; Hasan,K.; Seiler,P.; Franks,J.; Danner,A.; Barman,S.; McKenzie,P.; Krauss,S.; Webby,R.J.; Webster,R.G.; Engel,L.J.; Alam,S.M.R.; Ferguson,A.
EPI_ISL_257086	HA	Bangladesh	2015-Aug-25	A/environment/Bangladesh/25969/2015	NA	H9	Import from public-domain	Barman,S.; Marinova-Petkova,A.; Hasan,M.K.; Akhtar,S.; Turner,J.C.; Franks,J.; Walker,D.; Seiler,P.; Friedman,K.; Kercher,L.; Kayali,G.; Jones-Engel,L.; McKenzie,P.; Krauss,S.; Webby,R.J.; Feeroz,M.M.; Webster,R.G.
EPI_ISL_397430	HA	Bangladesh	2017-Jun-11	A/quail/Bangladesh/32935/2017	NA	H9	Import from public-domain	Barman,S.; Turner,J.C.; Hasan,M.K.; Akhtar,S.; Franks,J.; El-Shesheny,R.; Walker,D.; Seiler,P.; Friedman,K.; Kercher,L.; Kayali,G.; Jones-Engel,L.; McKenzie,P.; Krauss,S.; Webby,R.J.; Feeroz,M.; Webster,R.G.
EPI_ISL_144230	HA	Bangladesh	2011-Nov-24	A/chicken/Bangladesh/13916/2011	NA	H9	./	Shanmuganatham,K.; Feeroz,M.; Jones-Engel,L.; Smith,G.J.D.; Fourment,M.; Walker,D.; McClenaghan,L.; Rabiul Alam,S.M.; Hasan,K.; Seiler,P.; Franks,J.; Danner,A.; Barman,S.; McKenzie,P.; Krauss,S.; Webby,R.J.; Webster,R.G.; Engel,L.J.; Alam,S.M.R.; Ferguson,A.
EPI_ISL_284805	HA	Bangladesh	2016-Jul-22	A/chicken/Bangladesh/30457/2016	NA	H9	Import from public-domain	Barman,S.; Turner,J.C.; Hasan,M.K.; Akhtar,S.; Franks,J.; El-Shesheny,R.; Walker,D.; Seiler,P.; Friedman,K.; Kercher,L.; Kayali,G.; Jones-Engel,L.; McKenzie,P.; Krauss,S.; Webby,R.J.; Feeroz,M.M.; Webster,R.G.
EPI_ISL_144262	HA	Bangladesh	2011-Aug-14	A/environment/Bangladesh/12119/2011	NA	H9	Import from public-domain	Shanmuganatham,K.; Feeroz,M.; Jones-Engel,L.; Smith,G.J.D.; Fourment,M.; Walker,D.; McClenaghan,L.; Rabiul Alam,S.M.; Hasan,K.; Seiler,P.; Franks,J.; Danner,A.; Barman,S.; McKenzie,P.; Krauss,S.; Webby,R.J.; Webster,R.G.; Engel,L.J.; Alam,S.M.R.; Ferguson,A.
EPI_ISL_257059	HA	Bangladesh	2015-Aug-27	A/chicken/Bangladesh/26120/2015	NA	H9	Import from public-domain	Barman,S.; Marinova-Petkova,A.; Hasan,M.K.; Akhtar,S.; Turner,J.C.; Franks,J.; Walker,D.; Seiler,P.; Friedman,K.; Kercher,L.; Kayali,G.; Jones-Engel,L.; McKenzie,P.; Krauss,S.; Webby,R.J.; Feeroz,M.M.; Webster,R.G.
EPI_ISL_503486	HA	Bangladesh	2019-Sep-13	A/quail/Bangladesh/40847/2019	NA	H9	Import from public-domain	Barman,S.; Turner,J.C.; Hasan,M.; Akhtar,S.; Franks,J.; El-Shesheny,R.; Walker,D.; Seiler,P.; Mukherjee,N.; Kercher,L.; McKenzie,P.; Feeroz,M.; Webby,R.J.
EPI_ISL_144231	HA	Bangladesh	2011-Aug-14	A/environment/Bangladesh/12068/2011	NA	H9	Import from public-domain	Shanmuganatham,K.; Feeroz,M.; Jones-Engel,L.; Smith,G.J.D.; Fourment,M.; Walker,D.; McClenaghan,L.; Rabiul Alam,S.M.; Hasan,K.; Seiler,P.; Franks,J.; Danner,A.; Barman,S.; McKenzie,P.; Krauss,S.; Webby,R.J.; Webster,R.G.; Engel,L.J.; Alam,S.M.R.; Ferguson,A.
EPI_ISL_161237	HA	Bangladesh	2013-Jan-28	A/environment/Bangladesh/18846/2013	NA	H9	Import from public-domain	Shanmuganatham,K.; Feeroz,M.; Jones Engel,L.; Walker,D.; McClenaghan,L.; Alam,R.S.; Hasan,K.; McKenzie,P.; Webby,R.J.; Webster,R.G.
EPI_ISL_503515	HA	Bangladesh	2019-Sep-13	A/chicken/Bangladesh/40876/2019	NA	H9	Import from public-domain	Barman,S.; Turner,J.C.; Hasan,M.; Akhtar,S.; Franks,J.; El-Shesheny,R.; Walker,D.; Seiler,P.; Mukherjee,N.; Kercher,L.; McKenzie,P.; Feeroz,M.; Webby,R.J.
EPI_ISL_165814	HA	Bangladesh	2010-Oct-20	A/environment/Bangladesh/155/2010	Institute of Epidemiology Disease Control and Research (IEDCR) & Bangladesh National Influenza Centre (NIC)	H9	Centers for Disease Control and Prevention	Gerloff, Nancy; Simpson, Natosha; Davis, C. Todd
EPI_ISL_503529	HA	Bangladesh	2019-Sep-13	A/chicken/Bangladesh/40818/2019	NA	H9	Import from public-domain	Barman,S.; Turner,J.C.; Hasan,M.; Akhtar,S.; Franks,J.; El-Shesheny,R.; Walker,D.; Seiler,P.; Mukherjee,N.; Kercher,L.; McKenzie,P.; Feeroz,M.; Webby,R.J.
EPI_ISL_331118	HA	Bangladesh	2016-May-12	A/layer chicken/Bangladesh/VP02-Plaque purified H9A/2016	Bangladesh Agricultural University, Faculty of Veterinary Science, Department of Pathology	H9	Friedrich-Loeffler-Institut	Parvin, Rokshana; Begum, Jahan Ara; Chowdhury, Emadadul Haque; Islam, Mohammed Rafiqul; Beer, Martin; Harder, Timm C
EPI_ISL_257102	HA	Bangladesh	2015-Mar-22	A/quail/Bangladesh/24922/2015	NA	H9	Import from public-domain	Shanmuganatham,K.; Barman,S.; Marinova-Petkova,A.; Hasan,M.K.; Akhtar,S.; Turner,J.C.; Franks,J.; Walker,D.; Seiler,P.; Friedman,K.; Jones-Engel,L.; McKenzie,P.; Krauss,S.; Webby,R.J.; Feeroz,M.M.; Webster,R.G.
EPI_ISL_165817	HA	Bangladesh	2012-Dec-15	A/poultry/Bangladesh/91349/2012	Institute of Epidemiology Disease Control and Research (IEDCR) & Bangladesh National Influenza Centre (NIC)	H9	Centers for Disease Control and Prevention	Gerloff, Nancy; Simpson, Natosha; Davis, C. Todd
EPI_ISL_161200	HA	Bangladesh	2013-Jan-28	A/environment/Bangladesh/18849/2013	NA	H9	Import from public-domain	Shanmuganatham,K.; Feeroz,M.; Jones Engel,L.; Walker,D.; McClenaghan,L.; Alam,R.S.; Hasan,K.; McKenzie,P.; Webby,R.J.; Webster,R.G.
EPI_ISL_144260	HA	Bangladesh	2011-Feb-19	A/environment/Bangladesh/10316/2011	NA	H9	Import from public-domain	Shanmuganatham,K.; Feeroz,M.; Jones-Engel,L.; Smith,G.J.D.; Fourment,M.; Walker,D.; McClenaghan,L.; Rabiul Alam,S.M.; Hasan,K.; Seiler,P.; Franks,J.; Danner,A.; Barman,S.; McKenzie,P.; Krauss,S.; Webby,R.J.; Webster,R.G.; Engel,L.J.; Alam,S.M.R.; Ferguson,A.
EPI_ISL_379688	HA	Bangladesh	2017-Nov-28	A/environment/Bangladesh/34022/2017	NA	H9	Import from public-domain	Barman,S.; Turner,J.C.; Hasan,M.K.; Akhtar,S.; Franks,J.; El-Shesheny,R.; Walker,D.; Seiler,P.; Friedman,K.; Kercher,L.; Kayali,G.; Jones-Engel,L.; McKenzie,P.; Krauss,S.; Webby,R.J.; Feeroz,M.M.; Webster,R.G.
EPI_ISL_503518	HA	Bangladesh	2019-Jul-10	A/chicken/Bangladesh/40498/2019	NA	H9	Import from public-domain	Barman,S.; Turner,J.C.; Hasan,M.; Akhtar,S.; Franks,J.; El-Shesheny,R.; Walker,D.; Seiler,P.; Mukherjee,N.; Kercher,L.; McKenzie,P.; Feeroz,M.; Webby,R.J.
EPI_ISL_388024	HA	Bangladesh	2018-Apr-26	A/chicken/Bangladesh/35278/2018	NA	H9	Import from public-domain	Barman,S.; Turner,J.C.; Hasan,M.; Akhtar,S.; Franks,J.; El-Shesheny,R.; Walker,D.; Seiler,P.; Friedman,K.; Kercher,L.; McKenzie,P.; Webby,R.J.; Feeroz,M.; Webster,R.G.
EPI_ISL_257051	HA	Bangladesh	2015-Aug-27	A/chicken/Bangladesh/26102/2015	NA	H9	Import from public-domain	Barman,S.; Marinova-Petkova,A.; Hasan,M.K.; Akhtar,S.; Turner,J.C.; Franks,J.; Walker,D.; Seiler,P.; Friedman,K.; Kercher,L.; Kayali,G.; Jones-Engel,L.; McKenzie,P.; Krauss,S.; Webby,R.J.; Feeroz,M.M.; Webster,R.G.
EPI_ISL_161231	HA	Bangladesh	2012-Nov-15	A/chicken/Bangladesh/18408/2012	NA	H9	Import from public-domain	Shanmuganatham,K.; Feeroz,M.; Jones Engel,L.; Walker,D.; McClenaghan,L.; Alam,R.S.; Hasan,K.; McKenzie,P.; Webby,R.J.; Webster,R.G.
EPI_ISL_329589	HA	Bangladesh	2017-Jul-21	A/duck/Bangladesh/33135/2017	NA	H9	Import from public-domain	Barman,S.; Turner,J.C.; Hasan,M.K.; Akhtar,S.; Franks,J.; El-Shesheny,R.; Walker,D.; Seiler,P.; Friedman,K.; Kercher,L.; Kayali,G.; Jones-Engel,L.; McKenzie,P.; Krauss,S.; Webby,R.J.; Feeroz,M.; Webster,R.G.
EPI_ISL_329579	HA	Bangladesh	2017-Jul-21	A/duck/Bangladesh/33127/2017	NA	H9	Import from public-domain	Barman,S.; Turner,J.C.; Hasan,M.K.; Akhtar,S.; Franks,J.; El-Shesheny,R.; Walker,D.; Seiler,P.; Friedman,K.; Kercher,L.; Kayali,G.; Jones-Engel,L.; McKenzie,P.; Krauss,S.; Webby,R.J.; Feeroz,M.; Webster,R.G.
EPI_ISL_503496	HA	Bangladesh	2019-Sep-14	A/chicken/Bangladesh/40926/2019	NA	H9	Import from public-domain	Barman,S.; Turner,J.C.; Hasan,M.; Akhtar,S.; Franks,J.; El-Shesheny,R.; Walker,D.; Seiler,P.; Mukherjee,N.; Kercher,L.; McKenzie,P.; Feeroz,M.; Webby,R.J.

EPI_ISL_165818	HA	Bangladesh	2012-Nov-19	A/avian/Bangladesh/91256/2012	Institute of Epidemiology Disease Control and Research (IEDCR) & Bangladesh National Influenza Centre (NIC)	H9	Centers for Disease Control and Prevention	Gerloff, Nancy; Simpson, Natosha; Davis, C. Todd
EPI_ISL_256786	HA	Bangladesh	2014-Oct-19	A/chicken/Bangladesh/23740/2014	NA	H9	Import from public-domain	Shanmuganatham,K.; Barman,S.; Marinova-Petkova,A.; Hasan,M.K.; Akhtar,S.; Turner,J.C.; Franks,J.; Walker,D.; Seiler,P.; Friedman,K.; Jones-Engel,L.; McKenzie,P.; Krauss,S.; Webby,R.J.; Feeroz,M.M.; Webster,R.G.
EPI_ISL_257081	HA	Bangladesh	2015-Sep-12	A/environment/Bangladesh/26218/2015	NA	H9	Import from public-domain	Barman,S.; Marinova-Petkova,A.; Hasan,M.K.; Akhtar,S.; Turner,J.C.; Franks,J.; Walker,D.; Seiler,P.; Friedman,K.; Kercher,L.; Kayali,G.; Jones-Engel,L.; McKenzie,P.; Krauss,S.; Webby,R.J.; Feeroz,M.M.; Webster,R.G.
EPI_ISL_257063	HA	Bangladesh	2015-Aug-25	A/chicken/Bangladesh/25945/2015	NA	H9	Import from public-domain	Barman,S.; Marinova-Petkova,A.; Hasan,M.K.; Akhtar,S.; Turner,J.C.; Franks,J.; Walker,D.; Seiler,P.; Friedman,K.; Kercher,L.; Kayali,G.; Jones-Engel,L.; McKenzie,P.; Krauss,S.; Webby,R.J.; Feeroz,M.M.; Webster,R.G.
EPI_ISL_144235	HA	Bangladesh	2011-Feb-19	A/chicken/Bangladesh/10450/2011	NA	H9	Import from public-domain	Shanmuganatham,K.; Feeroz,M.; Jones-Engel,L.; Smith,G.J.D.; Fourment,M.; Walker,D.; McClenaghan,L.; Rabiul Alam,S.M.; Hasan,K.; Seiler,P.; Franks,J.; Danner,A.; Barman,S.; McKenzie,P.; Krauss,S.; Webby,R.J.; Webster,R.G.; Engel,L.J.; Alam,S.M.R.; Ferguson,A.
EPI_ISL_161218	HA	Bangladesh	2013-Jul-08	A/quail/Bangladesh/20177/2013	NA	H9	Import from public-domain	Shanmuganatham,K.; Feeroz,M.; Jones Engel,L.; Walker,D.; McClenaghan,L.; Alam,R.S.; Hasan,K.; McKenzie,P.; Webby,R.J.; Webster,R.G.
EPI_ISL_379701	HA	Bangladesh	2017-Dec-25	A/chicken/Bangladesh/34322/2017	NA	H9	Import from public-domain	Barman,S.; Turner,J.C.; Hasan,M.K.; Akhtar,S.; Franks,J.; El-Shesheny,R.; Walker,D.; Seiler,P.; Friedman,K.; Kercher,L.; Kayali,G.; Jones-Engel,L.; McKenzie,P.; Krauss,S.; Webby,R.J.; Feeroz,M.M.; Webster,R.G.
EPI_ISL_257085	HA	Bangladesh	2015-Dec-23	A/quail/Bangladesh/27835/2015	NA	H9	Import from public-domain	Barman,S.; Marinova-Petkova,A.; Hasan,M.K.; Akhtar,S.; Turner,J.C.; Franks,J.; Walker,D.; Seiler,P.; Friedman,K.; Kercher,L.; Kayali,G.; Jones-Engel,L.; McKenzie,P.; Krauss,S.; Webby,R.J.; Feeroz,M.M.; Webster,R.G.
EPI_ISL_329577	HA	Bangladesh	2017-Jul-21	A/chicken/Bangladesh/33112/2017	NA	H9	Import from public-domain	Barman,S.; Turner,J.C.; Hasan,M.K.; Akhtar,S.; Franks,J.; El-Shesheny,R.; Walker,D.; Seiler,P.; Friedman,K.; Kercher,L.; Kayali,G.; Jones-Engel,L.; McKenzie,P.; Krauss,S.; Webby,R.J.; Feeroz,M.; Webster,R.G.
EPI_ISL_284713	HA	Bangladesh	2016-Mar-28	A/chicken/Bangladesh/29588/2016	NA	H9	Import from public-domain	Barman,S.; Turner,J.C.; Hasan,M.K.; Akhtar,S.; Franks,J.; El-Shesheny,R.; Walker,D.; Seiler,P.; Friedman,K.; Kercher,L.; Kayali,G.; Jones-Engel,L.; McKenzie,P.; Krauss,S.; Webby,R.J.; Feeroz,M.M.; Webster,R.G.
EPI_ISL_144265	HA	Bangladesh	2009-Mar-22	A/chicken/Bangladesh/2075/2009	NA	H9	Import from public-domain	Shanmuganatham,K.; Feeroz,M.; Jones-Engel,L.; Smith,G.J.D.; Fourment,M.; Walker,D.; McClenaghan,L.; Rabiul Alam,S.M.; Hasan,K.; Seiler,P.; Franks,J.; Danner,A.; Barman,S.; McKenzie,P.; Krauss,S.; Webby,R.J.; Webster,R.G.; Engel,L.J.; Alam,S.M.R.; Ferguson,A.
EPI_ISL_27600	HA	India	2003-Apr-24	A/chicken/Haryana/2051/2003	NA	H9	Import from public-domain	
EPI_ISL_284798	HA	Bangladesh	2016-Aug-21	A/quail/Bangladesh/30637/2016	NA	H9	Import from public-domain	Barman,S.; Turner,J.C.; Hasan,M.K.; Akhtar,S.; Franks,J.; El-Shesheny,R.; Walker,D.; Seiler,P.; Friedman,K.; Kercher,L.; Kayali,G.; Jones-Engel,L.; McKenzie,P.; Krauss,S.; Webby,R.J.; Feeroz,M.M.; Webster,R.G.
EPI_ISL_69121	HA	India	2005-Oct-01	A/watercoot/Haryana/5844/2005	NA	H9	Import from public-domain	Nagarajan,S.; Jain,R.; Gounalan,S.; Tosh,C.; Pradhan,H.K.; Dubey,S.C.
EPI_ISL_83300	HA	India	2008-May-29	A/chicken/Tripura/105131/2008	NA	H9	Import from public-domain	Nagarajan,S.; Tripati,S.; Tosh,C.; Murugkar,H.V.; Dubey,S.C.
EPI_ISL_161227	HA	Bangladesh	2012-Oct-21	A/environment/Bangladesh/18317/2012	NA	H9	Import from public-domain	Shanmuganatham,K.; Feeroz,M.; Jones Engel,L.; Walker,D.; McClenaghan,L.; Alam,R.S.; Hasan,K.; McKenzie,P.; Webby,R.J.; Webster,R.G.
EPI_ISL_329578	HA	Bangladesh	2017-Jul-21	A/chicken/Bangladesh/33107/2017	NA	H9	Import from public-domain	Barman,S.; Turner,J.C.; Hasan,M.K.; Akhtar,S.; Franks,J.; El-Shesheny,R.; Walker,D.; Seiler,P.; Friedman,K.; Kercher,L.; Kayali,G.; Jones-Engel,L.; McKenzie,P.; Krauss,S.; Webby,R.J.; Feeroz,M.; Webster,R.G.
EPI_ISL_379731	HA	Bangladesh	2018-Jan-23	A/chicken/Bangladesh/34637/2018	NA	H9	Import from public-domain	Barman,S.; Turner,J.C.; Hasan,M.K.; Akhtar,S.; Franks,J.; El-Shesheny,R.; Walker,D.; Seiler,P.; Friedman,K.; Kercher,L.; Kayali,G.; Jones-Engel,L.; McKenzie,P.; Krauss,S.; Webby,R.J.; Feeroz,M.M.; Webster,R.G.
EPI_ISL_161687	HA	Bangladesh	2013-Dec-27	A/quail/Bangladesh/21483/2013	NA	H9	Import from public-domain	Shanmuganatham,K.; Feeroz,M.; Jones Engel,L.; Walker,D.; McClenaghan,L.; Alam,R.S.; Hasan,K.; McKenzie,P.; Webby,R.J.; Webster,R.G.
EPI_ISL_161202	HA	Bangladesh	2012-Nov-25	A/quail/Bangladesh/18521/2012	NA	H9	Import from public-domain	Shanmuganatham,K.; Feeroz,M.; Jones Engel,L.; Walker,D.; McClenaghan,L.; Alam,R.S.; Hasan,K.; McKenzie,P.; Webby,R.J.; Webster,R.G.
EPI_ISL_161201	HA	Bangladesh	2012-Oct-21	A/environment/Bangladesh/18315/2012	NA	H9	Import from public-domain	Shanmuganatham,K.; Feeroz,M.; Jones Engel,L.; Walker,D.; McClenaghan,L.; Alam,R.S.; Hasan,K.; McKenzie,P.; Webby,R.J.; Webster,R.G.
EPI_ISL_329593	HA	Bangladesh	2017-Jun-11	A/chicken/Bangladesh/32957/2017	NA	H9	Import from public-domain	Barman,S.; Turner,J.C.; Hasan,M.K.; Akhtar,S.; Franks,J.; El-Shesheny,R.; Walker,D.; Seiler,P.; Friedman,K.; Kercher,L.; Kayali,G.; Jones-Engel,L.; McKenzie,P.; Krauss,S.; Webby,R.J.; Feeroz,M.; Webster,R.G.
EPI_ISL_144258	HA	Bangladesh	2011-Aug-14	A/environment/Bangladesh/12093/2011	NA	H9	Import from public-domain	Shanmuganatham,K.; Feeroz,M.; Jones-Engel,L.; Smith,G.J.D.; Fourment,M.; Walker,D.; McClenaghan,L.; Rabiul Alam,S.M.; Hasan,K.; Seiler,P.; Franks,J.; Danner,A.; Barman,S.; McKenzie,P.; Krauss,S.; Webby,R.J.; Webster,R.G.; Engel,L.J.; Alam,S.M.R.; Ferguson,A.
EPI_ISL_379972	HA	Bangladesh	2018-Jan-23	A/quail/Bangladesh/34615/2018	NA	H9	Import from public-domain	Barman,S.; Turner,J.C.; Hasan,M.K.; Akhtar,S.; Franks,J.; El-Shesheny,R.; Walker,D.; Seiler,P.; Friedman,K.; Kercher,L.; Kayali,G.; Jones-Engel,L.; McKenzie,P.; Krauss,S.; Webby,R.J.; Feeroz,M.M.; Webster,R.G.
EPI_ISL_144283	HA	Bangladesh	2011-Jun-28	A/environment/Bangladesh/11173/2011	NA	H9	Import from public-domain	Shanmuganatham,K.; Feeroz,M.; Jones-Engel,L.; Smith,G.J.D.; Fourment,M.; Walker,D.; McClenaghan,L.; Rabiul Alam,S.M.; Hasan,K.; Seiler,P.; Franks,J.; Danner,A.; Barman,S.; McKenzie,P.; Krauss,S.; Webby,R.J.; Webster,R.G.; Engel,L.J.; Alam,S.M.R.; Ferguson,A.
EPI_ISL_379728	HA	Bangladesh	2017-Dec-25	A/duck/Bangladesh/34286/2017	NA	H9	Import from public-domain	Barman,S.; Turner,J.C.; Hasan,M.K.; Akhtar,S.; Franks,J.; El-Shesheny,R.; Walker,D.; Seiler,P.; Friedman,K.; Kercher,L.; Kayali,G.; Jones-Engel,L.; McKenzie,P.; Krauss,S.; Webby,R.J.; Feeroz,M.M.; Webster,R.G.
EPI_ISL_144238	HA	Bangladesh	2011-Jun-30	A/chicken/Bangladesh/11315/2011	NA	H9	Import from public-domain	Shanmuganatham,K.; Feeroz,M.; Jones-Engel,L.; Smith,G.J.D.; Fourment,M.; Walker,D.; McClenaghan,L.; Rabiul Alam,S.M.; Hasan,K.; Seiler,P.; Franks,J.; Danner,A.; Barman,S.; McKenzie,P.; Krauss,S.; Webby,R.J.; Webster,R.G.; Engel,L.J.; Alam,S.M.R.; Ferguson,A.
EPI_ISL_144272	HA	Bangladesh	2010-Sep-01	A/chicken/Bangladesh/8725/2010	NA	H9	Import from public-domain	Shanmuganatham,K.; Feeroz,M.; Jones-Engel,L.; Smith,G.J.D.; Fourment,M.; Walker,D.; McClenaghan,L.; Rabiul Alam,S.M.; Hasan,K.; Seiler,P.; Franks,J.; Danner,A.; Barman,S.; McKenzie,P.; Krauss,S.; Webby,R.J.; Webster,R.G.; Engel,L.J.; Alam,S.M.R.; Ferguson,A.
EPI_ISL_161223	HA	Bangladesh	2013-Apr-29	A/quail/Bangladesh/19475/2013	NA	H9	Import from public-domain	Shanmuganatham,K.; Feeroz,M.; Jones Engel,L.; Walker,D.; McClenaghan,L.; Alam,R.S.; Hasan,K.; McKenzie,P.; Webby,R.J.; Webster,R.G.
EPI_ISL_503493	HA	Bangladesh	2019-Aug-19	A/chicken/Bangladesh/40619/2019	NA	H9	Import from public-domain	Barman,S.; Turner,J.C.; Hasan,M.; Akhtar,S.; Franks,J.; El-Shesheny,R.; Walker,D.; Seiler,P.; Mukherjee,N.; Kercher,L.; McKenzie,P.; Feeroz,M.; Webby,R.J.
EPI_ISL_144237	HA	Bangladesh	2010-Nov-15	A/environment/Bangladesh/9350/2010	NA	H9	Import from public-domain	Shanmuganatham,K.; Feeroz,M.; Jones-Engel,L.; Smith,G.J.D.; Fourment,M.; Walker,D.; McClenaghan,L.; Rabiul Alam,S.M.; Hasan,K.; Seiler,P.; Franks,J.; Danner,A.; Barman,S.; McKenzie,P.; Krauss,S.; Webby,R.J.; Webster,R.G.; Engel,L.J.; Alam,S.M.R.; Ferguson,A.
EPI_ISL_144269	HA	Bangladesh	2009-Dec-27	A/environment/Bangladesh/5144/2009	NA	H9	Import from public-domain	Shanmuganatham,K.; Feeroz,M.; Jones-Engel,L.; Smith,G.J.D.; Fourment,M.; Walker,D.; McClenaghan,L.; Rabiul Alam,S.M.; Hasan,K.; Seiler,P.; Franks,J.; Danner,A.; Barman,S.; McKenzie,P.; Krauss,S.; Webby,R.J.; Webster,R.G.; Engel,L.J.; Alam,S.M.R.; Ferguson,A.
EPI_ISL_284796	HA	Bangladesh	2016-May-26	A/chicken/Bangladesh/30030/2016	NA	H9	Import from public-domain	Barman,S.; Turner,J.C.; Hasan,M.K.; Akhtar,S.; Franks,J.; El-Shesheny,R.; Walker,D.; Seiler,P.; Friedman,K.; Kercher,L.; Kayali,G.; Jones-Engel,L.; McKenzie,P.; Krauss,S.; Webby,R.J.; Feeroz,M.M.; Webster,R.G.
EPI_ISL_388023	HA	Bangladesh	2018-Apr-26	A/quail/Bangladesh/35241/2018	NA	H9	Import from public-domain	Barman,S.; Turner,J.C.; Hasan,M.; Akhtar,S.; Franks,J.; El-Shesheny,R.; Walker,D.; Seiler,P.; Friedman,K.; Kercher,L.; McKenzie,P.; Webby,R.J.; Feeroz,M.; Webster,R.G.
EPI_ISL_388012	HA	Bangladesh	2018-Jun-05	A/quail/Bangladesh/35454/2018	NA	H9	Import from public-domain	Barman,S.; Turner,J.C.; Hasan,M.; Akhtar,S.; Franks,J.; El-Shesheny,R.; Walker,D.; Seiler,P.; Friedman,K.; Kercher,L.; McKenzie,P.; Webby,R.J.; Feeroz,M.; Webster,R.G.
EPI_ISL_144242	HA	Bangladesh	2011-Jun-28	A/chicken/Bangladesh/11154/2011	NA	H9	Import from public-domain	Shanmuganatham,K.; Feeroz,M.; Jones-Engel,L.; Smith,G.J.D.; Fourment,M.; Walker,D.; McClenaghan,L.; Rabiul Alam,S.M.; Hasan,K.; Seiler,P.; Franks,J.; Danner,A.; Barman,S.; McKenzie,P.; Krauss,S.; Webby,R.J.; Webster,R.G.; Engel,L.J.; Alam,S.M.R.; Ferguson,A.
EPI_ISL_379713	HA	Bangladesh	2018-Jan-23	A/environment/Bangladesh/34574/2018	NA	H9	Import from public-domain	Barman,S.; Turner,J.C.; Hasan,M.K.; Akhtar,S.; Franks,J.; El-Shesheny,R.; Walker,D.; Seiler,P.; Friedman,K.; Kercher,L.; Kayali,G.; Jones-Engel,L.; McKenzie,P.; Krauss,S.; Webby,R.J.; Feeroz,M.M.; Webster,R.G.
EPI_ISL_144241	HA	Bangladesh	2009-Jan-07	A/environment/Bangladesh/907/2009	NA	H9	Import from public-domain	Shanmuganatham,K.; Feeroz,M.; Jones-Engel,L.; Smith,G.J.D.; Fourment,M.; Walker,D.; McClenaghan,L.; Rabiul Alam,S.M.; Hasan,K.; Seiler,P.; Franks,J.; Danner,A.; Barman,S.; McKenzie,P.; Krauss,S.; Webby,R.J.; Webster,R.G.; Engel,L.J.; Alam,S.M.R.; Ferguson,A.
EPI_ISL_165816	HA	Bangladesh	2012-Dec-15	A/poultry/Bangladesh/91311/2012	Institute of Epidemiology Disease Control and Research (IEDCR) & Bangladesh National Influenza Centre (NIC)	H9	Centers for Disease Control and Prevention	Gerloff, Nancy; Simpson, Natosha; Davis, C. Todd

EPI_ISL_144274	HA	Bangladesh	2010-Sep-01	A/environment/Bangladesh/8465/2010	NA	H9	Import from public-domain	Shanmuganatham,K.; Feeroz,M.; Jones-Engel,L.; Smith,G.J.D.; Fourment,M.; Walker,D.; McClenaghan,L.; Rabiul Alam,S.M.; Hasan,K.; Seiler,P.; Franks,J.; Danner,A.; Barman,S.; McKenzie,P.; Krauss,S.; Webby,R.J.; Webster,R.G.; Engel,L.J.; Alam,S.M.R.; Ferguson,A.
EPI_ISL_284797	HA	Bangladesh	2016-Nov-25	A/quail/Bangladesh/31244/2016	NA	H9	Import from public-domain	Barman,S.; Turner,J.C.; Hasan,M.K.; Akhtar,S.; Franks,J.; El-Shesheny,R.; Walker,D.; Seiler,P.; Friedman,K.; Kercher,L.; Kayali,G.; Jones-Engel,L.; McKenzie,P.; Krauss,S.; Webby,R.J.; Feeroz,M.M.; Webster,R.G.
EPI_ISL_284791	HA	Bangladesh	2016-Oct-24	A/chicken/Bangladesh/31066/2016	NA	H9	Import from public-domain	Barman,S.; Turner,J.C.; Hasan,M.K.; Akhtar,S.; Franks,J.; El-Shesheny,R.; Walker,D.; Seiler,P.; Friedman,K.; Kercher,L.; Kayali,G.; Jones-Engel,L.; McKenzie,P.; Krauss,S.; Webby,R.J.; Feeroz,M.M.; Webster,R.G.
EPI_ISL_284800	HA	Bangladesh	2016-Mar-28	A/quail/Bangladesh/29564/2016	NA	H9	Import from public-domain	Barman,S.; Turner,J.C.; Hasan,M.K.; Akhtar,S.; Franks,J.; El-Shesheny,R.; Walker,D.; Seiler,P.; Friedman,K.; Kercher,L.; Kayali,G.; Jones-Engel,L.; McKenzie,P.; Krauss,S.; Webby,R.J.; Feeroz,M.M.; Webster,R.G.
EPI_ISL_329574	HA	Bangladesh	2017-Jul-21	A/chicken/Bangladesh/33113/2017	NA	H9	Import from public-domain	Barman,S.; Turner,J.C.; Hasan,M.K.; Akhtar,S.; Franks,J.; El-Shesheny,R.; Walker,D.; Seiler,P.; Friedman,K.; Kercher,L.; Kayali,G.; Jones-Engel,L.; McKenzie,P.; Krauss,S.; Webby,R.J.; Feeroz,M.; Webster,R.G.
EPI_ISL_144285	HA	Bangladesh	2011-Feb-19	A/environment/Bangladesh/10313/2011	NA	H9	Import from public-domain	Shanmuganatham,K.; Feeroz,M.; Jones-Engel,L.; Smith,G.J.D.; Fourment,M.; Walker,D.; McClenaghan,L.; Rabiul Alam,S.M.; Hasan,K.; Seiler,P.; Franks,J.; Danner,A.; Barman,S.; McKenzie,P.; Krauss,S.; Webby,R.J.; Webster,R.G.; Engel,L.J.; Alam,S.M.R.; Ferguson,A.
EPI_ISL_329583	HA	Bangladesh	2017-Jul-21	A/duck/Bangladesh/33138/2017	NA	H9	Import from public-domain	Barman,S.; Turner,J.C.; Hasan,M.K.; Akhtar,S.; Franks,J.; El-Shesheny,R.; Walker,D.; Seiler,P.; Friedman,K.; Kercher,L.; Kayali,G.; Jones-Engel,L.; McKenzie,P.; Krauss,S.; Webby,R.J.; Feeroz,M.; Webster,R.G.
EPI_ISL_284772	HA	Bangladesh	2016-Sep-27	A/chicken/Bangladesh/30861/2016	NA	H9	Import from public-domain	Barman,S.; Turner,J.C.; Hasan,M.K.; Akhtar,S.; Franks,J.; El-Shesheny,R.; Walker,D.; Seiler,P.; Friedman,K.; Kercher,L.; Kayali,G.; Jones-Engel,L.; McKenzie,P.; Krauss,S.; Webby,R.J.; Feeroz,M.M.; Webster,R.G.
EPI_ISL_387978	HA	Bangladesh	2018-Dec-18	A/chicken/Bangladesh/35970/2018	NA	H9	Import from public-domain	Barman,S.; Turner,J.C.; Hasan,M.; Akhtar,S.; Franks,J.; El-Shesheny,R.; Walker,D.; Seiler,P.; Friedman,K.; Kercher,L.; McKenzie,P.; Webby,R.J.; Feeroz,M.; Webster,R.G.
EPI_ISL_379724	HA	Bangladesh	2017-Nov-28	A/chicken/Bangladesh/34004/2017	NA	H9	Import from public-domain	Barman,S.; Turner,J.C.; Hasan,M.K.; Akhtar,S.; Franks,J.; El-Shesheny,R.; Walker,D.; Seiler,P.; Friedman,K.; Kercher,L.; Kayali,G.; Jones-Engel,L.; McKenzie,P.; Krauss,S.; Webby,R.J.; Feeroz,M.M.; Webster,R.G.
EPI_ISL_27602	HA	India	2004-Jan-01	A/chicken/Uttar Pradesh/2543/2004	NA	H9	Import from public-domain	Nagarajan,S.; Rajukumar,K.; Ramaswamy,V.; Purohit,K.; Pattnaik,B.; Pradhan,H.K.
EPI_ISL_388031	HA	Bangladesh	2018-Jun-05	A/chicken/Bangladesh/35410/2018	NA	H9	Import from public-domain	Barman,S.; Turner,J.C.; Hasan,M.; Akhtar,S.; Franks,J.; El-Shesheny,R.; Walker,D.; Seiler,P.; Friedman,K.; Kercher,L.; McKenzie,P.; Webby,R.J.; Feeroz,M.; Webster,R.G.
EPI_ISL_161203	HA	Bangladesh	2012-Oct-21	A/quail/Bangladesh/18210/2012	NA	H9	Import from public-domain	Shanmuganatham,K.; Feeroz,M.; Jones Engel,L.; Walker,D.; McClenaghan,L.; Alam,R.S.; Hasan,K.; McKenzie,P.; Webby,R.J.; Webster,R.G.
EPI_ISL_78705	HA	India	2004-Apr-12	A/chicken/Haryana/2424/2004	NA	H9	Import from public-domain	Tosh,C.; Nagarajan,S.; Behera,P.; Pradhan,H.K.; Dubey,S.C.
EPI_ISL_161222	HA	Bangladesh	2013-Apr-29	A/chicken/Bangladesh/19565/2013	NA	H9	Import from public-domain	Shanmuganatham,K.; Feeroz,M.; Jones Engel,L.; Walker,D.; McClenaghan,L.; Alam,R.S.; Hasan,K.; McKenzie,P.; Webby,R.J.; Webster,R.G.
EPI_ISL_161217	HA	Bangladesh	2013-Apr-29	A/chicken/Bangladesh/19495/2013	NA	H9	Import from public-domain	Shanmuganatham,K.; Feeroz,M.; Jones Engel,L.; Walker,D.; McClenaghan,L.; Alam,R.S.; Hasan,K.; McKenzie,P.; Webby,R.J.; Webster,R.G.
EPI_ISL_329561	HA	Bangladesh	2017-Sep-28	A/chicken/Bangladesh/33649/2017	NA	H9	Import from public-domain	Barman,S.; Turner,J.C.; Hasan,M.K.; Akhtar,S.; Franks,J.; El-Shesheny,R.; Walker,D.; Seiler,P.; Friedman,K.; Kercher,L.; Kayali,G.; Jones-Engel,L.; McKenzie,P.; Krauss,S.; Webby,R.J.; Feeroz,M.; Webster,R.G.
EPI_ISL_76224	HA	India	2006-Apr-25	A/chicken/Uchal/8293/2006	NA	H9	Import from public-domain	Maheswarappa,G.; Byregowda,S.M.; Pradhan,H.K.; Tosh,C.; Dubey,S.C.
EPI_ISL_379707	HA	Bangladesh	2018-Jan-23	A/environment/Bangladesh/34578/2018	NA	H9	Import from public-domain	Barman,S.; Turner,J.C.; Hasan,M.K.; Akhtar,S.; Franks,J.; El-Shesheny,R.; Walker,D.; Seiler,P.; Friedman,K.; Kercher,L.; Kayali,G.; Jones-Engel,L.; McKenzie,P.; Krauss,S.; Webby,R.J.; Feeroz,M.M.; Webster,R.G.
EPI_ISL_161219	HA	Bangladesh	2013-Apr-29	A/quail/Bangladesh/19462/2013	NA	H9	Import from public-domain	Shanmuganatham,K.; Feeroz,M.; Jones Engel,L.; Walker,D.; McClenaghan,L.; Alam,R.S.; Hasan,K.; McKenzie,P.; Webby,R.J.; Webster,R.G.
EPI_ISL_388022	HA	Bangladesh	2018-Jun-05	A/chicken/Bangladesh/35407/2018	NA	H9	Import from public-domain	Barman,S.; Turner,J.C.; Hasan,M.; Akhtar,S.; Franks,J.; El-Shesheny,R.; Walker,D.; Seiler,P.; Friedman,K.; Kercher,L.; McKenzie,P.; Webby,R.J.; Feeroz,M.; Webster,R.G.
EPI_ISL_387983	HA	Bangladesh	2018-Dec-18	A/quail/Bangladesh/35929/2018	NA	H9	Import from public-domain	Barman,S.; Turner,J.C.; Hasan,M.; Akhtar,S.; Franks,J.; El-Shesheny,R.; Walker,D.; Seiler,P.; Friedman,K.; Kercher,L.; McKenzie,P.; Webby,R.J.; Feeroz,M.; Webster,R.G.
EPI_ISL_161221	HA	Bangladesh	2012-Nov-15	A/chicken/Bangladesh/18460/2012	NA	H9	Import from public-domain	Shanmuganatham,K.; Feeroz,M.; Jones Engel,L.; Walker,D.; McClenaghan,L.; Alam,R.S.; Hasan,K.; McKenzie,P.; Webby,R.J.; Webster,R.G.
EPI_ISL_144268	HA	Bangladesh	2009-Aug-10	A/pigeon/Bangladesh/4303/2009	NA	H9	Import from public-domain	Shanmuganatham,K.; Feeroz,M.; Jones-Engel,L.; Smith,G.J.D.; Fourment,M.; Walker,D.; McClenaghan,L.; Rabiul Alam,S.M.; Hasan,K.; Seiler,P.; Franks,J.; Danner,A.; Barman,S.; McKenzie,P.; Krauss,S.; Webby,R.J.; Webster,R.G.; Engel,L.J.; Alam,S.M.R.; Ferguson,A.
EPI_ISL_503504	HA	Bangladesh	2019-Oct-16	A/chicken/Bangladesh/40960/2019	NA	H9	Import from public-domain	Barman,S.; Turner,J.C.; Hasan,M.; Akhtar,S.; Franks,J.; El-Shesheny,R.; Walker,D.; Seiler,P.; Mukherjee,N.; Kercher,L.; McKenzie,P.; Feeroz,M.; Webby,R.J.
EPI_ISL_144271	HA	Bangladesh	2011-May-29	A/chicken/Bangladesh/10897/2011	NA	H9	Import from public-domain	Shanmuganatham,K.; Feeroz,M.; Jones-Engel,L.; Smith,G.J.D.; Fourment,M.; Walker,D.; McClenaghan,L.; Rabiul Alam,S.M.; Hasan,K.; Seiler,P.; Franks,J.; Danner,A.; Barman,S.; McKenzie,P.; Krauss,S.; Webby,R.J.; Webster,R.G.; Engel,L.J.; Alam,S.M.R.; Ferguson,A.
EPI_ISL_257045	HA	Bangladesh	2015-Aug-27	A/chicken/Bangladesh/26115/2015	NA	H9	Import from public-domain	Barman,S.; Marinova-Petkova,A.; Hasan,M.K.; Akhtar,S.; Turner,J.C.; Franks,J.; Walker,D.; Seiler,P.; Friedman,K.; Kercher,L.; Kayali,G.; Jones-Engel,L.; McKenzie,P.; Krauss,S.; Webby,R.J.; Feeroz,M.M.; Webster,R.G.
EPI_ISL_256790	HA	Bangladesh	2014-Oct-18	A/environment/Bangladesh/23641/2014	NA	H9	Import from public-domain	Shanmuganatham,K.; Barman,S.; Marinova-Petkova,A.; Hasan,M.K.; Akhtar,S.; Turner,J.C.; Franks,J.; Walker,D.; Seiler,P.; Friedman,K.; Jones-Engel,L.; McKenzie,P.; Krauss,S.; Webby,R.J.; Feeroz,M.M.; Webster,R.G.
EPI_ISL_329576	HA	Bangladesh	2017-Aug-17	A/chicken/Bangladesh/33386/2017	NA	H9	Import from public-domain	Barman,S.; Turner,J.C.; Hasan,M.K.; Akhtar,S.; Franks,J.; El-Shesheny,R.; Walker,D.; Seiler,P.; Friedman,K.; Kercher,L.; Kayali,G.; Jones-Engel,L.; McKenzie,P.; Krauss,S.; Webby,R.J.; Feeroz,M.; Webster,R.G.
EPI_ISL_161230	HA	Bangladesh	2013-Jan-28	A/chicken/Bangladesh/18857/2013	NA	H9	Import from public-domain	Shanmuganatham,K.; Feeroz,M.; Jones Engel,L.; Walker,D.; McClenaghan,L.; Alam,R.S.; Hasan,K.; McKenzie,P.; Webby,R.J.; Webster,R.G.
EPI_ISL_161199	HA	Bangladesh	2012-Nov-20	A/chicken/Bangladesh/18549/2012	NA	H9	Import from public-domain	Shanmuganatham,K.; Feeroz,M.; Jones Engel,L.; Walker,D.; McClenaghan,L.; Alam,R.S.; Hasan,K.; McKenzie,P.; Webby,R.J.; Webster,R.G.
EPI_ISL_284809	HA	Bangladesh	2016-Oct-24	A/quail/Bangladesh/31043/2016	NA	H9	Import from public-domain	Barman,S.; Turner,J.C.; Hasan,M.K.; Akhtar,S.; Franks,J.; El-Shesheny,R.; Walker,D.; Seiler,P.; Friedman,K.; Kercher,L.; Kayali,G.; Jones-Engel,L.; McKenzie,P.; Krauss,S.; Webby,R.J.; Feeroz,M.M.; Webster,R.G.
EPI_ISL_161220	HA	Bangladesh	2013-Mar-30	A/environment/Bangladesh/19301/2013	NA	H9	Import from public-domain	Shanmuganatham,K.; Feeroz,M.; Jones Engel,L.; Walker,D.; McClenaghan,L.; Alam,R.S.; Hasan,K.; McKenzie,P.; Webby,R.J.; Webster,R.G.
EPI_ISL_144259	HA	Bangladesh	2010-Dec-09	A/environment/Bangladesh/9457/2010	NA	H9	Import from public-domain	Shanmuganatham,K.; Feeroz,M.; Jones-Engel,L.; Smith,G.J.D.; Fourment,M.; Walker,D.; McClenaghan,L.; Rabiul Alam,S.M.; Hasan,K.; Seiler,P.; Franks,J.; Danner,A.; Barman,S.; McKenzie,P.; Krauss,S.; Webby,R.J.; Webster,R.G.; Engel,L.J.; Alam,S.M.R.; Ferguson,A.
EPI_ISL_503512	HA	Bangladesh	2019-Jul-11	A/quail/Bangladesh/40420/2019	NA	H9	Import from public-domain	Barman,S.; Turner,J.C.; Hasan,M.; Akhtar,S.; Franks,J.; El-Shesheny,R.; Walker,D.; Seiler,P.; Mukherjee,N.; Kercher,L.; McKenzie,P.; Feeroz,M.; Webby,R.J.
EPI_ISL_161688	HA	Bangladesh	2013-Oct-25	A/duck/Bangladesh/21126/2013	NA	H9	Import from public-domain	Shanmuganatham,K.; Feeroz,M.; Jones Engel,L.; Walker,D.; McClenaghan,L.; Alam,R.S.; Hasan,K.; McKenzie,P.; Webby,R.J.; Webster,R.G.
EPI_ISL_387991	HA	Bangladesh	2018-Aug-09	A/chicken/Bangladesh/35646/2018	NA	H9	Import from public-domain	Barman,S.; Turner,J.C.; Hasan,M.; Akhtar,S.; Franks,J.; El-Shesheny,R.; Walker,D.; Seiler,P.; Friedman,K.; Kercher,L.; McKenzie,P.; Webby,R.J.; Feeroz,M.; Webster,R.G.
EPI_ISL_503517	HA	Bangladesh	2019-Aug-19	A/chicken/Bangladesh/40618/2019	NA	H9	Import from public-domain	Barman,S.; Turner,J.C.; Hasan,M.; Akhtar,S.; Franks,J.; El-Shesheny,R.; Walker,D.; Seiler,P.; Mukherjee,N.; Kercher,L.; McKenzie,P.; Feeroz,M.; Webby,R.J.
EPI_ISL_144267	HA	Bangladesh	2011-Feb-19	A/environment/Bangladesh/10306/2011	NA	H9	Import from public-domain	Shanmuganatham,K.; Feeroz,M.; Jones-Engel,L.; Smith,G.J.D.; Fourment,M.; Walker,D.; McClenaghan,L.; Rabiul Alam,S.M.; Hasan,K.; Seiler,P.; Franks,J.; Danner,A.; Barman,S.; McKenzie,P.; Krauss,S.; Webby,R.J.; Webster,R.G.; Engel,L.J.; Alam,S.M.R.; Ferguson,A.
EPI_ISL_329567	HA	Bangladesh	2017-Jul-21	A/chicken/Bangladesh/33111/2017	NA	H9	Import from public-domain	Barman,S.; Turner,J.C.; Hasan,M.K.; Akhtar,S.; Franks,J.; El-Shesheny,R.; Walker,D.; Seiler,P.; Friedman,K.; Kercher,L.; Kayali,G.; Jones-Engel,L.; McKenzie,P.; Krauss,S.; Webby,R.J.; Feeroz,M.; Webster,R.G.
EPI_ISL_379705	HA	Bangladesh	2017-Nov-28	A/environment/Bangladesh/34092/2017	NA	H9	Import from public-domain	Barman,S.; Turner,J.C.; Hasan,M.K.; Akhtar,S.; Franks,J.; El-Shesheny,R.; Walker,D.; Seiler,P.; Friedman,K.; Kercher,L.; Kayali,G.; Jones-Engel,L.; McKenzie,P.; Krauss,S.; Webby,R.J.; Feeroz,M.M.; Webster,R.G.
EPI_ISL_284811	HA	Bangladesh	2016-May-26	A/quail/Bangladesh/29997/2016	NA	H9	Import from public-domain	Barman,S.; Turner,J.C.; Hasan,M.K.; Akhtar,S.; Franks,J.; El-Shesheny,R.; Walker,D.; Seiler,P.; Friedman,K.; Kercher,L.; Kayali,G.; Jones-Engel,L.; McKenzie,P.; Krauss,S.; Webby,R.J.; Feeroz,M.M.; Webster,R.G.
EPI_ISL_379687	HA	Bangladesh	2017-Dec-25	A/chicken/Bangladesh/34321/2017	NA	H9	Import from public-domain	Barman,S.; Turner,J.C.; Hasan,M.K.; Akhtar,S.; Franks,J.; El-Shesheny,R.; Walker,D.; Seiler,P.; Friedman,K.; Kercher,L.; Kayali,G.; Jones-Engel,L.; McKenzie,P.; Krauss,S.; Webby,R.J.; Feeroz,M.M.; Webster,R.G.
EPI_ISL_161229	HA	Bangladesh	2012-Nov-25	A/environment/Bangladesh/18530/2012	NA	H9	Import from public-domain	Shanmuganatham,K.; Feeroz,M.; Jones Engel,L.; Walker,D.; McClenaghan,L.; Alam,R.S.; Hasan,K.; McKenzie,P.; Webby,R.J.; Webster,R.G.

EPI_ISL_379689	HA	Bangladesh	2017-Dec-25	A/environment/Bangladesh/34343/2017	NA	H9	Import from public-domain	Barman,S.; Turner,J.C.; Hasan,M.K.; Akhtar,S.; Franks,J.; El-Shesheny,R.; Walker,D.; Seiler,P.; Friedman,K.; Kercher,L.; Kayali,G.; Jones-Engel,L.; McKenzie,P.; Krauss,S.; Webby,R.J.; Feeroz,M.M.; Webster,R.G.
EPI_ISL_388026	HA	Bangladesh	2018-Apr-28	A/chicken/Bangladesh/35368/2018	NA	H9	Import from public-domain	Barman,S.; Turner,J.C.; Hasan,M.; Akhtar,S.; Franks,J.; El-Shesheny,R.; Walker,D.; Seiler,P.; Friedman,K.; Kercher,L.; McKenzie,P.; Webby,R.J.; Feeroz,M.; Webster,R.G.
EPI_ISL_379694	HA	Bangladesh	2017-Nov-28	A/chicken/Bangladesh/34075/2017	NA	H9	Import from public-domain	Barman,S.; Turner,J.C.; Hasan,M.K.; Akhtar,S.; Franks,J.; El-Shesheny,R.; Walker,D.; Seiler,P.; Friedman,K.; Kercher,L.; Kayali,G.; Jones-Engel,L.; McKenzie,P.; Krauss,S.; Webby,R.J.; Feeroz,M.M.; Webster,R.G.
EPI_ISL_161224	HA	Bangladesh	2012-Oct-21	A/chicken/Bangladesh/18301/2012	NA	H9	Import from public-domain	Shanmuganatham,K.; Feeroz,M.; Jones Engel,L.; Walker,D.; McClenaghan,L.; Alam,R.S.; Hasan,K.; McKenzie,P.; Webby,R.J.; Webster,R.G.
EPI_ISL_144276	HA	Bangladesh	2010-Aug-06	A/chicken/Bangladesh/8415/2010	NA	H9	Import from public-domain	Shanmuganatham,K.; Feeroz,M.; Jones-Engel,L.; Smith,G.J.D.; Fourment,M.; Walker,D.; McClenaghan,L.; Rabiul Alam,S.M.; Hasan,K.; Seiler,P.; Franks,J.; Danner,A.; Barman,S.; McKenzie,P.; Krauss,S.; Webby,R.J.; Webster,R.G.; Engel,L.J.; Alam,S.M.R.; Ferguson,A.
EPI_ISL_144236	HA	Bangladesh	2010-Nov-15	A/chicken/Bangladesh/9334/2010	NA	H9	Import from public-domain	Shanmuganatham,K.; Feeroz,M.; Jones-Engel,L.; Smith,G.J.D.; Fourment,M.; Walker,D.; McClenaghan,L.; Rabiul Alam,S.M.; Hasan,K.; Seiler,P.; Franks,J.; Danner,A.; Barman,S.; McKenzie,P.; Krauss,S.; Webby,R.J.; Webster,R.G.; Engel,L.J.; Alam,S.M.R.; Ferguson,A.
EPI_ISL_329572	HA	Bangladesh	2017-Aug-17	A/chicken/Bangladesh/33338/2017	NA	H9	Import from public-domain	Barman,S.; Turner,J.C.; Hasan,M.K.; Akhtar,S.; Franks,J.; El-Shesheny,R.; Walker,D.; Seiler,P.; Friedman,K.; Kercher,L.; Kayali,G.; Jones-Engel,L.; McKenzie,P.; Krauss,S.; Webby,R.J.; Feeroz,M.; Webster,R.G.
EPI_ISL_144228	HA	Bangladesh	2009-Feb-03	A/duck/Bangladesh/1231/2009	NA	H9	Import from public-domain	Shanmuganatham,K.; Feeroz,M.; Jones-Engel,L.; Smith,G.J.D.; Fourment,M.; Walker,D.; McClenaghan,L.; Rabiul Alam,S.M.; Hasan,K.; Seiler,P.; Franks,J.; Danner,A.; Barman,S.; McKenzie,P.; Krauss,S.; Webby,R.J.; Webster,R.G.; Engel,L.J.; Alam,S.M.R.; Ferguson,A.
EPI_ISL_306668	HA	Bangladesh	2017-Feb-15	A/quail/Bangladesh/32020/2017	NA	H9	Import from public-domain	Barman,S.; Turner,J.C.; Hasan,M.K.; Akhtar,S.; Franks,J.; El-Shesheny,R.; Walker,D.; Seiler,P.; Friedman,K.; Kercher,L.; Kayali,G.; Jones-Engel,L.; McKenzie,P.; Krauss,S.; Webby,R.J.; Feeroz,M.M.; Webster,R.G.
EPI_ISL_161233	HA	Bangladesh	2012-Oct-21	A/environment/Bangladesh/18311/2012	NA	H9	Import from public-domain	Shanmuganatham,K.; Feeroz,M.; Jones Engel,L.; Walker,D.; McClenaghan,L.; Alam,R.S.; Hasan,K.; McKenzie,P.; Webby,R.J.; Webster,R.G.
EPI_ISL_257094	HA	Bangladesh	2015-Jan-23	A/environment/Bangladesh/24205/2015	NA	H9	Import from public-domain	Shanmuganatham,K.; Barman,S.; Marinova-Petkova,A.; Hasan,M.K.; Akhtar,S.; Turner,J.C.; Franks,J.; Walker,D.; Seiler,P.; Friedman,K.; Jones-Engel,L.; McKenzie,P.; Krauss,S.; Webby,R.J.; Feeroz,M.M.; Webster,R.G.
EPI_ISL_388010	HA	Bangladesh	2018-Oct-30	A/chicken/Bangladesh/35732/2018	NA	H9	Import from public-domain	Barman,S.; Turner,J.C.; Hasan,M.; Akhtar,S.; Franks,J.; El-Shesheny,R.; Walker,D.; Seiler,P.; Friedman,K.; Kercher,L.; McKenzie,P.; Webby,R.J.; Feeroz,M.; Webster,R.G.
EPI_ISL_329580	HA	Bangladesh	2017-Aug-17	A/duck/Bangladesh/33351/2017	NA	H9	Import from public-domain	Barman,S.; Turner,J.C.; Hasan,M.K.; Akhtar,S.; Franks,J.; El-Shesheny,R.; Walker,D.; Seiler,P.; Friedman,K.; Kercher,L.; Kayali,G.; Jones-Engel,L.; McKenzie,P.; Krauss,S.; Webby,R.J.; Feeroz,M.; Webster,R.G.
EPI_ISL_284810	HA	Bangladesh	2016-Sep-27	A/quail/Bangladesh/30835/2016	NA	H9	Import from public-domain	Barman,S.; Turner,J.C.; Hasan,M.K.; Akhtar,S.; Franks,J.; El-Shesheny,R.; Walker,D.; Seiler,P.; Friedman,K.; Kercher,L.; Kayali,G.; Jones-Engel,L.; McKenzie,P.; Krauss,S.; Webby,R.J.; Feeroz,M.M.; Webster,R.G.
EPI_ISL_144234	HA	Bangladesh	2010-Aug-06	A/chicken/Bangladesh/8411/2010	NA	H9	Import from public-domain	Shanmuganatham,K.; Feeroz,M.; Jones-Engel,L.; Smith,G.J.D.; Fourment,M.; Walker,D.; McClenaghan,L.; Rabiul Alam,S.M.; Hasan,K.; Seiler,P.; Franks,J.; Danner,A.; Barman,S.; McKenzie,P.; Krauss,S.; Webby,R.J.; Webster,R.G.; Engel,L.J.; Alam,S.M.R.; Ferguson,A.
EPI_ISL_257062	HA	Bangladesh	2016-Feb-05	A/chicken/Bangladesh/28182/2016	NA	H9	Import from public-domain	Barman,S.; Marinova-Petkova,A.; Hasan,M.K.; Akhtar,S.; Turner,J.C.; Franks,J.; Walker,D.; Seiler,P.; Friedman,K.; Kercher,L.; Kayali,G.; Jones-Engel,L.; McKenzie,P.; Krauss,S.; Webby,R.J.; Feeroz,M.M.; Webster,R.G.
EPI_ISL_76225	HA	India	2006-Apr-25	A/chicken/Uchal/8286/2006	NA	H9	Import from public-domain	Maheswarappa,G.; Byregowda,S.M.; Pradhan,H.K.; Tosh,C.; Dubey,S.C.
EPI_ISL_165819	HA	Bangladesh	2012-Nov-20	A/avian/Bangladesh/91277/2012	Institute of Epidemiology Disease Control and Research (IEDCR) & Bangladesh National Influenza Centre (NIC)	H9	Centers for Disease Control and Prevention	Gerloff, Nancy; Simpson, Natosha; Davis, C. Todd
EPI_ISL_161236	HA	Bangladesh	2012-Oct-21	A/chicken/Bangladesh/18276/2012	NA	H9	Import from public-domain	Shanmuganatham,K.; Feeroz,M.; Jones Engel,L.; Walker,D.; McClenaghan,L.; Alam,R.S.; Hasan,K.; McKenzie,P.; Webby,R.J.; Webster,R.G.
EPI_ISL_257083	HA	Bangladesh	2015-Aug-25	A/quail/Bangladesh/25987/2015	NA	H9	Import from public-domain	Barman,S.; Marinova-Petkova,A.; Hasan,M.K.; Akhtar,S.; Turner,J.C.; Franks,J.; Walker,D.; Seiler,P.; Friedman,K.; Kercher,L.; Kayali,G.; Jones-Engel,L.; McKenzie,P.; Krauss,S.; Webby,R.J.; Feeroz,M.M.; Webster,R.G.
EPI_ISL_161232	HA	Bangladesh	2012-Mar-22	A/chicken/Bangladesh/16448/2012	NA	H9	Import from public-domain	Shanmuganatham,K.; Feeroz,M.; Jones Engel,L.; Walker,D.; McClenaghan,L.; Alam,R.S.; Hasan,K.; McKenzie,P.; Webby,R.J.; Webster,R.G.
EPI_ISL_388032	HA	Bangladesh	2018-Jun-05	A/chicken/Bangladesh/35417/2018	NA	H9	Import from public-domain	Barman,S.; Turner,J.C.; Hasan,M.; Akhtar,S.; Franks,J.; El-Shesheny,R.; Walker,D.; Seiler,P.; Friedman,K.; Kercher,L.; McKenzie,P.; Webby,R.J.; Feeroz,M.; Webster,R.G.
EPI_ISL_27603	HA	India	2004-Jan-01	A/chicken/Gujarat/3724/2004		H9	Import from public-domain	
EPI_ISL_165813	HA	Bangladesh	2010-Jul-19	A/environment/Bangladesh/124/2010	Institute of Epidemiology Disease Control and Research (IEDCR) & Bangladesh National Influenza Centre (NIC)	H9	Centers for Disease Control and Prevention	Gerloff, Nancy; Simpson, Natosha; Davis, C. Todd
EPI_ISL_329569	HA	Bangladesh	2017-Sep-28	A/chicken/Bangladesh/33648/2017	NA	H9	Import from public-domain	Barman,S.; Turner,J.C.; Hasan,M.K.; Akhtar,S.; Franks,J.; El-Shesheny,R.; Walker,D.; Seiler,P.; Friedman,K.; Kercher,L.; Kayali,G.; Jones-Engel,L.; McKenzie,P.; Krauss,S.; Webby,R.J.; Feeroz,M.; Webster,R.G.
EPI_ISL_161198	HA	Bangladesh	2013-Mar-30	A/environment/Bangladesh/19336/2013	NA	H9	Import from public-domain	Shanmuganatham,K.; Feeroz,M.; Jones Engel,L.; Walker,D.; McClenaghan,L.; Alam,R.S.; Hasan,K.; McKenzie,P.; Webby,R.J.; Webster,R.G.
EPI_ISL_165821	HA	Bangladesh	2012-Nov-29	A/avian/Bangladesh/91286/2012	Institute of Epidemiology Disease Control and Research (IEDCR) & Bangladesh National Influenza Centre (NIC)	H9	Centers for Disease Control and Prevention	Gerloff, Nancy; Simpson, Natosha; Davis, C. Todd
EPI_ISL_144270	HA	Bangladesh	2009-Dec-27	A/chicken/Bangladesh/5209/2009	NA	H9	Import from public-domain	Shanmuganatham,K.; Feeroz,M.; Jones-Engel,L.; Smith,G.J.D.; Fourment,M.; Walker,D.; McClenaghan,L.; Rabiul Alam,S.M.; Hasan,K.; Seiler,P.; Franks,J.; Danner,A.; Barman,S.; McKenzie,P.; Krauss,S.; Webby,R.J.; Webster,R.G.; Engel,L.J.; Alam,S.M.R.; Ferguson,A.
EPI_ISL_144280	HA	Bangladesh	2011-Jul-20	A/environment/Bangladesh/11597/2011	NA	H9	Import from public-domain	Shanmuganatham,K.; Feeroz,M.; Jones-Engel,L.; Smith,G.J.D.; Fourment,M.; Walker,D.; McClenaghan,L.; Rabiul Alam,S.M.; Hasan,K.; Seiler,P.; Franks,J.; Danner,A.; Barman,S.; McKenzie,P.; Krauss,S.; Webby,R.J.; Webster,R.G.; Engel,L.J.; Alam,S.M.R.; Ferguson,A.
EPI_ISL_144282	HA	Bangladesh	2011-Feb-19	A/environment/Bangladesh/10307/2011	NA	H9	Import from public-domain	Shanmuganatham,K.; Feeroz,M.; Jones-Engel,L.; Smith,G.J.D.; Fourment,M.; Walker,D.; McClenaghan,L.; Rabiul Alam,S.M.; Hasan,K.; Seiler,P.; Franks,J.; Danner,A.; Barman,S.; McKenzie,P.; Krauss,S.; Webby,R.J.; Webster,R.G.; Engel,L.J.; Alam,S.M.R.; Ferguson,A.
EPI_ISL_78706	HA	India	2004-Feb-23	A/chicken/Orissa/2317/2004	NA	H9	Import from public-domain	Tosh,C.; Nagarajan,S.; Behera,P.; Pradhan,H.K.; Dubey,S.C.
EPI_ISL_284711	HA	Bangladesh	2016-Jul-22	A/chicken/Bangladesh/30461/2016	NA	H9	Import from public-domain	Barman,S.; Turner,J.C.; Hasan,M.K.; Akhtar,S.; Franks,J.; El-Shesheny,R.; Walker,D.; Seiler,P.; Friedman,K.; Kercher,L.; Kayali,G.; Jones-Engel,L.; McKenzie,P.; Krauss,S.; Webby,R.J.; Feeroz,M.M.; Webster,R.G.
EPI_ISL_161684	HA	Bangladesh	2013-Dec-20	A/chicken/Bangladesh/21554/2013	NA	H9	Import from public-domain	Shanmuganatham,K.; Feeroz,M.; Jones Engel,L.; Walker,D.; McClenaghan,L.; Alam,R.S.; Hasan,K.; McKenzie,P.; Webby,R.J.; Webster,R.G.
EPI_ISL_379706	HA	Bangladesh	2017-Nov-28	A/environment/Bangladesh/34095/2017	NA	H9	Import from public-domain	Barman,S.; Turner,J.C.; Hasan,M.K.; Akhtar,S.; Franks,J.; El-Shesheny,R.; Walker,D.; Seiler,P.; Friedman,K.; Kercher,L.; Kayali,G.; Jones-Engel,L.; McKenzie,P.; Krauss,S.; Webby,R.J.; Feeroz,M.M.; Webster,R.G.
EPI_ISL_397432	HA	Bangladesh	2017-Aug-17	A/quail/Bangladesh/33376/2017	NA	H9	Import from public-domain	Barman,S.; Turner,J.C.; Hasan,M.K.; Akhtar,S.; Franks,J.; El-Shesheny,R.; Walker,D.; Seiler,P.; Friedman,K.; Kercher,L.; Kayali,G.; Jones-Engel,L.; McKenzie,P.; Krauss,S.; Webby,R.J.; Feeroz,M.; Webster,R.G.
EPI_ISL_257099	HA	Bangladesh	2015-Aug-25	A/chicken/Bangladesh/25946/2015	NA	H9	Import from public-domain	Barman,S.; Marinova-Petkova,A.; Hasan,M.K.; Akhtar,S.; Turner,J.C.; Franks,J.; Walker,D.; Seiler,P.; Friedman,K.; Kercher,L.; Kayali,G.; Jones-Engel,L.; McKenzie,P.; Krauss,S.; Webby,R.J.; Feeroz,M.M.; Webster,R.G.
EPI_ISL_144273	HA	Bangladesh	2010-Aug-07	A/environment/Bangladesh/8202/2010	NA	H9	Import from public-domain	Shanmuganatham,K.; Feeroz,M.; Jones-Engel,L.; Smith,G.J.D.; Fourment,M.; Walker,D.; McClenaghan,L.; Rabiul Alam,S.M.; Hasan,K.; Seiler,P.; Franks,J.; Danner,A.; Barman,S.; McKenzie,P.; Krauss,S.; Webby,R.J.; Webster,R.G.; Engel,L.J.; Alam,S.M.R.; Ferguson,A.

EPI_ISL_144233	HA	Bangladesh	2011-Aug-14	A/environment/Bangladesh/12077/2011	NA	H9	Import from public-domain	Shanmuganatham,K.; Feeroz,M.; Jones-Engel,L.; Smith,G.J.D.; Fourment,M.; Walker,D.; McClenaghan,L.; Rabiul Alam,S.M.; Hasan,K.; Seiler,P.; Franks,J.; Danner,A.; Barman,S.; McKenzie,P.; Krauss,S.; Webby,R.J.; Webster,R.G.; Engel,L.J.; Alam,S.M.R.; Ferguson,A.
EPI_ISL_161685	HA	Bangladesh	2013-Jul-08	A/chicken/Bangladesh/20205/2013	NA	H9	Import from public-domain	Shanmuganatham,K.; Feeroz,M.; Jones Engel,L.; Walker,D.; McClenaghan,L.; Alam,R.S.; Hasan,K.; McKenzie,P.; Webby,R.J.; Webster,R.G.
EPI_ISL_165815	HA	Bangladesh	2010-Dec-12	A/environment/Bangladesh/177/2010	Institute of Epidemiology Disease Control and Research (IEDCR) & Bangladesh National Influenza Centre (NIC)	H9	Centers for Disease Control and Prevention	Gerloff, Nancy; Simpson, Natosha; Davis, C. Todd
EPI_ISL_161225	HA	Bangladesh	2013-May-25	A/environment/Bangladesh/19842/2013	NA	H9	Import from public-domain	Shanmuganatham,K.; Feeroz,M.; Jones Engel,L.; Walker,D.; McClenaghan,L.; Alam,R.S.; Hasan,K.; McKenzie,P.; Webby,R.J.; Webster,R.G.
EPI_ISL_78703	HA	India	2003-Apr-14	A/chicken/Chandigarh/2048/2003	NA	H9	Import from public-domain	Tosh,C.; Nagarajan,S.; Behera,P.; Pradhan,H.K.; Dubey,S.C.
EPI_ISL_144239	HA	Bangladesh	2010-Jan-20	A/environment/Bangladesh/5745/2010	NA	H9	Import from public-domain	Shanmuganatham,K.; Feeroz,M.; Jones-Engel,L.; Smith,G.J.D.; Fourment,M.; Walker,D.; McClenaghan,L.; Rabiul Alam,S.M.; Hasan,K.; Seiler,P.; Franks,J.; Danner,A.; Barman,S.; McKenzie,P.; Krauss,S.; Webby,R.J.; Webster,R.G.; Engel,L.J.; Alam,S.M.R.; Ferguson,A.
EPI_ISL_379971	HA	Bangladesh	2017-Nov-28	A/quail/Bangladesh/34042/2017	NA	H9	Import from public-domain	Barman,S.; Turner,J.C.; Hasan,M.K.; Akhtar,S.; Franks,J.; El-Shesheny,R.; Walker,D.; Seiler,P.; Friedman,K.; Kercher,L.; Kayali,G.; Jones-Engel,L.; McKenzie,P.; Krauss,S.; Webby,R.J.; Feeroz,M.M.; Webster,R.G.
EPI_ISL_397434	HA	Bangladesh	2017-Jul-21	A/quail/Bangladesh/33163/2017	NA	H9	Import from public-domain	Barman,S.; Turner,J.C.; Hasan,M.K.; Akhtar,S.; Franks,J.; El-Shesheny,R.; Walker,D.; Seiler,P.; Friedman,K.; Kercher,L.; Kayali,G.; Jones-Engel,L.; McKenzie,P.; Krauss,S.; Webby,R.J.; Feeroz,M.; Webster,R.G.
EPI_ISL_142873	HA	Bangladesh	2009-Mar-05	A/duck/Bangladesh/1009/2009	icddr,b International Centre for Diarrhoeal Disease Research, Bangladesh	H9	Centers for Disease Control and Prevention	NA
EPI_ISL_144278	HA	Bangladesh	2010-Aug-06	A/chicken/Bangladesh/8413/2010	NA	H9	Import from public-domain	Shanmuganatham,K.; Feeroz,M.; Jones-Engel,L.; Smith,G.J.D.; Fourment,M.; Walker,D.; McClenaghan,L.; Rabiul Alam,S.M.; Hasan,K.; Seiler,P.; Franks,J.; Danner,A.; Barman,S.; McKenzie,P.; Krauss,S.; Webby,R.J.; Webster,R.G.; Engel,L.J.; Alam,S.M.R.; Ferguson,A.
EPI_ISL_144261	HA	Bangladesh	2010-Oct-15	A/chicken/Bangladesh/9029/2010	NA	H9	Import from public-domain	Shanmuganatham,K.; Feeroz,M.; Jones-Engel,L.; Smith,G.J.D.; Fourment,M.; Walker,D.; McClenaghan,L.; Rabiul Alam,S.M.; Hasan,K.; Seiler,P.; Franks,J.; Danner,A.; Barman,S.; McKenzie,P.; Krauss,S.; Webby,R.J.; Webster,R.G.; Engel,L.J.; Alam,S.M.R.; Ferguson,A.
EPI_ISL_329585	HA	Bangladesh	2017-Jul-21	A/duck/Bangladesh/33132/2017	NA	H9	Import from public-domain	Barman,S.; Turner,J.C.; Hasan,M.K.; Akhtar,S.; Franks,J.; El-Shesheny,R.; Walker,D.; Seiler,P.; Friedman,K.; Kercher,L.; Kayali,G.; Jones-Engel,L.; McKenzie,P.; Krauss,S.; Webby,R.J.; Feeroz,M.; Webster,R.G.
EPI_ISL_503536	HA	Bangladesh	2019-Sep-13	A/chicken/Bangladesh/40819/2019	NA	H9	Import from public-domain	Barman,S.; Turner,J.C.; Hasan,M.; Akhtar,S.; Franks,J.; El-Shesheny,R.; Walker,D.; Seiler,P.; Mukherjee,N.; Kercher,L.; McKenzie,P.; Feeroz,M.; Webby,R.J.
EPI_ISL_257068	HA	Bangladesh	2015-Mar-22	A/chicken/Bangladesh/24947/2015	NA	H9	Import from public-domain	Shanmuganatham,K.; Barman,S.; Marinova-Petkova,A.; Hasan,M.K.; Akhtar,S.; Turner,J.C.; Franks,J.; Walker,D.; Seiler,P.; Friedman,K.; Jones-Engel,L.; McKenzie,P.; Krauss,S.; Webby,R.J.; Feeroz,M.M.; Webster,R.G.
EPI_ISL_257084	HA	Bangladesh	2016-Feb-03	A/quail/Bangladesh/28122/2016	NA	H9	Import from public-domain	Barman,S.; Marinova-Petkova,A.; Hasan,M.K.; Akhtar,S.; Turner,J.C.; Franks,J.; Walker,D.; Seiler,P.; Friedman,K.; Kercher,L.; Kayali,G.; Jones-Engel,L.; McKenzie,P.; Krauss,S.; Webby,R.J.; Feeroz,M.M.; Webster,R.G.
EPI_ISL_161216	HA	Bangladesh	2012-Oct-21	A/chicken/Bangladesh/18283/2012	NA	H9	Import from public-domain	Shanmuganatham,K.; Feeroz,M.; Jones Engel,L.; Walker,D.; McClenaghan,L.; Alam,R.S.; Hasan,K.; McKenzie,P.; Webby,R.J.; Webster,R.G.
EPI_ISL_256789	HA	Bangladesh	2014-Dec-29	A/quail/Bangladesh/24008/2014	NA	H9	Import from public-domain	Shanmuganatham,K.; Barman,S.; Marinova-Petkova,A.; Hasan,M.K.; Akhtar,S.; Turner,J.C.; Franks,J.; Walker,D.; Seiler,P.; Friedman,K.; Jones-Engel,L.; McKenzie,P.; Krauss,S.; Webby,R.J.; Feeroz,M.M.; Webster,R.G.
EPI_ISL_256787	HA	Bangladesh	2014-Oct-19	A/chicken/Bangladesh/23727/2014	NA	H9	Import from public-domain	Shanmuganatham,K.; Barman,S.; Marinova-Petkova,A.; Hasan,M.K.; Akhtar,S.; Turner,J.C.; Franks,J.; Walker,D.; Seiler,P.; Friedman,K.; Jones-Engel,L.; McKenzie,P.; Krauss,S.; Webby,R.J.; Feeroz,M.M.; Webster,R.G.
EPI_ISL_257061	HA	Bangladesh	2015-Dec-23	A/chicken/Bangladesh/27871/2015	NA	H9	Import from public-domain	Barman,S.; Marinova-Petkova,A.; Hasan,M.K.; Akhtar,S.; Turner,J.C.; Franks,J.; Walker,D.; Seiler,P.; Friedman,K.; Kercher,L.; Kayali,G.; Jones-Engel,L.; McKenzie,P.; Krauss,S.; Webby,R.J.; Feeroz,M.M.; Webster,R.G.
EPI_ISL_78704	HA	India	2004-Sep-13	A/chicken/Gujarat/3697/2004	NA	H9	Import from public-domain	Tosh,C.; Nagarajan,S.; Behera,P.; Pradhan,H.K.; Dubey,S.C.
EPI_ISL_306654	HA	Bangladesh	2017-Mar-28	A/chicken/Bangladesh/32390/2017	NA	H9	Import from public-domain	Barman,S.; Turner,J.C.; Hasan,M.K.; Akhtar,S.; Franks,J.; El-Shesheny,R.; Walker,D.; Seiler,P.; Friedman,K.; Kercher,L.; Kayali,G.; Jones-Engel,L.; McKenzie,P.; Krauss,S.; Webby,R.J.; Feeroz,M.M.; Webster,R.G.
EPI_ISL_144263	HA	Bangladesh	2010-Sep-01	A/environment/Bangladesh/8463/2010	NA	H9	Import from public-domain	Shanmuganatham,K.; Feeroz,M.; Jones-Engel,L.; Smith,G.J.D.; Fourment,M.; Walker,D.; McClenaghan,L.; Rabiul Alam,S.M.; Hasan,K.; Seiler,P.; Franks,J.; Danner,A.; Barman,S.; McKenzie,P.; Krauss,S.; Webby,R.J.; Webster,R.G.; Engel,L.J.; Alam,S.M.R.; Ferguson,A.
EPI_ISL_161226	HA	Bangladesh	2013-Feb-14	A/chicken/Bangladesh/19145/2013	NA	H9	Import from public-domain	Shanmuganatham,K.; Feeroz,M.; Jones Engel,L.; Walker,D.; McClenaghan,L.; Alam,R.S.; Hasan,K.; McKenzie,P.; Webby,R.J.; Webster,R.G.
EPI_ISL_161234	HA	Bangladesh	2013-Jan-28	A/environment/Bangladesh/18894/2013	NA	H9	Import from public-domain	Shanmuganatham,K.; Feeroz,M.; Jones Engel,L.; Walker,D.; McClenaghan,L.; Alam,R.S.; Hasan,K.; McKenzie,P.; Webby,R.J.; Webster,R.G.
EPI_ISL_161686	HA	Bangladesh	2013-Nov-24	A/quail/Bangladesh/21247/2013	NA	H9	Import from public-domain	Shanmuganatham,K.; Feeroz,M.; Jones Engel,L.; Walker,D.; McClenaghan,L.; Alam,R.S.; Hasan,K.; McKenzie,P.; Webby,R.J.; Webster,R.G.
EPI_ISL_379973	HA	Bangladesh	2017-Dec-25	A/quail/Bangladesh/34294/2017	NA	H9	Import from public-domain	Barman,S.; Turner,J.C.; Hasan,M.K.; Akhtar,S.; Franks,J.; El-Shesheny,R.; Walker,D.; Seiler,P.; Friedman,K.; Kercher,L.; Kayali,G.; Jones-Engel,L.; McKenzie,P.; Krauss,S.; Webby,R.J.; Feeroz,M.M.; Webster,R.G.
EPI_ISL_257044	HA	Bangladesh	2015-Sep-12	A/chicken/Bangladesh/26223/2015	NA	H9	Import from public-domain	Barman,S.; Marinova-Petkova,A.; Hasan,M.K.; Akhtar,S.; Turner,J.C.; Franks,J.; Walker,D.; Seiler,P.; Friedman,K.; Kercher,L.; Kayali,G.; Jones-Engel,L.; McKenzie,P.; Krauss,S.; Webby,R.J.; Feeroz,M.M.; Webster,R.G.
EPI_ISL_387995	HA	Bangladesh	2018-Dec-15	A/chicken/Bangladesh/36009/2018	NA	H9	Import from public-domain	Barman,S.; Turner,J.C.; Hasan,M.; Akhtar,S.; Franks,J.; El-Shesheny,R.; Walker,D.; Seiler,P.; Friedman,K.; Kercher,L.; McKenzie,P.; Webby,R.J.; Feeroz,M.; Webster,R.G.
EPI_ISL_329594	HA	Bangladesh	2017-May-20	A/chicken/Bangladesh/32754/2017	NA	H9	Import from public-domain	Barman,S.; Turner,J.C.; Hasan,M.K.; Akhtar,S.; Franks,J.; El-Shesheny,R.; Walker,D.; Seiler,P.; Friedman,K.; Kercher,L.; Kayali,G.; Jones-Engel,L.; McKenzie,P.; Krauss,S.; Webby,R.J.; Feeroz,M.; Webster,R.G.
EPI_ISL_256791	HA	Bangladesh	2014-Dec-29	A/quail/Bangladesh/24007/2014	NA	H9	Import from public-domain	Shanmuganatham,K.; Barman,S.; Marinova-Petkova,A.; Hasan,M.K.; Akhtar,S.; Turner,J.C.; Franks,J.; Walker,D.; Seiler,P.; Friedman,K.; Jones-Engel,L.; McKenzie,P.; Krauss,S.; Webby,R.J.; Feeroz,M.M.; Webster,R.G.
EPI_ISL_144240	HA	Bangladesh	2011-Aug-14	A/environment/Bangladesh/12103/2011	NA	H9	Import from public-domain	Shanmuganatham,K.; Feeroz,M.; Jones-Engel,L.; Smith,G.J.D.; Fourment,M.; Walker,D.; McClenaghan,L.; Rabiul Alam,S.M.; Hasan,K.; Seiler,P.; Franks,J.; Danner,A.; Barman,S.; McKenzie,P.; Krauss,S.; Webby,R.J.; Webster,R.G.; Engel,L.J.; Alam,S.M.R.; Ferguson,A.
EPI_ISL_257100	HA	Bangladesh	2015-Mar-22	A/environment/Bangladesh/24969/2015	NA	H9	Import from public-domain	Shanmuganatham,K.; Barman,S.; Marinova-Petkova,A.; Hasan,M.K.; Akhtar,S.; Turner,J.C.; Franks,J.; Walker,D.; Seiler,P.; Friedman,K.; Jones-Engel,L.; McKenzie,P.; Krauss,S.; Webby,R.J.; Feeroz,M.M.; Webster,R.G.
EPI_ISL_503506	HA	Bangladesh	2019-Feb-19	A/chicken/Bangladesh/38150/2019	NA	H9	Import from public-domain	Barman,S.; Turner,J.C.; Hasan,M.; Akhtar,S.; Franks,J.; El-Shesheny,R.; Walker,D.; Seiler,P.; Mukherjee,N.; Kercher,L.; McKenzie,P.; Feeroz,M.; Webby,R.J.
EPI_ISL_503285	HA	Bangladesh	2017-Apr-23	A/chicken/Bangladesh/NRL-AI-3238/2017	NA	H9	Import from public-domain	Kwon,J.; Criado,M.F.; Killmaster,L.; Swayne,D.E.
EPI_ISL_165820	HA	Bangladesh	2012-Nov-19	A/avian/Bangladesh/91254/2012	Institute of Epidemiology Disease Control and Research (IEDCR) & Bangladesh National Influenza Centre (NIC)	H9	Centers for Disease Control and Prevention	Gerloff, Nancy; Simpson, Natosha; Davis, C. Todd
EPI_ISL_257101	HA	Bangladesh	2015-Aug-25	A/quail/Bangladesh/25997/2015	NA	H9	Import from public-domain	Barman,S.; Marinova-Petkova,A.; Hasan,M.K.; Akhtar,S.; Turner,J.C.; Franks,J.; Walker,D.; Seiler,P.; Friedman,K.; Kercher,L.; Kayali,G.; Jones-Engel,L.; McKenzie,P.; Krauss,S.; Webby,R.J.; Feeroz,M.M.; Webster,R.G.
EPI_ISL_329596	HA	Bangladesh	2017-Jun-11	A/chicken/Bangladesh/32958/2017	NA	H9	Import from public-domain	Barman,S.; Turner,J.C.; Hasan,M.K.; Akhtar,S.; Franks,J.; El-Shesheny,R.; Walker,D.; Seiler,P.; Friedman,K.; Kercher,L.; Kayali,G.; Jones-Engel,L.; McKenzie,P.; Krauss,S.; Webby,R.J.; Feeroz,M.; Webster,R.G.
EPI_ISL_144229	HA	Bangladesh	2010-Sep-01	A/chicken/Bangladesh/8731/2010	NA	H9	Import from public-domain	Shanmuganatham,K.; Feeroz,M.; Jones-Engel,L.; Smith,G.J.D.; Fourment,M.; Walker,D.; McClenaghan,L.; Rabiul Alam,S.M.; Hasan,K.; Seiler,P.; Franks,J.; Danner,A.; Barman,S.; McKenzie,P.; Krauss,S.; Webby,R.J.; Webster,R.G.; Engel,L.J.; Alam,S.M.R.; Ferguson,A.
EPI_ISL_257091	HA	Bangladesh	2015-Jan-23	A/chicken/Bangladesh/24249/2015	NA	H9	Import from public-domain	Shanmuganatham,K.; Barman,S.; Marinova-Petkova,A.; Hasan,M.K.; Akhtar,S.; Turner,J.C.; Franks,J.; Walker,D.; Seiler,P.; Friedman,K.; Jones-Engel,L.; McKenzie,P.; Krauss,S.; Webby,R.J.; Feeroz,M.M.; Webster,R.G.

EPI_ISL_257106	HA	Bangladesh	2015-Aug-25	A/quail/Bangladesh/25992/2015	NA	H9	Import from public-domain	Barman,S.; Marinova-Petkova,A.; Hasan,M.K.; Akhtar,S.; Turner,J.C.; Franks,J.; Walker,D.; Seiler,P.; Friedman,K.; Kercher,L.; Kayali,G.; Jones-Engel,L.; McKenzie,P.; Krauss,S.; Webby,R.J.; Feeroz,M.M.; Webster,R.G.
EPI_ISL_144266	HA	Bangladesh	2010-Oct-15	A/chicken/Bangladesh/8996/2010	NA	H9	Import from public-domain	Shanmuganatham,K.; Feeroz,M.; Jones-Engel,L.; Smith,G.J.D.; Fourment,M.; Walker,D.; McClenaghan,L.; Rabiul Alam,S.M.; Hasan,K.; Seiler,P.; Franks,J.; Danner,A.; Barman,S.; McKenzie,P.; Krauss,S.; Webby,R.J.; Webster,R.G.; Engel,L.J.; Alam,S.M.R.; Ferguson,A.
EPI_ISL_144284	HA	Bangladesh	2011-Jun-30	A/chicken/Bangladesh/11309/2011	NA	H9	Import from public-domain	Shanmuganatham,K.; Feeroz,M.; Jones-Engel,L.; Smith,G.J.D.; Fourment,M.; Walker,D.; McClenaghan,L.; Rabiul Alam,S.M.; Hasan,K.; Seiler,P.; Franks,J.; Danner,A.; Barman,S.; McKenzie,P.; Krauss,S.; Webby,R.J.; Webster,R.G.; Engel,L.J.; Alam,S.M.R.; Ferguson,A.
EPI_ISL_306665	HA	Bangladesh	2017-Mar-29	A/chicken/Bangladesh/32287/2017	NA	H9	Import from public-domain	Barman,S.; Turner,J.C.; Hasan,M.K.; Akhtar,S.; Franks,J.; El-Shesheny,R.; Walker,D.; Seiler,P.; Friedman,K.; Kercher,L.; Kayali,G.; Jones-Engel,L.; McKenzie,P.; Krauss,S.; Webby,R.J.; Feeroz,M.M.; Webster,R.G.
EPI_ISL_379690	HA	Bangladesh	2018-Jan-23	A/environment/Bangladesh/34573/2018	NA	H9	Import from public-domain	Barman,S.; Turner,J.C.; Hasan,M.K.; Akhtar,S.; Franks,J.; El-Shesheny,R.; Walker,D.; Seiler,P.; Friedman,K.; Kercher,L.; Kayali,G.; Jones-Engel,L.; McKenzie,P.; Krauss,S.; Webby,R.J.; Feeroz,M.M.; Webster,R.G.
EPI_ISL_387979	HA	Bangladesh	2018-Dec-18	A/duck/Bangladesh/35996/2018	NA	H9	Import from public-domain	Barman,S.; Turner,J.C.; Hasan,M.; Akhtar,S.; Franks,J.; El-Shesheny,R.; Walker,D.; Seiler,P.; Friedman,K.; Kercher,L.; McKenzie,P.; Webby,R.J.; Feeroz,M.; Webster,R.G.
EPI_ISL_257107	HA	Bangladesh	2015-Aug-25	A/environment/Bangladesh/26032/2015	NA	H9	Import from public-domain	Barman,S.; Marinova-Petkova,A.; Hasan,M.K.; Akhtar,S.; Turner,J.C.; Franks,J.; Walker,D.; Seiler,P.; Friedman,K.; Kercher,L.; Kayali,G.; Jones-Engel,L.; McKenzie,P.; Krauss,S.; Webby,R.J.; Feeroz,M.M.; Webster,R.G.
EPI_ISL_161197	HA	Bangladesh	2013-May-25	A/chicken/Bangladesh/19870/2013	NA	H9	Import from public-domain	Shanmuganatham,K.; Feeroz,M.; Jones Engel,L.; Walker,D.; McClenaghan,L.; Alam,R.S.; Hasan,K.; McKenzie,P.; Webby,R.J.; Webster,R.G.
EPI_ISL_78707	HA	India	2004-May-25	A/chicken/Uttar Pradesh/2544/2004	NA	H9	Import from public-domain	Tosh,C.; Nagarajan,S.; Behera,P.; Pradhan,H.K.; Dubey,S.C.
EPI_ISL_165822	HA	Bangladesh	2012-Dec-15	A/poultry/Bangladesh/91354/2012	Institute of Epidemiology Disease Control and Research (IEDCR) & Bangladesh National Influenza Centre (NIC)	H9	Centers for Disease Control and Prevention	Gerloff, Nancy; Simpson, Natosha; Davis, C. Todd
EPI_ISL_144281	HA	Bangladesh	2011-Feb-08	A/environment/Bangladesh/10234/2011	NA	H9	Import from public-domain	Shanmuganatham,K.; Feeroz,M.; Jones-Engel,L.; Smith,G.J.D.; Fourment,M.; Walker,D.; McClenaghan,L.; Rabiul Alam,S.M.; Hasan,K.; Seiler,P.; Franks,J.; Danner,A.; Barman,S.; McKenzie,P.; Krauss,S.; Webby,R.J.; Webster,R.G.; Engel,L.J.; Alam,S.M.R.; Ferguson,A.
EPI_ISL_161235	HA	Bangladesh	2012-Oct-21	A/chicken/Bangladesh/18224/2012	NA	H9	Import from public-domain	Shanmuganatham,K.; Feeroz,M.; Jones Engel,L.; Walker,D.; McClenaghan,L.; Alam,R.S.; Hasan,K.; McKenzie,P.; Webby,R.J.; Webster,R.G.
EPI_ISL_329559	HA	Bangladesh	2017-Sep-28	A/chicken/Bangladesh/33645/2017	NA	H9	Import from public-domain	Barman,S.; Turner,J.C.; Hasan,M.K.; Akhtar,S.; Franks,J.; El-Shesheny,R.; Walker,D.; Seiler,P.; Friedman,K.; Kercher,L.; Kayali,G.; Jones-Engel,L.; McKenzie,P.; Krauss,S.; Webby,R.J.; Feeroz,M.; Webster,R.G.
EPI_ISL_144275	HA	Bangladesh	2011-Nov-24	A/chicken/Bangladesh/13962/2011	NA	H9	Import from public-domain	Shanmuganatham,K.; Feeroz,M.; Jones-Engel,L.; Smith,G.J.D.; Fourment,M.; Walker,D.; McClenaghan,L.; Rabiul Alam,S.M.; Hasan,K.; Seiler,P.; Franks,J.; Danner,A.; Barman,S.; McKenzie,P.; Krauss,S.; Webby,R.J.; Webster,R.G.; Engel,L.J.; Alam,S.M.R.; Ferguson,A.
EPI_ISL_503494	HA	Bangladesh	2019-Feb-19	A/chicken/Bangladesh/38208/2019	NA	H9	Import from public-domain	Barman,S.; Turner,J.C.; Hasan,M.; Akhtar,S.; Franks,J.; El-Shesheny,R.; Walker,D.; Seiler,P.; Mukherjee,N.; Kercher,L.; McKenzie,P.; Feeroz,M.; Webby,R.J.
EPI_ISL_27601	HA	India	2003-Apr-25	A/chicken/Punjab/2063/2003	NA	H9	Import from public-domain	
EPI1581764	HA	Bangladesh	2018-Jun-05	A/duck/Bangladesh/35434/2018	NA	H5	Import from public-domain	Barman,S.; Turner,J.C.; Hasan,M.; Akhtar,S.; Franks,J.; El-Shesheny,R.; Walker,D.; Seiler,P.; Friedman,K.; Kercher,L.; McKenzie,P.; Webby,R.J.; Feeroz,M.; Webster,R.G.
EPI1581743	HA	Bangladesh	2018-Apr-27	A/duck/Bangladesh/35346/2018	NA	H5	Import from public-domain	Barman,S.; Turner,J.C.; Hasan,M.; Akhtar,S.; Franks,J.; El-Shesheny,R.; Walker,D.; Seiler,P.; Friedman,K.; Kercher,L.; McKenzie,P.; Webby,R.J.; Feeroz,M.; Webster,R.G.
EPI1581730	HA	Bangladesh	2018-Jun-05	A/duck/Bangladesh/35435/2018	NA	H5	Import from public-domain	Barman,S.; Turner,J.C.; Hasan,M.; Akhtar,S.; Franks,J.; El-Shesheny,R.; Walker,D.; Seiler,P.; Friedman,K.; Kercher,L.; McKenzie,P.; Webby,R.J.; Feeroz,M.; Webster,R.G.
EPI1581728	HA	Bangladesh	2018-Apr-27	A/duck/Bangladesh/35343/2018	NA	H5	Import from public-domain	Barman,S.; Turner,J.C.; Hasan,M.; Akhtar,S.; Franks,J.; El-Shesheny,R.; Walker,D.; Seiler,P.; Friedman,K.; Kercher,L.; McKenzie,P.; Webby,R.J.; Feeroz,M.; Webster,R.G.
EPI1581721	HA	Bangladesh	2018-Jun-05	A/duck/Bangladesh/35438/2018	NA	H5	Import from public-domain	Barman,S.; Turner,J.C.; Hasan,M.; Akhtar,S.; Franks,J.; El-Shesheny,R.; Walker,D.; Seiler,P.; Friedman,K.; Kercher,L.; McKenzie,P.; Webby,R.J.; Feeroz,M.; Webster,R.G.
EPI1581704	HA	Bangladesh	2018-Apr-26	A/duck/Bangladesh/35293/2018	NA	H5	Import from public-domain	Barman,S.; Turner,J.C.; Hasan,M.; Akhtar,S.; Franks,J.; El-Shesheny,R.; Walker,D.; Seiler,P.; Friedman,K.; Kercher,L.; McKenzie,P.; Webby,R.J.; Feeroz,M.; Webster,R.G.
EPI1581671	HA	Bangladesh	2018-Jun-05	A/duck/Bangladesh/35437/2018	NA	H5	Import from public-domain	Barman,S.; Turner,J.C.; Hasan,M.; Akhtar,S.; Franks,J.; El-Shesheny,R.; Walker,D.; Seiler,P.; Friedman,K.; Kercher,L.; McKenzie,P.; Webby,R.J.; Feeroz,M.; Webster,R.G.
EPI1581665	HA	Bangladesh	2018-Apr-28	A/duck/Bangladesh/35389/2018	NA	H5	Import from public-domain	Barman,S.; Turner,J.C.; Hasan,M.; Akhtar,S.; Franks,J.; El-Shesheny,R.; Walker,D.; Seiler,P.; Friedman,K.; Kercher,L.; McKenzie,P.; Webby,R.J.; Feeroz,M.; Webster,R.G.
EPI1581655	HA	Bangladesh	2018-Aug-09	A/duck/Bangladesh/35723/2018	NA	H5	Import from public-domain	Barman,S.; Turner,J.C.; Hasan,M.; Akhtar,S.; Franks,J.; El-Shesheny,R.; Walker,D.; Seiler,P.; Friedman,K.; Kercher,L.; McKenzie,P.; Webby,R.J.; Feeroz,M.; Webster,R.G.
EPI1581652	HA	Bangladesh	2018-Jun-05	A/duck/Bangladesh/35439/2018	NA	H5	Import from public-domain	Barman,S.; Turner,J.C.; Hasan,M.; Akhtar,S.; Franks,J.; El-Shesheny,R.; Walker,D.; Seiler,P.; Friedman,K.; Kercher,L.; McKenzie,P.; Webby,R.J.; Feeroz,M.; Webster,R.G.
EPI1581644	HA	Bangladesh	2018-Oct-30	A/duck/Bangladesh/35829/2018	NA	H5	Import from public-domain	Barman,S.; Turner,J.C.; Hasan,M.; Akhtar,S.; Franks,J.; El-Shesheny,R.; Walker,D.; Seiler,P.; Friedman,K.; Kercher,L.; McKenzie,P.; Webby,R.J.; Feeroz,M.; Webster,R.G.
EPI1581635	HA	Bangladesh	2018-Aug-09	A/duck/Bangladesh/35602/2018	NA	H5	Import from public-domain	Barman,S.; Turner,J.C.; Hasan,M.; Akhtar,S.; Franks,J.; El-Shesheny,R.; Walker,D.; Seiler,P.; Friedman,K.; Kercher,L.; McKenzie,P.; Webby,R.J.; Feeroz,M.; Webster,R.G.
EPI1581630	HA	Bangladesh	2018-Jun-05	A/duck/Bangladesh/35440/2018	NA	H5	Import from public-domain	Barman,S.; Turner,J.C.; Hasan,M.; Akhtar,S.; Franks,J.; El-Shesheny,R.; Walker,D.; Seiler,P.; Friedman,K.; Kercher,L.; McKenzie,P.; Webby,R.J.; Feeroz,M.; Webster,R.G.
EPI1581626	HA	Bangladesh	2018-Aug-09	A/duck/Bangladesh/35672/2018	NA	H5	Import from public-domain	Barman,S.; Turner,J.C.; Hasan,M.; Akhtar,S.; Franks,J.; El-Shesheny,R.; Walker,D.; Seiler,P.; Friedman,K.; Kercher,L.; McKenzie,P.; Webby,R.J.; Feeroz,M.; Webster,R.G.
EPI1581618	HA	Bangladesh	2018-Aug-09	A/chicken/Bangladesh/35579/2018	NA	H5	Import from public-domain	Barman,S.; Turner,J.C.; Hasan,M.; Akhtar,S.; Franks,J.; El-Shesheny,R.; Walker,D.; Seiler,P.; Friedman,K.; Kercher,L.; McKenzie,P.; Webby,R.J.; Feeroz,M.; Webster,R.G.
EPI1581597	HA	Bangladesh	2018-Aug-09	A/duck/Bangladesh/35600/2018	NA	H5	Import from public-domain	Barman,S.; Turner,J.C.; Hasan,M.; Akhtar,S.; Franks,J.; El-Shesheny,R.; Walker,D.; Seiler,P.; Friedman,K.; Kercher,L.; McKenzie,P.; Webby,R.J.; Feeroz,M.; Webster,R.G.
EPI1581581	HA	Bangladesh	2018-Dec-18	A/duck/Bangladesh/35921/2018	NA	H5	Import from public-domain	Barman,S.; Turner,J.C.; Hasan,M.; Akhtar,S.; Franks,J.; El-Shesheny,R.; Walker,D.; Seiler,P.; Friedman,K.; Kercher,L.; McKenzie,P.; Webby,R.J.; Feeroz,M.; Webster,R.G.
EPI1581566	HA	Bangladesh	2018-Oct-30	A/duck/Bangladesh/35827/2018	NA	H5	Import from public-domain	Barman,S.; Turner,J.C.; Hasan,M.; Akhtar,S.; Franks,J.; El-Shesheny,R.; Walker,D.; Seiler,P.; Friedman,K.; Kercher,L.; McKenzie,P.; Webby,R.J.; Feeroz,M.; Webster,R.G.
EPI1581552	HA	Bangladesh	2018-Oct-30	A/duck/Bangladesh/35835/2018	NA	H5	Import from public-domain	Barman,S.; Turner,J.C.; Hasan,M.; Akhtar,S.; Franks,J.; El-Shesheny,R.; Walker,D.; Seiler,P.; Friedman,K.; Kercher,L.; McKenzie,P.; Webby,R.J.; Feeroz,M.; Webster,R.G.
EPI1581539	HA	Bangladesh	2018-Oct-30	A/duck/Bangladesh/35833/2018	NA	H5	Import from public-domain	Barman,S.; Turner,J.C.; Hasan,M.; Akhtar,S.; Franks,J.; El-Shesheny,R.; Walker,D.; Seiler,P.; Friedman,K.; Kercher,L.; McKenzie,P.; Webby,R.J.; Feeroz,M.; Webster,R.G.
EPI1581522	HA	Bangladesh	2018-Dec-18	A/duck/Bangladesh/35924/2018	NA	H5	Import from public-domain	Barman,S.; Turner,J.C.; Hasan,M.; Akhtar,S.; Franks,J.; El-Shesheny,R.; Walker,D.; Seiler,P.; Friedman,K.; Kercher,L.; McKenzie,P.; Webby,R.J.; Feeroz,M.; Webster,R.G.
EPI1581469	HA	Bangladesh	2018-Dec-18	A/duck/Bangladesh/35986/2018	NA	H5	Import from public-domain	Barman,S.; Turner,J.C.; Hasan,M.; Akhtar,S.; Franks,J.; El-Shesheny,R.; Walker,D.; Seiler,P.; Friedman,K.; Kercher,L.; McKenzie,P.; Webby,R.J.; Feeroz,M.; Webster,R.G.
EPI1581416	HA	Bangladesh	2018-Dec-15	A/duck/Bangladesh/36037/2018	NA	H5	Import from public-domain	Barman,S.; Turner,J.C.; Hasan,M.; Akhtar,S.; Franks,J.; El-Shesheny,R.; Walker,D.; Seiler,P.; Friedman,K.; Kercher,L.; McKenzie,P.; Webby,R.J.; Feeroz,M.; Webster,R.G.
EPI1581407	HA	Bangladesh	2018-Dec-15	A/duck/Bangladesh/36042/2018	NA	H5	Import from public-domain	Barman,S.; Turner,J.C.; Hasan,M.; Akhtar,S.; Franks,J.; El-Shesheny,R.; Walker,D.; Seiler,P.; Friedman,K.; Kercher,L.; McKenzie,P.; Webby,R.J.; Feeroz,M.; Webster,R.G.
EPI1581349	HA	Bangladesh	2018-Apr-26	A/duck/Bangladesh/35222/2018	NA	H5	Import from public-domain	Barman,S.; Turner,J.C.; Hasan,M.; Akhtar,S.; Franks,J.; El-Shesheny,R.; Walker,D.; Seiler,P.; Friedman,K.; Kercher,L.; McKenzie,P.; Webby,R.J.; Feeroz,M.; Webster,R.G.
EPI1581333	HA	Bangladesh	2018-Apr-26	A/duck/Bangladesh/35226/2018	NA	H5	Import from public-domain	Barman,S.; Turner,J.C.; Hasan,M.; Akhtar,S.; Franks,J.; El-Shesheny,R.; Walker,D.; Seiler,P.; Friedman,K.; Kercher,L.; McKenzie,P.; Webby,R.J.; Feeroz,M.; Webster,R.G.
EPI965217	HA	Bangladesh	2015-Sep-12	A/environment/Bangladesh/26170/2015	NA	H5	Import from public-domain	Barman,S.; Marinova-Petkova,A.; Hasan,M.K.; Akhtar,S.; Turner,J.C.; Franks,J.; Walker,D.; Seiler,P.; Friedman,K.; Kercher,L.; Kayali,G.; Jones-Engel,L.; McKenzie,P.; Krauss,S.; Webby,R.J.; Feeroz,M.M.; Webster,R.G.

EPI965041	HA	Bangladesh	2014-Aug-17	A/environment/Bangladesh/23223/2014	NA	H5	Import from public-domain	Marinova-Petkova,A.; Shanmuganatham,K.; Feeroz,M.M.; Jones-Engel,L.; Hasan,M.K.; Akhtar,S.; Turner,J.; Walker,D.; Seiler,P.; Franks,J.; McKenzie,P.; Krauss,S.; Webby,R.J.; Webster,R.G.; Hasan,K.; Seiler,J.
EPI542317	HA	Bangladesh	2010-Dec-01	A/migratory bird/Bangladesh/P29/2010	NA	H5	Import from public-domain	Parvin,R.; Kamal,A.H.M.; Haque,M.E.; Chowdhury,E.H.; Giasuddin,M.; Islam,M.R.; Vahlenkamp,T.W.
EPI542316	HA	Bangladesh	2010-Dec-01	A/migratory bird/Bangladesh/P18/2010	NA	H5	Import from public-domain	Parvin,R.; Kamal,A.H.M.; Haque,M.E.; Chowdhury,E.H.; Giasuddin,M.; Islam,M.R.; Vahlenkamp,T.W.
EPI510500	HA	Bangladesh	2012-Jan-18	A/environment/Bangladesh/15145/2012	NA	H5	Import from public-domain	Marinova-Petkova,A.; Feeroz,M.M.; Alam,S.M.R.; Hasan,M.K.; Akhtar,S.; Jones-Engel,L.; Walker,D.; McClenaghan,L.; Rubrum,A.; Franks,J.; Seiler,P.; Jeevan,T.; McKenzie,P.; Krauss,S.; Webby,R.J.; Webster,R.G.
EPI510493	HA	Bangladesh	2012-Jan-18	A/environment/Bangladesh/15165/2012	NA	H5	Import from public-domain	Marinova-Petkova,A.; Feeroz,M.M.; Alam,S.M.R.; Hasan,M.K.; Akhtar,S.; Jones-Engel,L.; Walker,D.; McClenaghan,L.; Rubrum,A.; Franks,J.; Seiler,P.; Jeevan,T.; McKenzie,P.; Krauss,S.; Webby,R.J.; Webster,R.G.
EPI510486	HA	Bangladesh	2012-Jan-18	A/environment/Bangladesh/15121/2012	NA	H5	Import from public-domain	Marinova-Petkova,A.; Feeroz,M.M.; Alam,S.M.R.; Hasan,M.K.; Akhtar,S.; Jones-Engel,L.; Walker,D.; McClenaghan,L.; Rubrum,A.; Franks,J.; Seiler,P.; Jeevan,T.; McKenzie,P.; Krauss,S.; Webby,R.J.; Webster,R.G.
EPI510480	HA	Bangladesh	2012-Jan-18	A/environment/Bangladesh/15105/2012	NA	H5	Import from public-domain	Marinova-Petkova,A.; Feeroz,M.M.; Alam,S.M.R.; Hasan,M.K.; Akhtar,S.; Jones-Engel,L.; Walker,D.; McClenaghan,L.; Rubrum,A.; Franks,J.; Seiler,P.; Jeevan,T.; McKenzie,P.; Krauss,S.; Webby,R.J.; Webster,R.G.
EPI510472	HA	Bangladesh	2013-Mar-30	A/environment/Bangladesh/19338/2013	NA	H5	Import from public-domain	Marinova-Petkova,A.; Feeroz,M.M.; Alam,S.M.R.; Hasan,M.K.; Akhtar,S.; Jones-Engel,L.; Walker,D.; McClenaghan,L.; Rubrum,A.; Franks,J.; Seiler,P.; Jeevan,T.; McKenzie,P.; Krauss,S.; Webby,R.J.; Webster,R.G.
EPI510466	HA	Bangladesh	2011-Jan-18	A/environment/Bangladesh/9675/2011	NA	H5	Import from public-domain	Marinova-Petkova,A.; Feeroz,M.M.; Alam,S.M.R.; Hasan,M.K.; Akhtar,S.; Jones-Engel,L.; Walker,D.; McClenaghan,L.; Rubrum,A.; Franks,J.; Seiler,P.; Jeevan,T.; McKenzie,P.; Krauss,S.; Webby,R.J.; Webster,R.G.
EPI510455	HA	Bangladesh	2012-Jan-18	A/chicken/Bangladesh/15078/2012	NA	H5	Import from public-domain	Marinova-Petkova,A.; Feeroz,M.M.; Alam,S.M.R.; Hasan,M.K.; Akhtar,S.; Jones-Engel,L.; Walker,D.; McClenaghan,L.; Rubrum,A.; Franks,J.; Seiler,P.; Jeevan,T.; McKenzie,P.; Krauss,S.; Webby,R.J.; Webster,R.G.
EPI510450	HA	Bangladesh	2013-Jan-28	A/environment/Bangladesh/18948/2013	NA	H5	Import from public-domain	Marinova-Petkova,A.; Feeroz,M.M.; Alam,S.M.R.; Hasan,M.K.; Akhtar,S.; Jones-Engel,L.; Walker,D.; McClenaghan,L.; Rubrum,A.; Franks,J.; Seiler,P.; Jeevan,T.; McKenzie,P.; Krauss,S.; Webby,R.J.; Webster,R.G.
EPI510448	HA	Bangladesh	2013-Mar-30	A/quail/Bangladesh/19254/2013	NA	H5	Import from public-domain	Marinova-Petkova,A.; Feeroz,M.M.; Alam,S.M.R.; Hasan,M.K.; Akhtar,S.; Jones-Engel,L.; Walker,D.; McClenaghan,L.; Rubrum,A.; Franks,J.; Seiler,P.; Jeevan,T.; McKenzie,P.; Krauss,S.; Webby,R.J.; Webster,R.G.
EPI510442	HA	Bangladesh	2012-Jan-18	A/chicken/Bangladesh/15085/2012	NA	H5	Import from public-domain	Marinova-Petkova,A.; Feeroz,M.M.; Alam,S.M.R.; Hasan,M.K.; Akhtar,S.; Jones-Engel,L.; Walker,D.; McClenaghan,L.; Rubrum,A.; Franks,J.; Seiler,P.; Jeevan,T.; McKenzie,P.; Krauss,S.; Webby,R.J.; Webster,R.G.
EPI510434	HA	Bangladesh	2012-Jan-18	A/chicken/Bangladesh/15083/2012	NA	H5	Import from public-domain	Marinova-Petkova,A.; Feeroz,M.M.; Alam,S.M.R.; Hasan,M.K.; Akhtar,S.; Jones-Engel,L.; Walker,D.; McClenaghan,L.; Rubrum,A.; Franks,J.; Seiler,P.; Jeevan,T.; McKenzie,P.; Krauss,S.; Webby,R.J.; Webster,R.G.
EPI510426	HA	Bangladesh	2012-Oct-21	A/chicken/Bangladesh/18248/2012	NA	H5	Import from public-domain	Marinova-Petkova,A.; Feeroz,M.M.; Alam,S.M.R.; Hasan,M.K.; Akhtar,S.; Jones-Engel,L.; Walker,D.; McClenaghan,L.; Rubrum,A.; Franks,J.; Seiler,P.; Jeevan,T.; McKenzie,P.; Krauss,S.; Webby,R.J.; Webster,R.G.
EPI510418	HA	Bangladesh	2012-Jan-18	A/chicken/Bangladesh/15079/2012	NA	H5	Import from public-domain	Marinova-Petkova,A.; Feeroz,M.M.; Alam,S.M.R.; Hasan,M.K.; Akhtar,S.; Jones-Engel,L.; Walker,D.; McClenaghan,L.; Rubrum,A.; Franks,J.; Seiler,P.; Jeevan,T.; McKenzie,P.; Krauss,S.; Webby,R.J.; Webster,R.G.
EPI510412	HA	Bangladesh	2011-Aug-14	A/environment/Bangladesh/12197/2011	NA	H5	Import from public-domain	Marinova-Petkova,A.; Feeroz,M.M.; Alam,S.M.R.; Hasan,M.K.; Akhtar,S.; Jones-Engel,L.; Walker,D.; McClenaghan,L.; Rubrum,A.; Franks,J.; Seiler,P.; Jeevan,T.; McKenzie,P.; Krauss,S.; Webby,R.J.; Webster,R.G.
EPI510405	HA	Bangladesh	2012-Jan-18	A/environment/Bangladesh/15147/2012	NA	H5	Import from public-domain	Marinova-Petkova,A.; Feeroz,M.M.; Alam,S.M.R.; Hasan,M.K.; Akhtar,S.; Jones-Engel,L.; Walker,D.; McClenaghan,L.; Rubrum,A.; Franks,J.; Seiler,P.; Jeevan,T.; McKenzie,P.; Krauss,S.; Webby,R.J.; Webster,R.G.
EPI510401	HA	Bangladesh	2012-Mar-31	A/environment/Bangladesh/16298/2012	NA	H5	Import from public-domain	Marinova-Petkova,A.; Feeroz,M.M.; Alam,S.M.R.; Hasan,M.K.; Akhtar,S.; Jones-Engel,L.; Walker,D.; McClenaghan,L.; Rubrum,A.; Franks,J.; Seiler,P.; Jeevan,T.; McKenzie,P.; Krauss,S.; Webby,R.J.; Webster,R.G.
EPI510389	HA	Bangladesh	2012-Sep-07	A/chicken/Bangladesh/18061/2012	NA	H5	Import from public-domain	Marinova-Petkova,A.; Feeroz,M.M.; Alam,S.M.R.; Hasan,M.K.; Akhtar,S.; Jones-Engel,L.; Walker,D.; McClenaghan,L.; Rubrum,A.; Franks,J.; Seiler,P.; Jeevan,T.; McKenzie,P.; Krauss,S.; Webby,R.J.; Webster,R.G.
EPI510381	HA	Bangladesh	2013-Jan-28	A/environment/Bangladesh/18984/2013	NA	H5	Import from public-domain	Marinova-Petkova,A.; Feeroz,M.M.; Alam,S.M.R.; Hasan,M.K.; Akhtar,S.; Jones-Engel,L.; Walker,D.; McClenaghan,L.; Rubrum,A.; Franks,J.; Seiler,P.; Jeevan,T.; McKenzie,P.; Krauss,S.; Webby,R.J.; Webster,R.G.
EPI510376	HA	Bangladesh	2013-Jan-28	A/environment/Bangladesh/18989/2013	NA	H5	Import from public-domain	Marinova-Petkova,A.; Feeroz,M.M.; Alam,S.M.R.; Hasan,M.K.; Akhtar,S.; Jones-Engel,L.; Walker,D.; McClenaghan,L.; Rubrum,A.; Franks,J.; Seiler,P.; Jeevan,T.; McKenzie,P.; Krauss,S.; Webby,R.J.; Webster,R.G.
EPI510365	HA	Bangladesh	2012-Sep-07	A/chicken/Bangladesh/18065/2012	NA	H5	Import from public-domain	Marinova-Petkova,A.; Feeroz,M.M.; Alam,S.M.R.; Hasan,M.K.; Akhtar,S.; Jones-Engel,L.; Walker,D.; McClenaghan,L.; Rubrum,A.; Franks,J.; Seiler,P.; Jeevan,T.; McKenzie,P.; Krauss,S.; Webby,R.J.; Webster,R.G.
EPI510357	HA	Bangladesh	2012-Feb-08	A/environment/Bangladesh/15661/2012	NA	H5	Import from public-domain	Marinova-Petkova,A.; Feeroz,M.M.; Alam,S.M.R.; Hasan,M.K.; Akhtar,S.; Jones-Engel,L.; Walker,D.; McClenaghan,L.; Rubrum,A.; Franks,J.; Seiler,P.; Jeevan,T.; McKenzie,P.; Krauss,S.; Webby,R.J.; Webster,R.G.
EPI510333	HA	Bangladesh	2013-Jan-28	A/duck/Bangladesh/18949/2013	NA	H5	Import from public-domain	Marinova-Petkova,A.; Feeroz,M.M.; Alam,S.M.R.; Hasan,M.K.; Akhtar,S.; Jones-Engel,L.; Walker,D.; McClenaghan,L.; Rubrum,A.; Franks,J.; Seiler,P.; Jeevan,T.; McKenzie,P.; Krauss,S.; Webby,R.J.; Webster,R.G.
EPI510323	HA	Bangladesh	2012-Jan-18	A/environment/Bangladesh/15114/2012	NA	H5	Import from public-domain	Marinova-Petkova,A.; Feeroz,M.M.; Alam,S.M.R.; Hasan,M.K.; Akhtar,S.; Jones-Engel,L.; Walker,D.; McClenaghan,L.; Rubrum,A.; Franks,J.; Seiler,P.; Jeevan,T.; McKenzie,P.; Krauss,S.; Webby,R.J.; Webster,R.G.
EPI510303	HA	Bangladesh	2012-Jul-24	A/duck/Bangladesh/17753/2012	NA	H5	Import from public-domain	Marinova-Petkova,A.; Feeroz,M.M.; Alam,S.M.R.; Hasan,M.K.; Akhtar,S.; Jones-Engel,L.; Walker,D.; McClenaghan,L.; Rubrum,A.; Franks,J.; Seiler,P.; Jeevan,T.; McKenzie,P.; Krauss,S.; Webby,R.J.; Webster,R.G.
EPI510283	HA	Bangladesh	2012-Jan-18	A/environment/Bangladesh/15126/2012	NA	H5	Import from public-domain	Marinova-Petkova,A.; Feeroz,M.M.; Alam,S.M.R.; Hasan,M.K.; Akhtar,S.; Jones-Engel,L.; Walker,D.; McClenaghan,L.; Rubrum,A.; Franks,J.; Seiler,P.; Jeevan,T.; McKenzie,P.; Krauss,S.; Webby,R.J.; Webster,R.G.
EPI510278	HA	Bangladesh	2013-Mar-30	A/quail/Bangladesh/19250/2013	NA	H5	Import from public-domain	Marinova-Petkova,A.; Feeroz,M.M.; Alam,S.M.R.; Hasan,M.K.; Akhtar,S.; Jones-Engel,L.; Walker,D.; McClenaghan,L.; Rubrum,A.; Franks,J.; Seiler,P.; Jeevan,T.; McKenzie,P.; Krauss,S.; Webby,R.J.; Webster,R.G.
EPI510273	HA	Bangladesh	2010-Jul-10	A/chicken/Bangladesh/9636/2010	NA	H5	Import from public-domain	Marinova-Petkova,A.; Feeroz,M.M.; Alam,S.M.R.; Hasan,M.K.; Akhtar,S.; Jones-Engel,L.; Walker,D.; McClenaghan,L.; Rubrum,A.; Franks,J.; Seiler,P.; Jeevan,T.; McKenzie,P.; Krauss,S.; Webby,R.J.; Webster,R.G.
EPI510265	HA	Bangladesh	2013-Feb-14	A/duck/Bangladesh/19099/2013	NA	H5	Import from public-domain	Marinova-Petkova,A.; Feeroz,M.M.; Alam,S.M.R.; Hasan,M.K.; Akhtar,S.; Jones-Engel,L.; Walker,D.; McClenaghan,L.; Rubrum,A.; Franks,J.; Seiler,P.; Jeevan,T.; McKenzie,P.; Krauss,S.; Webby,R.J.; Webster,R.G.
EPI1778272	HA	Bangladesh	2019-Sep-13	A/duck/Bangladesh/40825/2019	NA	H5	Import from public-domain	Barman,S.; Turner,J.C.; Hasan,M.; Akhtar,S.; Franks,J.; El-Shesheny,R.; Walker,D.; Seiler,P.; Mukherjee,N.; Kercher,L.; McKenzie,P.; Feeroz,M.; Webby,R.J.
EPI1778238	HA	Bangladesh	2019-Sep-13	A/duck/Bangladesh/40832/2019	NA	H5	Import from public-domain	Barman,S.; Turner,J.C.; Hasan,M.; Akhtar,S.; Franks,J.; El-Shesheny,R.; Walker,D.; Seiler,P.; Mukherjee,N.; Kercher,L.; McKenzie,P.; Feeroz,M.; Webby,R.J.
EPI1778220	HA	Bangladesh	2019-Jul-11	A/duck/Bangladesh/40416/2019	NA	H5	Import from public-domain	Barman,S.; Turner,J.C.; Hasan,M.; Akhtar,S.; Franks,J.; El-Shesheny,R.; Walker,D.; Seiler,P.; Mukherjee,N.; Kercher,L.; McKenzie,P.; Feeroz,M.; Webby,R.J.
EPI1778209	HA	Bangladesh	2019-Sep-13	A/duck/Bangladesh/40826/2019	NA	H5	Import from public-domain	Barman,S.; Turner,J.C.; Hasan,M.; Akhtar,S.; Franks,J.; El-Shesheny,R.; Walker,D.; Seiler,P.; Mukherjee,N.; Kercher,L.; McKenzie,P.; Feeroz,M.; Webby,R.J.
EPI1778153	HA	Bangladesh	2019-Jul-10	A/duck/Bangladesh/40532/2019	NA	H5	Import from public-domain	Barman,S.; Turner,J.C.; Hasan,M.; Akhtar,S.; Franks,J.; El-Shesheny,R.; Walker,D.; Seiler,P.; Mukherjee,N.; Kercher,L.; McKenzie,P.; Feeroz,M.; Webby,R.J.
EPI1778093	HA	Bangladesh	2019-Jul-11	A/duck/Bangladesh/40524/2019	NA	H5	Import from public-domain	Barman,S.; Turner,J.C.; Hasan,M.; Akhtar,S.; Franks,J.; El-Shesheny,R.; Walker,D.; Seiler,P.; Mukherjee,N.; Kercher,L.; McKenzie,P.; Feeroz,M.; Webby,R.J.
EPI1778028	HA	Bangladesh	2019-Jul-10	A/duck/Bangladesh/40528/2019	NA	H5	Import from public-domain	Barman,S.; Turner,J.C.; Hasan,M.; Akhtar,S.; Franks,J.; El-Shesheny,R.; Walker,D.; Seiler,P.; Mukherjee,N.; Kercher,L.; McKenzie,P.; Feeroz,M.; Webby,R.J.
EPI1778014	HA	Bangladesh	2019-Aug-19	A/duck/Bangladesh/40638/2019	NA	H5	Import from public-domain	Barman,S.; Turner,J.C.; Hasan,M.; Akhtar,S.; Franks,J.; El-Shesheny,R.; Walker,D.; Seiler,P.; Mukherjee,N.; Kercher,L.; McKenzie,P.; Feeroz,M.; Webby,R.J.
EPI1777970	HA	Bangladesh	2019-Feb-18	A/duck/Bangladesh/38287/2019	NA	H5	Import from public-domain	Barman,S.; Turner,J.C.; Hasan,M.; Akhtar,S.; Franks,J.; El-Shesheny,R.; Walker,D.; Seiler,P.; Mukherjee,N.; Kercher,L.; McKenzie,P.; Feeroz,M.; Webby,R.J.
EPI1777925	HA	Bangladesh	2019-Aug-19	A/duck/Bangladesh/40641/2019	NA	H5	Import from public-domain	Barman,S.; Turner,J.C.; Hasan,M.; Akhtar,S.; Franks,J.; El-Shesheny,R.; Walker,D.; Seiler,P.; Mukherjee,N.; Kercher,L.; McKenzie,P.; Feeroz,M.; Webby,R.J.
EPI1777881	HA	Bangladesh	2019-Jul-11	A/chicken/Bangladesh/40446/2019	NA	H5	Import from public-domain	Barman,S.; Turner,J.C.; Hasan,M.; Akhtar,S.; Franks,J.; El-Shesheny,R.; Walker,D.; Seiler,P.; Mukherjee,N.; Kercher,L.; McKenzie,P.; Feeroz,M.; Webby,R.J.
EPI1777837	HA	Bangladesh	2019-Feb-19	A/duck/Bangladesh/38175/2019	NA	H5	Import from public-domain	Barman,S.; Turner,J.C.; Hasan,M.; Akhtar,S.; Franks,J.; El-Shesheny,R.; Walker,D.; Seiler,P.; Mukherjee,N.; Kercher,L.; McKenzie,P.; Feeroz,M.; Webby,R.J.
EPI1620332	HA	Bangladesh	2017-Jun-11	A/quail/Bangladesh/32942/2017	NA	H5	Import from public-domain	Barman,S.; Turner,J.C.; Hasan,M.K.; Akhtar,S.; Franks,J.; El-Shesheny,R.; Walker,D.; Seiler,P.; Friedman,K.; Kercher,L.; Kayali,G.; Jones-Engel,L.; McKenzie,P.; Krauss,S.; Webby,R.J.; Feeroz,M.; Webster,R.G.
EPI1557276	HA	Bangladesh	2017-Dec-25	A/quail/Bangladesh/34292/2017	NA	H5	Import from public-domain	Barman,S.; Turner,J.C.; Hasan,M.K.; Akhtar,S.; Franks,J.; El-Shesheny,R.; Walker,D.; Seiler,P.; Friedman,K.; Kercher,L.; Kayali,G.; Jones-Engel,L.; McKenzie,P.; Krauss,S.; Webby,R.J.; Feeroz,M.M.; Webster,R.G.

EPI1556208	HA	Bangladesh	2017-Dec-25	A/chicken/Bangladesh/34265/2017	NA	H5	Import from public-domain	Barman,S. ; Turner,J.C. ; Hasan,M.K. ; Akhtar,S. ; Franks,J. ; El-Shesheny,R. ; Walker,D. ; Seiler,P. ; Friedman,K. ; Kercher,L. ; Kayali,G. ; Jones-Engel,L. ; McKenzie,P. ; Krauss,S. ; Webby,R.J. ; Feeroz,M.M. ; Webster,R.G.
EPI1556204	HA	Bangladesh	2018-Jan-24	A/chicken/Bangladesh/34725/2018	NA	H5	Import from public-domain	Barman,S. ; Turner,J.C. ; Hasan,M.K. ; Akhtar,S. ; Franks,J. ; El-Shesheny,R. ; Walker,D. ; Seiler,P. ; Friedman,K. ; Kercher,L. ; Kayali,G. ; Jones-Engel,L. ; McKenzie,P. ; Krauss,S. ; Webby,R.J. ; Feeroz,M.M. ; Webster,R.G.
EPI1556197	HA	Bangladesh	2018-Jan-24	A/duck/Bangladesh/34743/2018	NA	H5	Import from public-domain	Barman,S. ; Turner,J.C. ; Hasan,M.K. ; Akhtar,S. ; Franks,J. ; El-Shesheny,R. ; Walker,D. ; Seiler,P. ; Friedman,K. ; Kercher,L. ; Kayali,G. ; Jones-Engel,L. ; McKenzie,P. ; Krauss,S. ; Webby,R.J. ; Feeroz,M.M. ; Webster,R.G.
EPI1556127	HA	Bangladesh	2018-Jan-24	A/duck/Bangladesh/34744/2018	NA	H5	Import from public-domain	Barman,S. ; Turner,J.C. ; Hasan,M.K. ; Akhtar,S. ; Franks,J. ; El-Shesheny,R. ; Walker,D. ; Seiler,P. ; Friedman,K. ; Kercher,L. ; Kayali,G. ; Jones-Engel,L. ; McKenzie,P. ; Krauss,S. ; Webby,R.J. ; Feeroz,M.M. ; Webster,R.G.
EPI1556118	HA	Bangladesh	2018-Jan-24	A/duck/Bangladesh/34745/2018	NA	H5	Import from public-domain	Barman,S. ; Turner,J.C. ; Hasan,M.K. ; Akhtar,S. ; Franks,J. ; El-Shesheny,R. ; Walker,D. ; Seiler,P. ; Friedman,K. ; Kercher,L. ; Kayali,G. ; Jones-Engel,L. ; McKenzie,P. ; Krauss,S. ; Webby,R.J. ; Feeroz,M.M. ; Webster,R.G.
EPI1556097	HA	Bangladesh	2018-Jan-24	A/duck/Bangladesh/34752/2018	NA	H5	Import from public-domain	Barman,S. ; Turner,J.C. ; Hasan,M.K. ; Akhtar,S. ; Franks,J. ; El-Shesheny,R. ; Walker,D. ; Seiler,P. ; Friedman,K. ; Kercher,L. ; Kayali,G. ; Jones-Engel,L. ; McKenzie,P. ; Krauss,S. ; Webby,R.J. ; Feeroz,M.M. ; Webster,R.G.
EPI1556084	HA	Bangladesh	2018-Jan-23	A/duck/Bangladesh/34590/2018	NA	H5	Import from public-domain	Barman,S. ; Turner,J.C. ; Hasan,M.K. ; Akhtar,S. ; Franks,J. ; El-Shesheny,R. ; Walker,D. ; Seiler,P. ; Friedman,K. ; Kercher,L. ; Kayali,G. ; Jones-Engel,L. ; McKenzie,P. ; Krauss,S. ; Webby,R.J. ; Feeroz,M.M. ; Webster,R.G.
EPI1556080	HA	Bangladesh	2017-Nov-28	A/duck/Bangladesh/34100/2017	NA	H5	Import from public-domain	Barman,S. ; Turner,J.C. ; Hasan,M.K. ; Akhtar,S. ; Franks,J. ; El-Shesheny,R. ; Walker,D. ; Seiler,P. ; Friedman,K. ; Kercher,L. ; Kayali,G. ; Jones-Engel,L. ; McKenzie,P. ; Krauss,S. ; Webby,R.J. ; Feeroz,M.M. ; Webster,R.G.
EPI1556076	HA	Bangladesh	2017-Nov-28	A/duck/Bangladesh/34097/2017	NA	H5	Import from public-domain	Barman,S. ; Turner,J.C. ; Hasan,M.K. ; Akhtar,S. ; Franks,J. ; El-Shesheny,R. ; Walker,D. ; Seiler,P. ; Friedman,K. ; Kercher,L. ; Kayali,G. ; Jones-Engel,L. ; McKenzie,P. ; Krauss,S. ; Webby,R.J. ; Feeroz,M.M. ; Webster,R.G.
EPI1556014	HA	Bangladesh	2017-Nov-28	A/duck/Bangladesh/34105/2017	NA	H5	Import from public-domain	Barman,S. ; Turner,J.C. ; Hasan,M.K. ; Akhtar,S. ; Franks,J. ; El-Shesheny,R. ; Walker,D. ; Seiler,P. ; Friedman,K. ; Kercher,L. ; Kayali,G. ; Jones-Engel,L. ; McKenzie,P. ; Krauss,S. ; Webby,R.J. ; Feeroz,M.M. ; Webster,R.G.
EPI1556001	HA	Bangladesh	2018-Jan-24	A/duck/Bangladesh/34749/2018	NA	H5	Import from public-domain	Barman,S. ; Turner,J.C. ; Hasan,M.K. ; Akhtar,S. ; Franks,J. ; El-Shesheny,R. ; Walker,D. ; Seiler,P. ; Friedman,K. ; Kercher,L. ; Kayali,G. ; Jones-Engel,L. ; McKenzie,P. ; Krauss,S. ; Webby,R.J. ; Feeroz,M.M. ; Webster,R.G.
EPI1555992	HA	Bangladesh	2018-Jan-24	A/duck/Bangladesh/34751/2018	NA	H5	Import from public-domain	Barman,S. ; Turner,J.C. ; Hasan,M.K. ; Akhtar,S. ; Franks,J. ; El-Shesheny,R. ; Walker,D. ; Seiler,P. ; Friedman,K. ; Kercher,L. ; Kayali,G. ; Jones-Engel,L. ; McKenzie,P. ; Krauss,S. ; Webby,R.J. ; Feeroz,M.M. ; Webster,R.G.
EPI1555989	HA	Bangladesh	2017-Nov-28	A/duck/Bangladesh/34032/2017	NA	H5	Import from public-domain	Barman,S. ; Turner,J.C. ; Hasan,M.K. ; Akhtar,S. ; Franks,J. ; El-Shesheny,R. ; Walker,D. ; Seiler,P. ; Friedman,K. ; Kercher,L. ; Kayali,G. ; Jones-Engel,L. ; McKenzie,P. ; Krauss,S. ; Webby,R.J. ; Feeroz,M.M. ; Webster,R.G.
EPI1555987	HA	Bangladesh	2017-Nov-28	A/duck/Bangladesh/34036/2017	NA	H5	Import from public-domain	Barman,S. ; Turner,J.C. ; Hasan,M.K. ; Akhtar,S. ; Franks,J. ; El-Shesheny,R. ; Walker,D. ; Seiler,P. ; Friedman,K. ; Kercher,L. ; Kayali,G. ; Jones-Engel,L. ; McKenzie,P. ; Krauss,S. ; Webby,R.J. ; Feeroz,M.M. ; Webster,R.G.
EPI1555983	HA	Bangladesh	2017-Nov-28	A/duck/Bangladesh/34035/2017	NA	H5	Import from public-domain	Barman,S. ; Turner,J.C. ; Hasan,M.K. ; Akhtar,S. ; Franks,J. ; El-Shesheny,R. ; Walker,D. ; Seiler,P. ; Friedman,K. ; Kercher,L. ; Kayali,G. ; Jones-Engel,L. ; McKenzie,P. ; Krauss,S. ; Webby,R.J. ; Feeroz,M.M. ; Webster,R.G.
EPI1555970	HA	Bangladesh	2017-Nov-25	A/duck/Bangladesh/34199/2017	NA	H5	Import from public-domain	Barman,S. ; Turner,J.C. ; Hasan,M.K. ; Akhtar,S. ; Franks,J. ; El-Shesheny,R. ; Walker,D. ; Seiler,P. ; Friedman,K. ; Kercher,L. ; Kayali,G. ; Jones-Engel,L. ; McKenzie,P. ; Krauss,S. ; Webby,R.J. ; Feeroz,M.M. ; Webster,R.G.
EPI1555936	HA	Bangladesh	2017-Nov-28	A/duck/Bangladesh/34104/2017	NA	H5	Import from public-domain	Barman,S. ; Turner,J.C. ; Hasan,M.K. ; Akhtar,S. ; Franks,J. ; El-Shesheny,R. ; Walker,D. ; Seiler,P. ; Friedman,K. ; Kercher,L. ; Kayali,G. ; Jones-Engel,L. ; McKenzie,P. ; Krauss,S. ; Webby,R.J. ; Feeroz,M.M. ; Webster,R.G.
EPI1555931	HA	Bangladesh	2017-Dec-25	A/duck/Bangladesh/34283/2017	NA	H5	Import from public-domain	Barman,S. ; Turner,J.C. ; Hasan,M.K. ; Akhtar,S. ; Franks,J. ; El-Shesheny,R. ; Walker,D. ; Seiler,P. ; Friedman,K. ; Kercher,L. ; Kayali,G. ; Jones-Engel,L. ; McKenzie,P. ; Krauss,S. ; Webby,R.J. ; Feeroz,M.M. ; Webster,R.G.
EPI1555913	HA	Bangladesh	2017-Dec-25	A/duck/Bangladesh/34285/2017	NA	H5	Import from public-domain	Barman,S. ; Turner,J.C. ; Hasan,M.K. ; Akhtar,S. ; Franks,J. ; El-Shesheny,R. ; Walker,D. ; Seiler,P. ; Friedman,K. ; Kercher,L. ; Kayali,G. ; Jones-Engel,L. ; McKenzie,P. ; Krauss,S. ; Webby,R.J. ; Feeroz,M.M. ; Webster,R.G.
EPI1555894	HA	Bangladesh	2017-Nov-28	A/duck/Bangladesh/34102/2017	NA	H5	Import from public-domain	Barman,S. ; Turner,J.C. ; Hasan,M.K. ; Akhtar,S. ; Franks,J. ; El-Shesheny,R. ; Walker,D. ; Seiler,P. ; Friedman,K. ; Kercher,L. ; Kayali,G. ; Jones-Engel,L. ; McKenzie,P. ; Krauss,S. ; Webby,R.J. ; Feeroz,M.M. ; Webster,R.G.
EPI1555876	HA	Bangladesh	2018-Jan-24	A/chicken/Bangladesh/34722/2018	NA	H5	Import from public-domain	Barman,S. ; Turner,J.C. ; Hasan,M.K. ; Akhtar,S. ; Franks,J. ; El-Shesheny,R. ; Walker,D. ; Seiler,P. ; Friedman,K. ; Kercher,L. ; Kayali,G. ; Jones-Engel,L. ; McKenzie,P. ; Krauss,S. ; Webby,R.J. ; Feeroz,M.M. ; Webster,R.G.
EPI1309692	HA	Bangladesh	2017-May-18	A/duck/Bangladesh/32822/2017	NA	H5	Import from public-domain	Barman,S. ; Turner,J.C. ; Hasan,M.K. ; Akhtar,S. ; Franks,J. ; El-Shesheny,R. ; Walker,D. ; Seiler,P. ; Friedman,K. ; Kercher,L. ; Kayali,G. ; Jones-Engel,L. ; McKenzie,P. ; Krauss,S. ; Webby,R.J. ; Feeroz,M. ; Webster,R.G.
EPI1309636	HA	Bangladesh	2017-Aug-17	A/duck/Bangladesh/33415/2017	NA	H5	Import from public-domain	Barman,S. ; Turner,J.C. ; Hasan,M.K. ; Akhtar,S. ; Franks,J. ; El-Shesheny,R. ; Walker,D. ; Seiler,P. ; Friedman,K. ; Kercher,L. ; Kayali,G. ; Jones-Engel,L. ; McKenzie,P. ; Krauss,S. ; Webby,R.J. ; Feeroz,M. ; Webster,R.G.
EPI1309609	HA	Bangladesh	2017-Oct-19	A/duck/Bangladesh/33848/2017	NA	H5	Import from public-domain	Barman,S. ; Turner,J.C. ; Hasan,M.K. ; Akhtar,S. ; Franks,J. ; El-Shesheny,R. ; Walker,D. ; Seiler,P. ; Friedman,K. ; Kercher,L. ; Kayali,G. ; Jones-Engel,L. ; McKenzie,P. ; Krauss,S. ; Webby,R.J. ; Feeroz,M. ; Webster,R.G.
EPI1309601	HA	Bangladesh	2017-Oct-19	A/duck/Bangladesh/33772/2017	NA	H5	Import from public-domain	Barman,S. ; Turner,J.C. ; Hasan,M.K. ; Akhtar,S. ; Franks,J. ; El-Shesheny,R. ; Walker,D. ; Seiler,P. ; Friedman,K. ; Kercher,L. ; Kayali,G. ; Jones-Engel,L. ; McKenzie,P. ; Krauss,S. ; Webby,R.J. ; Feeroz,M. ; Webster,R.G.
EPI1309592	HA	Bangladesh	2017-May-18	A/chicken/Bangladesh/32806/2017	NA	H5	Import from public-domain	Barman,S. ; Turner,J.C. ; Hasan,M.K. ; Akhtar,S. ; Franks,J. ; El-Shesheny,R. ; Walker,D. ; Seiler,P. ; Friedman,K. ; Kercher,L. ; Kayali,G. ; Jones-Engel,L. ; McKenzie,P. ; Krauss,S. ; Webby,R.J. ; Feeroz,M. ; Webster,R.G.
EPI1309572	HA	Bangladesh	2017-Oct-19	A/duck/Bangladesh/33775/2017	NA	H5	Import from public-domain	Barman,S. ; Turner,J.C. ; Hasan,M.K. ; Akhtar,S. ; Franks,J. ; El-Shesheny,R. ; Walker,D. ; Seiler,P. ; Friedman,K. ; Kercher,L. ; Kayali,G. ; Jones-Engel,L. ; McKenzie,P. ; Krauss,S. ; Webby,R.J. ; Feeroz,M. ; Webster,R.G.
EPI1309569	HA	Bangladesh	2017-Oct-19	A/duck/Bangladesh/33841/2017	NA	H5	Import from public-domain	Barman,S. ; Turner,J.C. ; Hasan,M.K. ; Akhtar,S. ; Franks,J. ; El-Shesheny,R. ; Walker,D. ; Seiler,P. ; Friedman,K. ; Kercher,L. ; Kayali,G. ; Jones-Engel,L. ; McKenzie,P. ; Krauss,S. ; Webby,R.J. ; Feeroz,M. ; Webster,R.G.
EPI1309552	HA	Bangladesh	2017-Oct-24	A/duck/Bangladesh/33892/2017	NA	H5	Import from public-domain	Barman,S. ; Turner,J.C. ; Hasan,M.K. ; Akhtar,S. ; Franks,J. ; El-Shesheny,R. ; Walker,D. ; Seiler,P. ; Friedman,K. ; Kercher,L. ; Kayali,G. ; Jones-Engel,L. ; McKenzie,P. ; Krauss,S. ; Webby,R.J. ; Feeroz,M. ; Webster,R.G.
EPI1309490	HA	Bangladesh	2017-May-20	A/chicken/Bangladesh/32747/2017	NA	H5	Import from public-domain	Barman,S. ; Turner,J.C. ; Hasan,M.K. ; Akhtar,S. ; Franks,J. ; El-Shesheny,R. ; Walker,D. ; Seiler,P. ; Friedman,K. ; Kercher,L. ; Kayali,G. ; Jones-Engel,L. ; McKenzie,P. ; Krauss,S. ; Webby,R.J. ; Feeroz,M. ; Webster,R.G.
EPI1309481	HA	Bangladesh	2017-Jun-11	A/duck/Bangladesh/32914/2017	NA	H5	Import from public-domain	Barman,S. ; Turner,J.C. ; Hasan,M.K. ; Akhtar,S. ; Franks,J. ; El-Shesheny,R. ; Walker,D. ; Seiler,P. ; Friedman,K. ; Kercher,L. ; Kayali,G. ; Jones-Engel,L. ; McKenzie,P. ; Krauss,S. ; Webby,R.J. ; Feeroz,M. ; Webster,R.G.
EPI1213888	HA	Bangladesh	2017-Apr-19	A/duck/Bangladesh/32502/2017	NA	H5	Import from public-domain	Barman,S. ; Turner,J.C. ; Hasan,M.K. ; Akhtar,S. ; Franks,J. ; El-Shesheny,R. ; Walker,D. ; Seiler,P. ; Friedman,K. ; Kercher,L. ; Kayali,G. ; Jones-Engel,L. ; McKenzie,P. ; Krauss,S. ; Webby,R.J. ; Feeroz,M.M. ; Webster,R.G.
EPI1213883	HA	Bangladesh	2017-Feb-15	A/duck/Bangladesh/32003/2017	NA	H5	Import from public-domain	Barman,S. ; Turner,J.C. ; Hasan,M.K. ; Akhtar,S. ; Franks,J. ; El-Shesheny,R. ; Walker,D. ; Seiler,P. ; Friedman,K. ; Kercher,L. ; Kayali,G. ; Jones-Engel,L. ; McKenzie,P. ; Krauss,S. ; Webby,R.J. ; Feeroz,M.M. ; Webster,R.G.
EPI1213880	HA	Bangladesh	2017-Feb-15	A/duck/Bangladesh/31998/2017	NA	H5	Import from public-domain	Barman,S. ; Turner,J.C. ; Hasan,M.K. ; Akhtar,S. ; Franks,J. ; El-Shesheny,R. ; Walker,D. ; Seiler,P. ; Friedman,K. ; Kercher,L. ; Kayali,G. ; Jones-Engel,L. ; McKenzie,P. ; Krauss,S. ; Webby,R.J. ; Feeroz,M.M. ; Webster,R.G.
EPI1213876	HA	Bangladesh	2017-Feb-15	A/duck/Bangladesh/31992/2017	NA	H5	Import from public-domain	Barman,S. ; Turner,J.C. ; Hasan,M.K. ; Akhtar,S. ; Franks,J. ; El-Shesheny,R. ; Walker,D. ; Seiler,P. ; Friedman,K. ; Kercher,L. ; Kayali,G. ; Jones-Engel,L. ; McKenzie,P. ; Krauss,S. ; Webby,R.J. ; Feeroz,M.M. ; Webster,R.G.
EPI1213859	HA	Bangladesh	2017-Mar-29	A/duck/Bangladesh/32247/2017	NA	H5	Import from public-domain	Barman,S. ; Turner,J.C. ; Hasan,M.K. ; Akhtar,S. ; Franks,J. ; El-Shesheny,R. ; Walker,D. ; Seiler,P. ; Friedman,K. ; Kercher,L. ; Kayali,G. ; Jones-Engel,L. ; McKenzie,P. ; Krauss,S. ; Webby,R.J. ; Feeroz,M.M. ; Webster,R.G.
EPI1213809	HA	Bangladesh	2017-Feb-15	A/duck/Bangladesh/31993/2017	NA	H5	Import from public-domain	Barman,S. ; Turner,J.C. ; Hasan,M.K. ; Akhtar,S. ; Franks,J. ; El-Shesheny,R. ; Walker,D. ; Seiler,P. ; Friedman,K. ; Kercher,L. ; Kayali,G. ; Jones-Engel,L. ; McKenzie,P. ; Krauss,S. ; Webby,R.J. ; Feeroz,M.M. ; Webster,R.G.
EPI1213807	HA	Bangladesh	2017-Apr-25	A/duck/Bangladesh/32664/2017	NA	H5	Import from public-domain	Barman,S. ; Turner,J.C. ; Hasan,M.K. ; Akhtar,S. ; Franks,J. ; El-Shesheny,R. ; Walker,D. ; Seiler,P. ; Friedman,K. ; Kercher,L. ; Kayali,G. ; Jones-Engel,L. ; McKenzie,P. ; Krauss,S. ; Webby,R.J. ; Feeroz,M.M. ; Webster,R.G.
EPI1099150	HA	Bangladesh	2016-Sep-27	A/duck/Bangladesh/30821/2016	NA	H5	Import from public-domain	Barman,S. ; Turner,J.C. ; Hasan,M.K. ; Akhtar,S. ; Franks,J. ; El-Shesheny,R. ; Walker,D. ; Seiler,P. ; Friedman,K. ; Kercher,L. ; Kayali,G. ; Jones-Engel,L. ; McKenzie,P. ; Krauss,S. ; Webby,R.J. ; Feeroz,M.M. ; Webster,R.G.
EPI1099138	HA	Bangladesh	2016-Oct-24	A/duck/Bangladesh/31097/2016	NA	H5	Import from public-domain	Barman,S. ; Turner,J.C. ; Hasan,M.K. ; Akhtar,S. ; Franks,J. ; El-Shesheny,R. ; Walker,D. ; Seiler,P. ; Friedman,K. ; Kercher,L. ; Kayali,G. ; Jones-Engel,L. ; McKenzie,P. ; Krauss,S. ; Webby,R.J. ; Feeroz,M.M. ; Webster,R.G.
EPI1099126	HA	Bangladesh	2016-Oct-24	A/duck/Bangladesh/31095/2016	NA	H5	Import from public-domain	Barman,S. ; Turner,J.C. ; Hasan,M.K. ; Akhtar,S. ; Franks,J. ; El-Shesheny,R. ; Walker,D. ; Seiler,P. ; Friedman,K. ; Kercher,L. ; Kayali,G. ; Jones-Engel,L. ; McKenzie,P. ; Krauss,S. ; Webby,R.J. ; Feeroz,M.M. ; Webster,R.G.
EPI1099121	HA	Bangladesh	2016-Oct-24	A/duck/Bangladesh/31023/2016	NA	H5	Import from public-domain	Barman,S. ; Turner,J.C. ; Hasan,M.K. ; Akhtar,S. ; Franks,J. ; El-Shesheny,R. ; Walker,D. ; Seiler,P. ; Friedman,K. ; Kercher,L. ; Kayali,G. ; Jones-Engel,L. ; McKenzie,P. ; Krauss,S. ; Webby,R.J. ; Feeroz,M.M. ; Webster,R.G.
EPI1099102	HA	Bangladesh	2016-Sep-27	A/duck/Bangladesh/30817/2016	NA	H5	Import from public-domain	Barman,S. ; Turner,J.C. ; Hasan,M.K. ; Akhtar,S. ; Franks,J. ; El-Shesheny,R. ; Walker,D. ; Seiler,P. ; Friedman,K. ; Kercher,L. ; Kayali,G. ; Jones-Engel,L. ; McKenzie,P. ; Krauss,S. ; Webby,R.J. ; Feeroz,M.M. ; Webster,R.G.
EPI1099041	HA	Bangladesh	2016-Oct-24	A/duck/Bangladesh/31022/2016	NA	H5	Import from public-domain	Barman,S. ; Turner,J.C. ; Hasan,M.K. ; Akhtar,S. ; Franks,J. ; El-Shesheny,R. ; Walker,D. ; Seiler,P. ; Friedman,K. ; Kercher,L. ; Kayali,G. ; Jones-Engel,L. ; McKenzie,P. ; Krauss,S. ; Webby,R.J. ; Feeroz,M.M. ; Webster,R.G.
EPI1099037	HA	Bangladesh	2016-Sep-27	A/duck/Bangladesh/30884/2016	NA	H5	Import from public-domain	Barman,S. ; Turner,J.C. ; Hasan,M.K. ; Akhtar,S. ; Franks,J. ; El-Shesheny,R. ; Walker,D. ; Seiler,P. ; Friedman,K. ; Kercher,L. ; Kayali,G. ; Jones-Engel,L. ; McKenzie,P. ; Krauss,S. ; Webby,R.J. ; Feeroz,M.M. ; Webster,R.G.

EPI1099027	HA	Bangladesh	2016-Sep-27	A/duck/Bangladesh/30818/2016	NA	H5	Import from public-domain	Barman,S. ; Turner,J.C. ; Hasan,M.K. ; Akhtar,S. ; Franks,J. ; El-Shesheny,R. ; Walker,D. ; Seiler,P. ; Friedman,K. ; Kercher,L. ; Kayali,G. ; Jones-Engel,L. ; McKenzie,P. ; Krauss,S. ; Webby,R.J. ; Feeroz,M.M. ; Webster,R.G.
EPI1099019	HA	Bangladesh	2016-Sep-27	A/duck/Bangladesh/30885/2016	NA	H5	Import from public-domain	Barman,S. ; Turner,J.C. ; Hasan,M.K. ; Akhtar,S. ; Franks,J. ; El-Shesheny,R. ; Walker,D. ; Seiler,P. ; Friedman,K. ; Kercher,L. ; Kayali,G. ; Jones-Engel,L. ; McKenzie,P. ; Krauss,S. ; Webby,R.J. ; Feeroz,M.M. ; Webster,R.G.
EPI1098990	HA	Bangladesh	2016-Nov-25	A/duck/Bangladesh/31297/2016	NA	H5	Import from public-domain	Barman,S. ; Turner,J.C. ; Hasan,M.K. ; Akhtar,S. ; Franks,J. ; El-Shesheny,R. ; Walker,D. ; Seiler,P. ; Friedman,K. ; Kercher,L. ; Kayali,G. ; Jones-Engel,L. ; McKenzie,P. ; Krauss,S. ; Webby,R.J. ; Feeroz,M.M. ; Webster,R.G.
EPI1098974	HA	Bangladesh	2016-Sep-27	A/duck/Bangladesh/30892/2016	NA	H5	Import from public-domain	Barman,S. ; Turner,J.C. ; Hasan,M.K. ; Akhtar,S. ; Franks,J. ; El-Shesheny,R. ; Walker,D. ; Seiler,P. ; Friedman,K. ; Kercher,L. ; Kayali,G. ; Jones-Engel,L. ; McKenzie,P. ; Krauss,S. ; Webby,R.J. ; Feeroz,M.M. ; Webster,R.G.
EPI1098959	HA	Bangladesh	2016-Dec-21	A/duck/Bangladesh/31549/2016	NA	H5	Import from public-domain	Barman,S. ; Turner,J.C. ; Hasan,M.K. ; Akhtar,S. ; Franks,J. ; El-Shesheny,R. ; Walker,D. ; Seiler,P. ; Friedman,K. ; Kercher,L. ; Kayali,G. ; Jones-Engel,L. ; McKenzie,P. ; Krauss,S. ; Webby,R.J. ; Feeroz,M.M. ; Webster,R.G.
EPI1098955	HA	Bangladesh	2016-May-27	A/chicken/Bangladesh/30065/2016	NA	H5	Import from public-domain	Barman,S. ; Turner,J.C. ; Hasan,M.K. ; Akhtar,S. ; Franks,J. ; El-Shesheny,R. ; Walker,D. ; Seiler,P. ; Friedman,K. ; Kercher,L. ; Kayali,G. ; Jones-Engel,L. ; McKenzie,P. ; Krauss,S. ; Webby,R.J. ; Feeroz,M.M. ; Webster,R.G.
EPI1098942	HA	Bangladesh	2016-Oct-24	A/duck/Bangladesh/31103/2016	NA	H5	Import from public-domain	Barman,S. ; Turner,J.C. ; Hasan,M.K. ; Akhtar,S. ; Franks,J. ; El-Shesheny,R. ; Walker,D. ; Seiler,P. ; Friedman,K. ; Kercher,L. ; Kayali,G. ; Jones-Engel,L. ; McKenzie,P. ; Krauss,S. ; Webby,R.J. ; Feeroz,M.M. ; Webster,R.G.
EPI1098941	HA	Bangladesh	2016-Aug-21	A/duck/Bangladesh/30682/2016	NA	H5	Import from public-domain	Barman,S. ; Turner,J.C. ; Hasan,M.K. ; Akhtar,S. ; Franks,J. ; El-Shesheny,R. ; Walker,D. ; Seiler,P. ; Friedman,K. ; Kercher,L. ; Kayali,G. ; Jones-Engel,L. ; McKenzie,P. ; Krauss,S. ; Webby,R.J. ; Feeroz,M.M. ; Webster,R.G.
EPI1098909	HA	Bangladesh	2016-Nov-25	A/duck/Bangladesh/31296/2016	NA	H5	Import from public-domain	Barman,S. ; Turner,J.C. ; Hasan,M.K. ; Akhtar,S. ; Franks,J. ; El-Shesheny,R. ; Walker,D. ; Seiler,P. ; Friedman,K. ; Kercher,L. ; Kayali,G. ; Jones-Engel,L. ; McKenzie,P. ; Krauss,S. ; Webby,R.J. ; Feeroz,M.M. ; Webster,R.G.
EPI1098907	HA	Bangladesh	2016-Sep-27	A/duck/Bangladesh/30890/2016	NA	H5	Import from public-domain	Barman,S. ; Turner,J.C. ; Hasan,M.K. ; Akhtar,S. ; Franks,J. ; El-Shesheny,R. ; Walker,D. ; Seiler,P. ; Friedman,K. ; Kercher,L. ; Kayali,G. ; Jones-Engel,L. ; McKenzie,P. ; Krauss,S. ; Webby,R.J. ; Feeroz,M.M. ; Webster,R.G.
EPI1098906	HA	Bangladesh	2016-Nov-25	A/duck/Bangladesh/31294/2016	NA	H5	Import from public-domain	Barman,S. ; Turner,J.C. ; Hasan,M.K. ; Akhtar,S. ; Franks,J. ; El-Shesheny,R. ; Walker,D. ; Seiler,P. ; Friedman,K. ; Kercher,L. ; Kayali,G. ; Jones-Engel,L. ; McKenzie,P. ; Krauss,S. ; Webby,R.J. ; Feeroz,M.M. ; Webster,R.G.
EPI1098888	HA	Bangladesh	2016-Oct-24	A/duck/Bangladesh/31093/2016	NA	H5	Import from public-domain	Barman,S. ; Turner,J.C. ; Hasan,M.K. ; Akhtar,S. ; Franks,J. ; El-Shesheny,R. ; Walker,D. ; Seiler,P. ; Friedman,K. ; Kercher,L. ; Kayali,G. ; Jones-Engel,L. ; McKenzie,P. ; Krauss,S. ; Webby,R.J. ; Feeroz,M.M. ; Webster,R.G.
EPI1098882	HA	Bangladesh	2016-Oct-24	A/duck/Bangladesh/31028/2016	NA	H5	Import from public-domain	Barman,S. ; Turner,J.C. ; Hasan,M.K. ; Akhtar,S. ; Franks,J. ; El-Shesheny,R. ; Walker,D. ; Seiler,P. ; Friedman,K. ; Kercher,L. ; Kayali,G. ; Jones-Engel,L. ; McKenzie,P. ; Krauss,S. ; Webby,R.J. ; Feeroz,M.M. ; Webster,R.G.
EPI1098879	HA	Bangladesh	2016-May-26	A/duck/Bangladesh/30057/2016	NA	H5	Import from public-domain	Barman,S. ; Turner,J.C. ; Hasan,M.K. ; Akhtar,S. ; Franks,J. ; El-Shesheny,R. ; Walker,D. ; Seiler,P. ; Friedman,K. ; Kercher,L. ; Kayali,G. ; Jones-Engel,L. ; McKenzie,P. ; Krauss,S. ; Webby,R.J. ; Feeroz,M.M. ; Webster,R.G.
EPI1098877	HA	Bangladesh	2016-Oct-24	A/duck/Bangladesh/31094/2016	NA	H5	Import from public-domain	Barman,S. ; Turner,J.C. ; Hasan,M.K. ; Akhtar,S. ; Franks,J. ; El-Shesheny,R. ; Walker,D. ; Seiler,P. ; Friedman,K. ; Kercher,L. ; Kayali,G. ; Jones-Engel,L. ; McKenzie,P. ; Krauss,S. ; Webby,R.J. ; Feeroz,M.M. ; Webster,R.G.
EPI1098861	HA	Bangladesh	2016-Sep-27	A/duck/Bangladesh/30815/2016	NA	H5	Import from public-domain	Barman,S. ; Turner,J.C. ; Hasan,M.K. ; Akhtar,S. ; Franks,J. ; El-Shesheny,R. ; Walker,D. ; Seiler,P. ; Friedman,K. ; Kercher,L. ; Kayali,G. ; Jones-Engel,L. ; McKenzie,P. ; Krauss,S. ; Webby,R.J. ; Feeroz,M.M. ; Webster,R.G.
EPI1098828	HA	Bangladesh	2016-Nov-25	A/duck/Bangladesh/31295/2016	NA	H5	Import from public-domain	Barman,S. ; Turner,J.C. ; Hasan,M.K. ; Akhtar,S. ; Franks,J. ; El-Shesheny,R. ; Walker,D. ; Seiler,P. ; Friedman,K. ; Kercher,L. ; Kayali,G. ; Jones-Engel,L. ; McKenzie,P. ; Krauss,S. ; Webby,R.J. ; Feeroz,M.M. ; Webster,R.G.
EPI1098827	HA	Bangladesh	2016-Nov-25	A/chicken/Bangladesh/31269/2016	NA	H5	Import from public-domain	Barman,S. ; Turner,J.C. ; Hasan,M.K. ; Akhtar,S. ; Franks,J. ; El-Shesheny,R. ; Walker,D. ; Seiler,P. ; Friedman,K. ; Kercher,L. ; Kayali,G. ; Jones-Engel,L. ; McKenzie,P. ; Krauss,S. ; Webby,R.J. ; Feeroz,M.M. ; Webster,R.G.
EPI1098823	HA	Bangladesh	2016-Nov-25	A/duck/Bangladesh/31299/2016	NA	H5	Import from public-domain	Barman,S. ; Turner,J.C. ; Hasan,M.K. ; Akhtar,S. ; Franks,J. ; El-Shesheny,R. ; Walker,D. ; Seiler,P. ; Friedman,K. ; Kercher,L. ; Kayali,G. ; Jones-Engel,L. ; McKenzie,P. ; Krauss,S. ; Webby,R.J. ; Feeroz,M.M. ; Webster,R.G.
EPI1098810	HA	Bangladesh	2016-Oct-24	A/duck/Bangladesh/31091/2016	NA	H5	Import from public-domain	Barman,S. ; Turner,J.C. ; Hasan,M.K. ; Akhtar,S. ; Franks,J. ; El-Shesheny,R. ; Walker,D. ; Seiler,P. ; Friedman,K. ; Kercher,L. ; Kayali,G. ; Jones-Engel,L. ; McKenzie,P. ; Krauss,S. ; Webby,R.J. ; Feeroz,M.M. ; Webster,R.G.
EPI1098802	HA	Bangladesh	2016-Oct-24	A/duck/Bangladesh/31098/2016	NA	H5	Import from public-domain	Barman,S. ; Turner,J.C. ; Hasan,M.K. ; Akhtar,S. ; Franks,J. ; El-Shesheny,R. ; Walker,D. ; Seiler,P. ; Friedman,K. ; Kercher,L. ; Kayali,G. ; Jones-Engel,L. ; McKenzie,P. ; Krauss,S. ; Webby,R.J. ; Feeroz,M.M. ; Webster,R.G.
EPI1098772	HA	Bangladesh	2016-Oct-24	A/duck/Bangladesh/31096/2016	NA	H5	Import from public-domain	Barman,S. ; Turner,J.C. ; Hasan,M.K. ; Akhtar,S. ; Franks,J. ; El-Shesheny,R. ; Walker,D. ; Seiler,P. ; Friedman,K. ; Kercher,L. ; Kayali,G. ; Jones-Engel,L. ; McKenzie,P. ; Krauss,S. ; Webby,R.J. ; Feeroz,M.M. ; Webster,R.G.
EPI965528	HA	Bangladesh	2015-Jul-10	A/duck/Bangladesh/25887/2015	NA	H5	Import from public-domain	Barman,S. ; Marinova-Petkova,A. ; Hasan,M.K. ; Akhtar,S. ; Turner,J.C. ; Franks,J. ; Walker,D. ; Seiler,P. ; Friedman,K. ; Kercher,L. ; Kayali,G. ; Jones-Engel,L. ; McKenzie,P. ; Krauss,S. ; Webby,R.J. ; Feeroz,M.M. ; Webster,R.G.
EPI965515	HA	Bangladesh	2015-Mar-22	A/duck/Bangladesh/24958/2015	NA	H5	Import from public-domain	Marinova-Petkova,A. ; Barman,S. ; Hasan,M.K. ; Akhtar,S. ; Turner,J.C. ; Franks,J. ; Walker,D. ; Seiler,P. ; Friedman,K. ; Kercher,L. ; Kayali,G. ; Jones-Engel,L. ; McKenzie,P. ; Krauss,S. ; Webby,R.J. ; Feeroz,M.M. ; Webster,R.G.
EPI965514	HA	Bangladesh	2015-Mar-22	A/chicken/Bangladesh/24944/2015	NA	H5	Import from public-domain	Marinova-Petkova,A. ; Barman,S. ; Hasan,M.K. ; Akhtar,S. ; Turner,J.C. ; Franks,J. ; Walker,D. ; Seiler,P. ; Friedman,K. ; Kercher,L. ; Kayali,G. ; Jones-Engel,L. ; McKenzie,P. ; Krauss,S. ; Webby,R.J. ; Feeroz,M.M. ; Webster,R.G.
EPI965510	HA	Bangladesh	2015-Dec-23	A/duck/Bangladesh/27892/2015	NA	H5	Import from public-domain	Barman,S. ; Marinova-Petkova,A. ; Hasan,M.K. ; Akhtar,S. ; Turner,J.C. ; Franks,J. ; Walker,D. ; Seiler,P. ; Friedman,K. ; Kercher,L. ; Kayali,G. ; Jones-Engel,L. ; McKenzie,P. ; Krauss,S. ; Webby,R.J. ; Feeroz,M.M. ; Webster,R.G.
EPI965483	HA	Bangladesh	2015-Sep-12	A/duck/Bangladesh/26188/2015	NA	H5	Import from public-domain	Barman,S. ; Marinova-Petkova,A. ; Hasan,M.K. ; Akhtar,S. ; Turner,J.C. ; Franks,J. ; Walker,D. ; Seiler,P. ; Friedman,K. ; Kercher,L. ; Kayali,G. ; Jones-Engel,L. ; McKenzie,P. ; Krauss,S. ; Webby,R.J. ; Feeroz,M.M. ; Webster,R.G.
EPI965468	HA	Bangladesh	2015-Sep-12	A/duck/Bangladesh/26251/2015	NA	H5	Import from public-domain	Barman,S. ; Marinova-Petkova,A. ; Hasan,M.K. ; Akhtar,S. ; Turner,J.C. ; Franks,J. ; Walker,D. ; Seiler,P. ; Friedman,K. ; Kercher,L. ; Kayali,G. ; Jones-Engel,L. ; McKenzie,P. ; Krauss,S. ; Webby,R.J. ; Feeroz,M.M. ; Webster,R.G.
EPI965413	HA	Bangladesh	2015-Dec-23	A/duck/Bangladesh/27820/2015	NA	H5	Import from public-domain	Barman,S. ; Marinova-Petkova,A. ; Hasan,M.K. ; Akhtar,S. ; Turner,J.C. ; Franks,J. ; Walker,D. ; Seiler,P. ; Friedman,K. ; Kercher,L. ; Kayali,G. ; Jones-Engel,L. ; McKenzie,P. ; Krauss,S. ; Webby,R.J. ; Feeroz,M.M. ; Webster,R.G.
EPI965411	HA	Bangladesh	2015-Jun-26	A/duck/Bangladesh/25683/2015	NA	H5	Import from public-domain	Barman,S. ; Marinova-Petkova,A. ; Hasan,M.K. ; Akhtar,S. ; Turner,J.C. ; Franks,J. ; Walker,D. ; Seiler,P. ; Friedman,K. ; Kercher,L. ; Kayali,G. ; Jones-Engel,L. ; McKenzie,P. ; Krauss,S. ; Webby,R.J. ; Feeroz,M.M. ; Webster,R.G.
EPI965403	HA	Bangladesh	2016-Feb-04	A/duck/Bangladesh/28250/2016	NA	H5	Import from public-domain	Barman,S. ; Marinova-Petkova,A. ; Hasan,M.K. ; Akhtar,S. ; Turner,J.C. ; Franks,J. ; Walker,D. ; Seiler,P. ; Friedman,K. ; Kercher,L. ; Kayali,G. ; Jones-Engel,L. ; McKenzie,P. ; Krauss,S. ; Webby,R.J. ; Feeroz,M.M. ; Webster,R.G.
EPI965386	HA	Bangladesh	2015-Feb-25	A/duck/Bangladesh/24482/2015	NA	H5	Import from public-domain	Marinova-Petkova,A. ; Barman,S. ; Hasan,M.K. ; Akhtar,S. ; Turner,J.C. ; Franks,J. ; Walker,D. ; Seiler,P. ; Friedman,K. ; Kercher,L. ; Kayali,G. ; Jones-Engel,L. ; McKenzie,P. ; Krauss,S. ; Webby,R.J. ; Feeroz,M.M. ; Webster,R.G.
EPI965354	HA	Bangladesh	2015-Aug-25	A/duck/Bangladesh/26042/2015	NA	H5	Import from public-domain	Barman,S. ; Marinova-Petkova,A. ; Hasan,M.K. ; Akhtar,S. ; Turner,J.C. ; Franks,J. ; Walker,D. ; Seiler,P. ; Friedman,K. ; Kercher,L. ; Kayali,G. ; Jones-Engel,L. ; McKenzie,P. ; Krauss,S. ; Webby,R.J. ; Feeroz,M.M. ; Webster,R.G.
EPI965301	HA	Bangladesh	2015-Jul-11	A/duck/Bangladesh/25845/2015	NA	H5	Import from public-domain	Marinova-Petkova,A. ; Barman,S. ; Hasan,M.K. ; Akhtar,S. ; Turner,J.C. ; Franks,J. ; Walker,D. ; Seiler,P. ; Friedman,K. ; Kercher,L. ; Kayali,G. ; Jones-Engel,L. ; McKenzie,P. ; Krauss,S. ; Webby,R.J. ; Feeroz,M.M. ; Webster,R.G.
EPI965289	HA	Bangladesh	2015-Mar-22	A/duck/Bangladesh/24915/2015	NA	H5	Import from public-domain	Marinova-Petkova,A. ; Barman,S. ; Hasan,M.K. ; Akhtar,S. ; Turner,J.C. ; Franks,J. ; Walker,D. ; Seiler,P. ; Friedman,K. ; Kercher,L. ; Kayali,G. ; Jones-Engel,L. ; McKenzie,P. ; Krauss,S. ; Webby,R.J. ; Feeroz,M.M. ; Webster,R.G.
EPI965249	HA	Bangladesh	2015-Apr-20	A/goose/Bangladesh/25169/2015	NA	H5	Import from public-domain	Marinova-Petkova,A. ; Barman,S. ; Hasan,M.K. ; Akhtar,S. ; Turner,J.C. ; Franks,J. ; Walker,D. ; Seiler,P. ; Friedman,K. ; Kercher,L. ; Kayali,G. ; Jones-Engel,L. ; McKenzie,P. ; Krauss,S. ; Webby,R.J. ; Feeroz,M.M. ; Webster,R.G.
EPI965248	HA	Bangladesh	2015-Sep-12	A/duck/Bangladesh/26182/2015	NA	H5	Import from public-domain	Barman,S. ; Marinova-Petkova,A. ; Hasan,M.K. ; Akhtar,S. ; Turner,J.C. ; Franks,J. ; Walker,D. ; Seiler,P. ; Friedman,K. ; Kercher,L. ; Kayali,G. ; Jones-Engel,L. ; McKenzie,P. ; Krauss,S. ; Webby,R.J. ; Feeroz,M.M. ; Webster,R.G.
EPI965238	HA	Bangladesh	2015-Jul-10	A/duck/Bangladesh/25893/2015	NA	H5	Import from public-domain	Marinova-Petkova,A. ; Barman,S. ; Hasan,M.K. ; Akhtar,S. ; Turner,J.C. ; Franks,J. ; Walker,D. ; Seiler,P. ; Friedman,K. ; Kercher,L. ; Kayali,G. ; Jones-Engel,L. ; McKenzie,P. ; Krauss,S. ; Webby,R.J. ; Feeroz,M.M. ; Webster,R.G.
EPI965234	HA	Bangladesh	2016-Feb-29	A/duck/Bangladesh/28389/2016	NA	H5	Import from public-domain	Barman,S. ; Marinova-Petkova,A. ; Hasan,M.K. ; Akhtar,S. ; Turner,J.C. ; Franks,J. ; Walker,D. ; Seiler,P. ; Friedman,K. ; Kercher,L. ; Kayali,G. ; Jones-Engel,L. ; McKenzie,P. ; Krauss,S. ; Webby,R.J. ; Feeroz,M.M. ; Webster,R.G.
EPI965188	HA	Bangladesh	2015-Feb-25	A/duck/Bangladesh/24483/2015	NA	H5	Import from public-domain	Marinova-Petkova,A. ; Barman,S. ; Hasan,M.K. ; Akhtar,S. ; Turner,J.C. ; Franks,J. ; Walker,D. ; Seiler,P. ; Friedman,K. ; Kercher,L. ; Kayali,G. ; Jones-Engel,L. ; McKenzie,P. ; Krauss,S. ; Webby,R.J. ; Feeroz,M.M. ; Webster,R.G.
EPI965185	HA	Bangladesh	2015-Jul-10	A/duck/Bangladesh/25890/2015	NA	H5	Import from public-domain	Marinova-Petkova,A. ; Barman,S. ; Hasan,M.K. ; Akhtar,S. ; Turner,J.C. ; Franks,J. ; Walker,D. ; Seiler,P. ; Friedman,K. ; Kercher,L. ; Kayali,G. ; Jones-Engel,L. ; McKenzie,P. ; Krauss,S. ; Webby,R.J. ; Feeroz,M.M. ; Webster,R.G.
EPI965071	HA	Bangladesh	2014-Mar-18	A/chicken/Bangladesh/22159/2014	NA	H5	Import from public-domain	Marinova-Petkova,A. ; Shanmuganatham,K. ; Feeroz,M.M. ; Jones-Engel,L. ; Hasan,M.K. ; Akhtar,S. ; Turner,J. ; Walker,D. ; Seiler,P. ; Franks,J. ; McKenzie,P. ; Krauss,S. ; Webby,R.J. ; Webster,R.G. ; Hasan,K. ; Seiler,J.
EPI965069	HA	Bangladesh	2014-Apr-12	A/duck/Bangladesh/22399/2014	NA	H5	Import from public-domain	Marinova-Petkova,A. ; Shanmuganatham,K. ; Feeroz,M.M. ; Jones-Engel,L. ; Hasan,M.K. ; Akhtar,S. ; Turner,J. ; Walker,D. ; Seiler,P. ; Franks,J. ; McKenzie,P. ; Krauss,S. ; Webby,R.J. ; Webster,R.G. ; Hasan,K. ; Seiler,J.
EPI965063	HA	Bangladesh	2014-Nov-16	A/duck/Bangladesh/23858/2014	NA	H5	Import from public-domain	Marinova-Petkova,A. ; Shanmuganatham,K. ; Feeroz,M.M. ; Jones-Engel,L. ; Hasan,M.K. ; Akhtar,S. ; Turner,J. ; Walker,D. ; Seiler,P. ; Franks,J. ; McKenzie,P. ; Krauss,S. ; Webby,R.J. ; Webster,R.G. ; Hasan,K. ; Seiler,J.
EPI965056	HA	Bangladesh	2014-Jun-22	A/duck/Bangladesh/22806/2014	NA	H5	Import from public-domain	Marinova-Petkova,A. ; Shanmuganatham,K. ; Feeroz,M.M. ; Jones-Engel,L. ; Hasan,M.K. ; Akhtar,S. ; Turner,J. ; Walker,D. ; Seiler,P. ; Franks,J. ; McKenzie,P. ; Krauss,S. ; Webby,R.J. ; Webster,R.G. ; Hasan,K. ; Seiler,J.

EPI965052	HA	Bangladesh	2014-Apr-15	A/chicken/Bangladesh/22482/2014	NA	H5	Import from public-domain	Marinova-Petkova,A.; Shanmuganatham,K.; Feeroz,M.M.; Jones-Engel,L.; Hasan,M.K.; Akhtar,S.; Turner,J.; Walker,D.; Seiler,P.; Franks,J.; McKenzie,P.; Krauss,S.; Webby,R.J.; Webster,R.G.; Hasan,K.; Seiler,J.
EPI965047	HA	Bangladesh	2014-Jul-17	A/duck/Bangladesh/22996/2014	NA	H5	Import from public-domain	Marinova-Petkova,A.; Shanmuganatham,K.; Feeroz,M.M.; Jones-Engel,L.; Hasan,M.K.; Akhtar,S.; Turner,J.; Walker,D.; Seiler,P.; Franks,J.; McKenzie,P.; Krauss,S.; Webby,R.J.; Webster,R.G.; Hasan,K.; Seiler,J.
EPI965046	HA	Bangladesh	2014-Oct-18	A/duck/Bangladesh/23659/2014	NA	H5	Import from public-domain	Marinova-Petkova,A.; Shanmuganatham,K.; Feeroz,M.M.; Jones-Engel,L.; Hasan,M.K.; Akhtar,S.; Turner,J.; Walker,D.; Seiler,P.; Franks,J.; McKenzie,P.; Krauss,S.; Webby,R.J.; Webster,R.G.; Hasan,K.; Seiler,J.
EPI965032	HA	Bangladesh	2014-Oct-18	A/duck/Bangladesh/23655/2014	NA	H5	Import from public-domain	Marinova-Petkova,A.; Shanmuganatham,K.; Feeroz,M.M.; Jones-Engel,L.; Hasan,M.K.; Akhtar,S.; Turner,J.; Walker,D.; Seiler,P.; Franks,J.; McKenzie,P.; Krauss,S.; Webby,R.J.; Webster,R.G.; Hasan,K.; Seiler,J.
EPI965024	HA	Bangladesh	2014-Dec-29	A/duck/Bangladesh/24044/2014	NA	H5	Import from public-domain	Marinova-Petkova,A.; Shanmuganatham,K.; Feeroz,M.M.; Jones-Engel,L.; Hasan,M.K.; Akhtar,S.; Turner,J.; Walker,D.; Seiler,P.; Franks,J.; McKenzie,P.; Krauss,S.; Webby,R.J.; Webster,R.G.; Hasan,K.; Seiler,J.
EPI965022	HA	Bangladesh	2014-Jul-17	A/duck/Bangladesh/22991/2014	NA	H5	Import from public-domain	Marinova-Petkova,A.; Shanmuganatham,K.; Feeroz,M.M.; Jones-Engel,L.; Hasan,M.K.; Akhtar,S.; Turner,J.; Walker,D.; Seiler,P.; Franks,J.; McKenzie,P.; Krauss,S.; Webby,R.J.; Webster,R.G.; Hasan,K.; Seiler,J.
EPI964995	HA	Bangladesh	2014-Aug-17	A/duck/Bangladesh/23163/2014	NA	H5	Import from public-domain	Marinova-Petkova,A.; Shanmuganatham,K.; Feeroz,M.M.; Jones-Engel,L.; Hasan,M.K.; Akhtar,S.; Turner,J.; Walker,D.; Seiler,P.; Franks,J.; McKenzie,P.; Krauss,S.; Webby,R.J.; Webster,R.G.; Hasan,K.; Seiler,J.
EPI964990	HA	Bangladesh	2014-Oct-18	A/duck/Bangladesh/23656/2014	NA	H5	Import from public-domain	Marinova-Petkova,A.; Shanmuganatham,K.; Feeroz,M.M.; Jones-Engel,L.; Hasan,M.K.; Akhtar,S.; Turner,J.; Walker,D.; Seiler,P.; Franks,J.; McKenzie,P.; Krauss,S.; Webby,R.J.; Webster,R.G.; Hasan,K.; Seiler,J.
EPI964969	HA	Bangladesh	2014-Oct-18	A/duck/Bangladesh/23660/2014	NA	H5	Import from public-domain	Marinova-Petkova,A.; Shanmuganatham,K.; Feeroz,M.M.; Jones-Engel,L.; Hasan,M.K.; Akhtar,S.; Turner,J.; Walker,D.; Seiler,P.; Franks,J.; McKenzie,P.; Krauss,S.; Webby,R.J.; Webster,R.G.; Hasan,K.; Seiler,J.
EPI964937	HA	Bangladesh	2014-Jun-22	A/duck/Bangladesh/22811/2014	NA	H5	Import from public-domain	Marinova-Petkova,A.; Shanmuganatham,K.; Feeroz,M.M.; Jones-Engel,L.; Hasan,M.K.; Akhtar,S.; Turner,J.; Walker,D.; Seiler,P.; Franks,J.; McKenzie,P.; Krauss,S.; Webby,R.J.; Webster,R.G.; Hasan,K.; Seiler,J.
EPI964935	HA	Bangladesh	2014-Dec-29	A/chicken/Bangladesh/23974/2014	NA	H5	Import from public-domain	Marinova-Petkova,A.; Shanmuganatham,K.; Feeroz,M.M.; Jones-Engel,L.; Hasan,M.K.; Akhtar,S.; Turner,J.; Walker,D.; Seiler,P.; Franks,J.; McKenzie,P.; Krauss,S.; Webby,R.J.; Webster,R.G.; Hasan,K.; Seiler,J.
EPI964933	HA	Bangladesh	2014-Apr-15	A/chicken/Bangladesh/22478/2014	NA	H5	Import from public-domain	Marinova-Petkova,A.; Shanmuganatham,K.; Feeroz,M.M.; Jones-Engel,L.; Hasan,M.K.; Akhtar,S.; Turner,J.; Walker,D.; Seiler,P.; Franks,J.; McKenzie,P.; Krauss,S.; Webby,R.J.; Webster,R.G.; Hasan,K.; Seiler,J.
EPI510370	HA	Bangladesh	2012-Jan-18	A/chicken/Bangladesh/15210/2012	NA	H5	Import from public-domain	Marinova-Petkova,A.; Feeroz,M.M.; Alam,S.M.R.; Hasan,M.K.; Akhtar,S.; Jones-Engel,L.; Walker,D.; McClenaghan,L.; Rubrum,A.; Franks,J.; Seiler,P.; Jeevan,T.; McKenzie,P.; Krauss,S.; Webby,R.J.; Webster,R.G.
