# Supplementary material for: H9N2 avian influenza virus dispersal along Bangladeshi poultry trading networks
Source: Virus Evol. 2023 Feb 25;9(1):vead014. doi: 10.1093/ve/vead014 (PMC10032359; doi:10.1093/ve/vead014)
Supplement: vead014_Supp [file vead014_supp.zip › TableS3.pdf]

IRD acknowledgement table

Collector Institution	Collection Date	Country	State / Province	City / Local	Host Species	Host Common Name	Subtype	Flu Test Status	Type	Strain Name	Age	Health
Jahangirnagar University-CEIRS2	01/23/2015	Bangladesh	Dhaka	Changkharpul	Coturnix sp.	Quail	H9	Positive	A	A/quail/Bangladesh/24225/2015(H9N2)	Undetermined	Healthy
Jahangirnagar University-CEIRS2	12/28/2016	Bangladesh	Dhaka	Savar Bazar, Savar	Gallus gallus domesticus	Domestic Chicken	H9	Positive	A	A/chicken/Bangladesh/31624/2016(H9N2)	Hatch Year	Healthy
Jahangirnagar University, Bangladesh	08/14/2011	Bangladesh	Dhaka	Katabon, Dhaka	Coturnix sp.	Quail	H9	Positive	A	A/environment/Bangladesh/12116/2011(H9N2)	Undetermined	Healthy
Jahangirnagar University-CEIRS2	04/19/2017	Bangladesh	Dhaka	Changkharpul, Dhaka	Coturnix sp.	Quail	H9	Positive	A	A/quail/Bangladesh/32525/2017(H9N2)	Hatch Year	Healthy
Jahangirnagar University-CEIRS2	12/18/2018	Bangladesh	Dhaka	Mohammadpur, Dhaka	Gallus gallus domesticus	Domestic Chicken	H9	Positive	A	A/chicken/Bangladesh/35959/2018(H9N2)	Hatch Year	Healthy
Jahangirnagar University-CEIRS2	12/25/2017	Bangladesh	Dhaka	Dhaka	Gallus gallus domesticus	Domestic Chicken	H9	Positive	A	A/environment/Bangladesh/34270/2017(H9N2)	Hatch Year	Healthy
Jahangirnagar University, Bangladesh	02/19/2011	Bangladesh	Dhaka	Karwan Bazaar, Dhaka	Gallus gallus domesticus	Domestic Chicken	H9	Positive	A	A/chicken/Bangladesh/10401/2011(H9N2),A/chicken/Bangladesh/10402/2011(H9N2)	Undetermined	Healthy
Jahangirnagar University, Bangladesh	07/08/2013	Bangladesh	Dhaka	Changkharpul	Coturnix sp.	Quail	H9	Positive	A	A/environment/Bangladesh/20199/2013(H9N2)	Undetermined	Healthy
Jahangirnagar University, Bangladesh	11/15/2010	Bangladesh	Dhaka	Katabon, Dhaka	Psittacidae	Parrot	H9	Positive	A	A/environment/Bangladesh/9306/2010(H9N2)	Undetermined	Healthy
Jahangirnagar University-CEIRS2	08/25/2015	Bangladesh	Dhaka	Kaptan Bazar, Dhaka	Gallus gallus domesticus	Domestic Chicken	H9	Positive	A	A/environment/Bangladesh/25969/2015(H9N2)	Undetermined	Healthy
Jahangirnagar University-CEIRS2	06/11/2017	Bangladesh	Dhaka	Changkharpul, Dhaka	Coturnix sp.	Quail	H9	Positive	A	A/quail/Bangladesh/32935/2017(H9N2)	Hatch Year	Healthy
Jahangirnagar University, Bangladesh	11/24/2011	Bangladesh	Dhaka	Karwan Bazaar, Dhaka,Dhaka	Gallus gallus domesticus	Domestic Chicken	H9	Positive	A	A/chicken/Bangladesh/13916/2011(H9N2)	Undetermined	Healthy
Jahangirnagar University, Bangladesh	08/14/2011	Bangladesh	Dhaka	Katabon, Dhaka	Coturnix sp.	Quail	H9	Positive	A	A/environment/Bangladesh/12119/2011(H9N2)	Undetermined	Healthy
Jahangirnagar University-CEIRS2	08/27/2015	Bangladesh	Dhaka	Savar Bazar, Savar	Gallus gallus domesticus	Domestic Chicken	H9	Positive	A	A/chicken/Bangladesh/26120/2015(H9N2)	Undetermined	Healthy
Jahangirnagar University-CEIRS2	09/13/2019	Bangladesh	Dhaka	Changkharpul, Dhaka	Coturnix sp.	Quail	H9	Positive	A	A/quail/Bangladesh/40847/2019(H9N2)	Hatch Year	Healthy
Jahangirnagar University, Bangladesh	08/14/2011	Bangladesh	Dhaka	Karwan Bazaar, Dhaka	Columba livia	Rock Pigeon	H9	Positive	A	A/environment/Bangladesh/12068/2011(H9N2)	Undetermined	Healthy
Jahangirnagar University-CEIRS2	09/13/2019	Bangladesh	Dhaka	Mohammadpur, Dhaka	Gallus gallus domesticus	Domestic Chicken	H9	Positive	A	A/chicken/Bangladesh/40876/2019(H9N2)	Hatch Year	Healthy
Jahangirnagar University-CEIRS2	03/22/2015	Bangladesh	Dhaka	Changkharpul	Coturnix sp.	Quail	H9	Positive	A	A/quail/Bangladesh/24922/2015(H9N2)	Undetermined	Healthy
Jahangirnagar University, Bangladesh	02/19/2011	Bangladesh	Dhaka	Katabon, Dhaka	Coturnix sp.	Quail	H9	Positive	A	A/environment/Bangladesh/10316/2011(H9N2)	Undetermined	Healthy
Jahangirnagar University-CEIRS2	11/28/2017	Bangladesh	Dhaka	Dhaka	Gallus gallus domesticus	Domestic Chicken	H9	Positive	A	A/environment/Bangladesh/34022/2017(H9N2)	Hatch Year	Healthy
Jahangirnagar University-CEIRS2	07/10/2019	Bangladesh	Dhaka	Savar Bazar, Dhaka	Gallus gallus domesticus	Domestic Chicken	H9	Positive	A	A/chicken/Bangladesh/40498/2019(H9N2)	Hatch Year	Healthy
Jahangirnagar University-CEIRS2	04/26/2018	Bangladesh	Dhaka	Mohammadpur, Dhaka	Gallus gallus domesticus	Domestic Chicken	H9	Positive	A	A/chicken/Bangladesh/35278/2018(H9N2)	Hatch Year	Healthy
Jahangirnagar University-CEIRS2	08/27/2015	Bangladesh	Dhaka	Savar Bazar, Savar	Gallus gallus domesticus	Domestic Chicken	H9	Positive	A	A/chicken/Bangladesh/26102/2015(H9N2)	Undetermined	Healthy
Jahangirnagar University-CEIRS2	09/14/2019	Bangladesh	Dhaka	Savar Bazar, Dhaka	Gallus gallus domesticus	Domestic Chicken	H9	Positive	A	A/chicken/Bangladesh/40926/2019(H9N2)	Hatch Year	Healthy
Jahangirnagar University-CEIRS2	10/19/2014	Bangladesh	Dhaka	Savar Bazar, Savar	Gallus gallus domesticus	Domestic Chicken	H9	Positive	A	A/chicken/Bangladesh/23740/2014(H9N2)	Undetermined	Healthy
Jahangirnagar University-CEIRS2	09/12/2015	Bangladesh	Dhaka	Changkharpul	Coturnix sp.	Quail	H9	Positive	A	A/environment/Bangladesh/26218/2015(H9N2)	Hatch Year	Healthy
Jahangirnagar University-CEIRS2	08/25/2015	Bangladesh	Dhaka	Kaptan Bazar, Dhaka	Gallus gallus domesticus	Domestic Chicken	H9	Positive	A	A/chicken/Bangladesh/25945/2015(H9N2)	Undetermined	Healthy
Jahangirnagar University, Bangladesh	07/08/2013	Bangladesh	Dhaka	Changkharpul	Coturnix sp.	Quail	H9	Positive	A	A/quail/Bangladesh/20177/2013(H9N2)	Undetermined	Healthy
Jahangirnagar University-CEIRS2	12/25/2017	Bangladesh	Dhaka	Dhaka	Gallus gallus domesticus	Domestic Chicken	H9	Positive	A	A/chicken/Bangladesh/34322/2017(H9N2)	Hatch Year	Healthy
Jahangirnagar University-CEIRS2	12/23/2015	Bangladesh	Dhaka	Changkharpul	Coturnix sp.	Quail	H9	Positive	A	A/quail/Bangladesh/27835/2015(H9N2)	Hatch Year	Healthy
Jahangirnagar University-CEIRS2	01/23/2018	Bangladesh	Dhaka	Mohammadpur, Dhaka	Gallus gallus domesticus	Domestic Chicken	H9	Positive	A	A/chicken/Bangladesh/34637/2018(H9N2)	Adult	Healthy
Jahangirnagar University, Bangladesh	12/27/2013	Bangladesh	Dhaka	Changkharpul	Coturnix sp.	Quail	H9	Positive	A	A/quail/Bangladesh/21483/2013(H9N2)	Undetermined	Healthy
Jahangirnagar University-CEIRS2	06/11/2017	Bangladesh	Dhaka	Mohammadpur, Dhaka	Gallus gallus domesticus	Domestic Chicken	H9	Positive	A	A/chicken/Bangladesh/32957/2017(H9N2)	Hatch Year	Healthy
Jahangirnagar University, Bangladesh	08/14/2011	Bangladesh	Dhaka	Katabon, Dhaka	Coturnix sp.	Quail	H9	Positive	A	A/environment/Bangladesh/12093/2011(H9N2)	Undetermined	Healthy
Jahangirnagar University-CEIRS2	01/23/2018	Bangladesh	Dhaka	Changkharpul, Dhaka	Coturnix sp.	Quail	H9	Positive	A	A/quail/Bangladesh/34615/2018(H9N2)	Hatch Year	Healthy
Jahangirnagar University, Bangladesh	06/28/2011	Bangladesh	Dhaka	Savar Bazar, Savar	Gallus gallus domesticus	Domestic Chicken	H9	Positive	A	A/environment/Bangladesh/11173/2011(H9N2)	Undetermined	Healthy
Jahangirnagar University-CEIRS2	12/25/2017	Bangladesh	Dhaka	Dhaka	Anas platyrhynchos	mallard	H9	Positive	A	A/duck/Bangladesh/34286/2017(H9N2)	Hatch Year	Healthy
Jahangirnagar University-CEIRS2	08/19/2019	Bangladesh	Dhaka	Mohammadpur, Dhaka	Gallus gallus domesticus	Domestic Chicken	H9	Positive	A	A/chicken/Bangladesh/40619/2019(H9N2)	Hatch Year	Healthy
Jahangirnagar University, Bangladesh	12/27/2009	Bangladesh	Dhaka	Katabon, Dhaka	Coturnix sp.	Quail	H9	Positive	A	A/environment/Bangladesh/5144/2009(H9N2)	Hatch Year	Healthy
Jahangirnagar University-CEIRS2	02/04/2016	Bangladesh	Dhaka	Savar Bazar, Savar	Anas sp.	Duck	H9	Positive	A	A/chicken/Bangladesh/30030/2016(H9N2)	Hatch Year	Healthy
Jahangirnagar University-CEIRS2	04/26/2018	Bangladesh	Dhaka	Changkharpul, Dhaka	Coturnix sp.	Quail	H9	Positive	A	A/quail/Bangladesh/35241/2018(H9N2)	Hatch Year	Healthy
Jahangirnagar University-CEIRS2	06/05/2018	Bangladesh	Dhaka	Changkharpul, Dhaka	Coturnix sp.	Quail	H9	Positive	A	A/quail/Bangladesh/35454/2018(H9N2)	Hatch Year	Healthy
Jahangirnagar University, Bangladesh	06/28/2011	Bangladesh	Dhaka	Savar Bazar, Savar	Gallus gallus domesticus	Domestic Chicken	H9	Positive	A	A/chicken/Bangladesh/11154/2011(H9N2),A/chicken/Bangladesh/11153/2011(H9N2)	Undetermined	Healthy
Jahangirnagar University-CEIRS2	01/23/2018	Bangladesh	Dhaka	Kaptan Bazar, Dhaka	Gallus gallus domesticus	Domestic Chicken	H9	Positive	A	A/environment/Bangladesh/34574/2018(H9N2)	Hatch Year	Healthy
Jahangirnagar University, Bangladesh	01/07/2009	Bangladesh	Dhaka	Katabon, Dhaka	Coturnix sp.	Quail	H9	Positive	A	A/environment/Bangladesh/907/2009(H9N2)	Hatch Year	Healthy
Jahangirnagar University, Bangladesh	09/01/2010	Bangladesh	Dhaka	Savar Bazar, Savar	Gallus gallus domesticus	Domestic Chicken	H9	Positive	A	A/environment/Bangladesh/8465/2010(H9N2)	Undetermined	Healthy
Jahangirnagar University-CEIRS2	11/25/2016	Bangladesh	Dhaka	Changkharpul, Dhaka	Coturnix sp.	Quail	H9	Positive	A	A/quail/Bangladesh/31244/2016(H9N2)	Hatch Year	Healthy
Jahangirnagar University-CEIRS2	09/27/2016	Bangladesh	Dhaka	Mohammadpur, Dhaka	Gallus gallus domesticus	Domestic Chicken	H9	Positive	A	A/chicken/Bangladesh/31066/2016(H9N2)	Hatch Year	Healthy
Jahangirnagar University-CEIRS2	10/24/2016	Bangladesh	Dhaka	Mohammadpur, Dhaka	Gallus gallus domesticus	Domestic Chicken	H9	Positive	A	A/chicken/Bangladesh/31066/2016(H9N2)	Hatch Year	Healthy
Jahangirnagar University, Bangladesh	02/19/2011	Bangladesh	Dhaka	Katabon, Dhaka	Coturnix sp.	Quail	H9	Positive	A	A/environment/Bangladesh/10313/2011(H9N2)	Undetermined	Healthy
Jahangirnagar University-CEIRS2	08/21/2016	Bangladesh	Dhaka	Changkharpul	Coturnix sp.	Quail	H9	Positive	A	A/chicken/Bangladesh/30861/2016(H9N2)	Hatch Year	Healthy
Jahangirnagar University-CEIRS2	12/18/2018	Bangladesh	Dhaka	Mohammadpur, Dhaka	Gallus gallus domesticus	Domestic Chicken	H9	Positive	A	A/chicken/Bangladesh/35970/2018(H9N2)	Hatch Year	Healthy
Jahangirnagar University-CEIRS2	11/28/2017	Bangladesh	Dhaka	Dhaka	Gallus gallus domesticus	Domestic Chicken	H9	Positive	A	A/chicken/Bangladesh/34004/2017(H9N2)	Hatch Year	Healthy
Jahangirnagar University-CEIRS2	06/05/2018	Bangladesh	Dhaka	Kaptan Bazar, Dhaka	Gallus gallus domesticus	Domestic Chicken	H9	Positive	A	A/chicken/Bangladesh/35410/2018(H9N2)	Hatch Year	Sick
Jahangirnagar University-CEIRS2	09/28/2017	Bangladesh	Dhaka	Dhaka	Gallus gallus domesticus	Domestic Chicken	H9	Positive	A	A/chicken/Bangladesh/33649/2017(H9N2)	Hatch Year	Healthy
Jahangirnagar University-CEIRS2	01/23/2018	Bangladesh	Dhaka	Kaptan Bazar, Dhaka	Gallus gallus domesticus	Domestic Chicken	H9	Positive	A	A/environment/Bangladesh/34578/2018(H9N2)	Hatch Year	Healthy
Jahangirnagar University-CEIRS2	06/05/2018	Bangladesh	Dhaka	Kaptan Bazar, Dhaka	Gallus gallus domesticus	Domestic Chicken	H9	Positive	A	A/chicken/Bangladesh/35407/2018(H9N2)	Hatch Year	Sick
Jahangirnagar University-CEIRS2	12/18/2018	Bangladesh	Dhaka	Changkharpul, Dhaka	Coturnix sp.	Quail	H9	Positive	A	A/quail/Bangladesh/35929/2018(H9N2)	Hatch Year	Healthy
Jahangirnagar University, Bangladesh	08/10/2009	Bangladesh	Dhaka	Karwan Bazaar, Dhaka	Columba livia	Rock Pigeon	H9	Positive	A	A/pigeon/Bangladesh/4303/2009(H9N2)	Undetermined	Healthy
Jahangirnagar University-CEIRS2	10/16/2019	Bangladesh	Dhaka	Kaptan Bazar, Dhaka	Gallus gallus domesticus	Domestic Chicken	H9	Positive	A	A/chicken/Bangladesh/40960/2019(H9N2)	Hatch Year	Healthy
Jahangirnagar University, Bangladesh	05/29/2011	Bangladesh	Dhaka	Karwan Bazaar, Dhaka	Gallus gallus domesticus	Domestic Chicken	H9	Positive	A	A/chicken/Bangladesh/10897/2011(H9N2)	Undetermined	Healthy
Jahangirnagar University-CEIRS2	08/27/2015	Bangladesh	Dhaka	Savar Bazar, Savar	Gallus gallus domesticus	Domestic Chicken	H9	Positive	A	A/chicken/Bangladesh/26115/2015(H9N2)	Undetermined	Healthy

Jahangirnagar University-CEIRS2	09/27/2016	Bangladesh	Dhaka	Changkharpul, Dhaka	Coturnix sp.	Quail	H9	Positive	A	A/quail/Bangladesh/31043/2016(H9N2)	Hatch Year	Healthy
Jahangirnagar University-CEIRS2	10/24/2016	Bangladesh	Dhaka	Changkharpul, Dhaka	Coturnix sp.	Quail	H9	Positive	A	A/quail/Bangladesh/31043/2016(H9N2)	Hatch Year	Healthy
Jahangirnagar University, Bangladesh	07/10/2010	Bangladesh	Dhaka	Savar Bazar, Savar	Gallus gallus domesticus	Domestic Chicken	H9	Positive	A	A/environment/Bangladesh/9457/2010(H9N2)	Undetermined	Healthy
Jahangirnagar University-CEIRS2	07/11/2019	Bangladesh	Dhaka	Changkharpul, Dhaka	Coturnix sp.	Quail	H9	Positive	A	A/quail/Bangladesh/40420/2019(H9N2)	Hatch Year	Healthy
Jahangirnagar University-CEIRS2	08/09/2018	Bangladesh	Dhaka	Mohammadpur, Dhaka	Gallus gallus domesticus	Domestic Chicken	H9	Positive	A	A/chicken/Bangladesh/35646/2018(H9N2)	Hatch Year	Healthy
Jahangirnagar University-CEIRS2	08/19/2019	Bangladesh	Dhaka	Mohammadpur, Dhaka	Gallus gallus domesticus	Domestic Chicken	H9	Positive	A	A/chicken/Bangladesh/40618/2019(H9N2)	Hatch Year	Healthy
Jahangirnagar University, Bangladesh	02/19/2011	Bangladesh	Dhaka	Katabon, Dhaka	Coturnix sp.	Quail	H9	Positive	A	A/environment/Bangladesh/10306/2011(H9N2)	Undetermined	Healthy
Jahangirnagar University-CEIRS2	11/28/2017	Bangladesh	Dhaka	Dhaka	Gallus gallus domesticus	Domestic Chicken	H9	Positive	A	A/environment/Bangladesh/34092/2017(H9N2)	Hatch Year	Healthy
Jahangirnagar University-CEIRS2	02/05/2016	Bangladesh	Dhaka	Farm, Narsingdi	Gallus gallus domesticus	Domestic Chicken	H9	Positive	A	A/quail/Bangladesh/29997/2016(H9N2)	Hatch Year	Healthy
Jahangirnagar University-CEIRS2	12/25/2017	Bangladesh	Dhaka	Dhaka	Gallus gallus domesticus	Domestic Chicken	H9	Positive	A	A/chicken/Bangladesh/34321/2017(H9N2)	Hatch Year	Healthy
Jahangirnagar University-CEIRS2	12/25/2017	Bangladesh	Dhaka	Dhaka	Gallus gallus domesticus	Domestic Chicken	H9	Positive	A	A/environment/Bangladesh/34343/2017(H9N2)	Hatch Year	Healthy
Jahangirnagar University-CEIRS2	04/28/2018	Bangladesh	Dhaka	Savar Bazar, Dhaka	Gallus gallus domesticus	Domestic Chicken	H9	Positive	A	A/chicken/Bangladesh/35368/2018(H9N2)	Hatch Year	Healthy
Jahangirnagar University-CEIRS2	11/28/2017	Bangladesh	Dhaka	Dhaka	Gallus gallus domesticus	Domestic Chicken	H9	Positive	A	A/chicken/Bangladesh/34075/2017(H9N2)	Hatch Year	Healthy
Jahangirnagar University-CEIRS2	02/15/2017	Bangladesh	Dhaka	Changkharpul, Dhaka	Coturnix sp.	Quail	H9	Positive	A	A/quail/Bangladesh/32020/2017(H9N2)	Hatch Year	Healthy
Jahangirnagar University-CEIRS2	01/23/2015	Bangladesh	Dhaka	Kaptan Bazar, Dhaka	Gallus gallus domesticus	Domestic Chicken	H9	Positive	A	A/environment/Bangladesh/24205/2015(H9N2)	Undetermined	Healthy
Jahangirnagar University-CEIRS2	10/30/2018	Bangladesh	Dhaka	Kaptan Bazar, Dhaka	Gallus gallus domesticus	Domestic Chicken	H9	Positive	A	A/chicken/Bangladesh/35732/2018(H9N2)	Hatch Year	Healthy
Jahangirnagar University-CEIRS2	08/21/2016	Bangladesh	Dhaka	Dhaka	Gallus gallus domesticus	Domestic Chicken	H9	Positive	A	A/quail/Bangladesh/30835/2016(H9N2)	Hatch Year	Healthy
Jahangirnagar University-CEIRS2	02/03/2016	Bangladesh	Dhaka	Kaptan Bazar, Dhaka	Anas sp.	Duck	H9	Positive	A	A/chicken/Bangladesh/28182/2016(H9N2)	Hatch Year	Healthy
Jahangirnagar University-CEIRS2	08/25/2015	Bangladesh	Dhaka	Changkharpul	Coturnix sp.	Quail	H9	Positive	A	A/quail/Bangladesh/25987/2015(H9N2)	Undetermined	Healthy
Jahangirnagar University-CEIRS2	06/05/2018	Bangladesh	Dhaka	Kaptan Bazar, Dhaka	Gallus gallus domesticus	Domestic Chicken	H9	Positive	A	A/chicken/Bangladesh/35417/2018(H9N2)	Hatch Year	Healthy
Jahangirnagar University-CEIRS2	09/28/2017	Bangladesh	Dhaka	Dhaka	Gallus gallus domesticus	Domestic Chicken	H9	Positive	A	A/chicken/Bangladesh/33648/2017(H9N2)	Hatch Year	Healthy
Jahangirnagar University, Bangladesh	07/20/2011	Bangladesh	Dhaka	Savar Bazar, Savar	Gallus gallus domesticus	Domestic Chicken	H9	Positive	A	A/environment/Bangladesh/11597/2011(H9N2)	Undetermined	Healthy
Jahangirnagar University, Bangladesh	02/19/2011	Bangladesh	Dhaka	Katabon, Dhaka	Coturnix sp.	Quail	H9	Positive	A	A/environment/Bangladesh/10307/2011(H9N2)	Undetermined	Healthy
Jahangirnagar University, Bangladesh	12/20/2013	Bangladesh	Dhaka	Farm, Narsingdi	Gallus gallus domesticus	Domestic Chicken	H9	Positive	A	A/chicken/Bangladesh/21554/2013(H9N2)	Undetermined	Healthy
Jahangirnagar University-CEIRS2	11/28/2017	Bangladesh	Dhaka	Dhaka	Gallus gallus domesticus	Domestic Chicken	H9	Positive	A	A/environment/Bangladesh/34095/2017(H9N2)	Hatch Year	Healthy
Jahangirnagar University-CEIRS2	08/25/2015	Bangladesh	Dhaka	Kaptan Bazar, Dhaka	Gallus gallus domesticus	Domestic Chicken	H9	Positive	A	A/chicken/Bangladesh/25946/2015(H9N2)	Undetermined	Healthy
Jahangirnagar University, Bangladesh	08/07/2010	Bangladesh	Barisal	Farm, Pandua	Gallus gallus domesticus	Domestic Chicken	H9	Positive	A	A/environment/Bangladesh/8202/2010(H9N2)	Undetermined	Healthy
Jahangirnagar University, Bangladesh	08/14/2011	Bangladesh	Dhaka	Katabon, Dhaka	Meleagris gallopavo	Wild Turkey	H9	Positive	A	A/environment/Bangladesh/12077/2011(H9N2)	Undetermined	Healthy
Jahangirnagar University, Bangladesh	05/25/2013	Bangladesh	Dhaka	Changkharpul	Coturnix sp.	Quail	H9	Positive	A	A/environment/Bangladesh/19842/2013(H9N2)	Undetermined	Healthy
Jahangirnagar University-CEIRS2	11/28/2017	Bangladesh	Dhaka	Dhaka	Coturnix sp.	Quail	H9	Positive	A	A/quail/Bangladesh/34042/2017(H9N2)	Hatch Year	Healthy
Jahangirnagar University-CEIRS2	12/29/2014	Bangladesh	Dhaka	Changkharpul	Coturnix sp.	Quail	H9	Positive	A	A/quail/Bangladesh/24008/2014(H9N2)	Undetermined	Healthy
Jahangirnagar University-CEIRS2	10/19/2014	Bangladesh	Dhaka	Savar Bazar, Savar	Gallus gallus domesticus	Domestic Chicken	H9	Positive	A	A/chicken/Bangladesh/23727/2014(H9N2)	Undetermined	Healthy
Jahangirnagar University-CEIRS2	03/28/2017	Bangladesh	Dhaka	Savar Bazar, Savar	Gallus gallus domesticus	Domestic Chicken	H9	Positive	A	A/chicken/Bangladesh/32390/2017(H9N2)	Hatch Year	Healthy
Jahangirnagar University, Bangladesh	09/01/2010	Bangladesh	Dhaka	Savar Bazar, Savar	Gallus gallus domesticus	Domestic Chicken	H9	Positive	A	A/environment/Bangladesh/8463/2010(H9N2)	Undetermined	Healthy
Jahangirnagar University, Bangladesh	11/24/2013	Bangladesh	Dhaka	Kaptan Bazar, Dhaka	Coturnix sp.	Quail	H9	Positive	A	A/quail/Bangladesh/21247/2013(H9N2)	Undetermined	Healthy
Jahangirnagar University-CEIRS2	12/25/2017	Bangladesh	Dhaka	Dhaka	Coturnix sp.	Quail	H9	Positive	A	A/quail/Bangladesh/34294/2017(H9N2)	Hatch Year	Healthy
Jahangirnagar University-CEIRS2	12/15/2018	Bangladesh	Dhaka	Savar Bazar, Dhaka	Gallus gallus domesticus	Domestic Chicken	H9	Positive	A	A/chicken/Bangladesh/36009/2018(H9N2)	Hatch Year	Healthy
Jahangirnagar University-CEIRS2	05/20/2017	Bangladesh	Dhaka	Mohammadpur, Dhaka	Gallus gallus domesticus	Domestic Chicken	H9	Positive	A	A/chicken/Bangladesh/32754/2017(H9N2)	Hatch Year	Healthy
Jahangirnagar University-CEIRS2	12/29/2014	Bangladesh	Dhaka	Changkharpul	Coturnix sp.	Quail	H9	Positive	A	A/quail/Bangladesh/24007/2014(H9N2)	Undetermined	Healthy
Jahangirnagar University, Bangladesh	08/14/2011	Bangladesh	Dhaka	Katabon, Dhaka	Coturnix sp.	Quail	H9	Positive	A	A/environment/Bangladesh/12103/2011(H9N2)	Undetermined	Healthy
Jahangirnagar University-CEIRS2	02/19/2019	Bangladesh	Dhaka	Kaptan Bazar, Dhaka	Gallus gallus domesticus	Domestic Chicken	H9	Positive	A	A/chicken/Bangladesh/38150/2019(H9N2)	Undetermined	Healthy
Jahangirnagar University-CEIRS2	08/25/2015	Bangladesh	Dhaka	Changkharpul	Coturnix sp.	Quail	H9	Positive	A	A/quail/Bangladesh/25997/2015(H9N2)	Undetermined	Healthy
Jahangirnagar University-CEIRS2	06/11/2017	Bangladesh	Dhaka	Mohammadpur, Dhaka	Gallus gallus domesticus	Domestic Chicken	H9	Positive	A	A/chicken/Bangladesh/32958/2017(H9N2)	Hatch Year	Healthy
Jahangirnagar University-CEIRS2	08/25/2015	Bangladesh	Dhaka	Changkharpul	Coturnix sp.	Quail	H9	Positive	A	A/quail/Bangladesh/25992/2015(H9N2)	Undetermined	Healthy
Jahangirnagar University, Bangladesh	10/15/2010	Bangladesh	Dhaka	Karwan Bazaar, Dhaka	Gallus gallus domesticus	Domestic Chicken	H9	Positive	A	A/chicken/Bangladesh/8996/2010(H9N2),A/chicken/Bangladesh/8995/2010(H9N2)	Undetermined	Healthy
Jahangirnagar University-CEIRS2	03/29/2017	Bangladesh	Dhaka	Mohammadpur, Dhaka	Gallus gallus domesticus	Domestic Chicken	H9	Positive	A	A/chicken/Bangladesh/32287/2017(H9N2)	Hatch Year	Healthy
Jahangirnagar University-CEIRS2	01/23/2018	Bangladesh	Dhaka	Kaptan Bazar, Dhaka	Gallus gallus domesticus	Domestic Chicken	H9	Positive	A	A/environment/Bangladesh/34573/2018(H9N2)	Hatch Year	Healthy
Jahangirnagar University-CEIRS2	12/18/2018	Bangladesh	Dhaka	Mohammadpur, Dhaka	Anas sp.	Duck	H9	Positive	A	A/duck/Bangladesh/35996/2018(H9N2)	Adult	Healthy
Jahangirnagar University, Bangladesh	05/25/2013	Bangladesh	Dhaka	Kaptan Bazar, Dhaka	Gallus gallus domesticus	Domestic Chicken	H9	Positive	A	A/chicken/Bangladesh/19870/2013(H9N2)	Undetermined	Healthy
Jahangirnagar University, Bangladesh	02/08/2011	Bangladesh	Dhaka	Savar Bazar, Savar	Gallus gallus domesticus	Domestic Chicken	H9	Positive	A	A/environment/Bangladesh/10234/2011(H9N2)	Undetermined	Healthy
Jahangirnagar University-CEIRS2	09/28/2017	Bangladesh	Dhaka	Dhaka	Gallus gallus domesticus	Domestic Chicken	H9	Positive	A	A/chicken/Bangladesh/33645/2017(H9N2)	Hatch Year	Healthy
Jahangirnagar University-CEIRS2	02/19/2019	Bangladesh	Dhaka	Mohammadpur, Dhaka	Gallus gallus domesticus	Domestic Chicken	H9	Positive	A	A/chicken/Bangladesh/38208/2019(H9N2)	Undetermined	Healthy
