# Supplementary material for: H9N2 avian influenza virus dispersal along Bangladeshi poultry trading networks
Source: Virus Evol. 2023 Feb 25;9(1):vead014. doi: 10.1093/ve/vead014 (PMC10032359; doi:10.1093/ve/vead014)
Supplement: vead014_Supp [file vead014_supp.zip › TableS4.pdf]

Isolate-ID	Country	Collection date	Originating Lab	Subtype	Submitting Lab	Authors
EPI_ISL_144241	Bangladesh	2009-Jan-07	NA	H9	Import from public-domain	Shanmuganatham,K.; Feeroz,M.; Jones-Engel,L.; Smith,G.J.D.; Fourment,M.; Walker,D.; McClenaghan,L.; Rabiul Alam,S.M.; Hasan,K.; Seiler,P.; Franks,J.; Danner,A.; Barman,S.; McKenzie,P.; Krauss,S.; Webby,R.J.; Webster,R.G.; Engel,L.J.; Alam,S.M.R.; Ferguson,A.
EPI_ISL_144228	Bangladesh	2009-Feb-03	NA	H9	Import from public-domain	Shanmuganatham,K.; Feeroz,M.; Jones-Engel,L.; Smith,G.J.D.; Fourment,M.; Walker,D.; McClenaghan,L.; Rabiul Alam,S.M.; Hasan,K.; Seiler,P.; Franks,J.; Danner,A.; Barman,S.; McKenzie,P.; Krauss,S.; Webby,R.J.; Webster,R.G.; Engel,L.J.; Alam,S.M.R.; Ferguson,A.
EPI_ISL_144265	Bangladesh	2009-Mar-22	NA	H9	Import from public-domain	Shanmuganatham,K.; Feeroz,M.; Jones-Engel,L.; Smith,G.J.D.; Fourment,M.; Walker,D.; McClenaghan,L.; Rabiul Alam,S.M.; Hasan,K.; Seiler,P.; Franks,J.; Danner,A.; Barman,S.; McKenzie,P.; Krauss,S.; Webby,R.J.; Webster,R.G.; Engel,L.J.; Alam,S.M.R.; Ferguson,A.
EPI_ISL_144268	Bangladesh	2009-Aug-10	NA	H9	Import from public-domain	Shanmuganatham,K.; Feeroz,M.; Jones-Engel,L.; Smith,G.J.D.; Fourment,M.; Walker,D.; McClenaghan,L.; Rabiul Alam,S.M.; Hasan,K.; Seiler,P.; Franks,J.; Danner,A.; Barman,S.; McKenzie,P.; Krauss,S.; Webby,R.J.; Webster,R.G.; Engel,L.J.; Alam,S.M.R.; Ferguson,A.
EPI_ISL_144269	Bangladesh	2009-Dec-27	NA	H9	Import from public-domain	Shanmuganatham,K.; Feeroz,M.; Jones-Engel,L.; Smith,G.J.D.; Fourment,M.; Walker,D.; McClenaghan,L.; Rabiul Alam,S.M.; Hasan,K.; Seiler,P.; Franks,J.; Danner,A.; Barman,S.; McKenzie,P.; Krauss,S.; Webby,R.J.; Webster,R.G.; Engel,L.J.; Alam,S.M.R.; Ferguson,A.
EPI_ISL_144270	Bangladesh	2009-Dec-27	NA	H9	Import from public-domain	Shanmuganatham,K.; Feeroz,M.; Jones-Engel,L.; Smith,G.J.D.; Fourment,M.; Walker,D.; McClenaghan,L.; Rabiul Alam,S.M.; Hasan,K.; Seiler,P.; Franks,J.; Danner,A.; Barman,S.; McKenzie,P.; Krauss,S.; Webby,R.J.; Webster,R.G.; Engel,L.J.; Alam,S.M.R.; Ferguson,A.
EPI_ISL_144239	Bangladesh	2010-Jan-20	NA	H9	Import from public-domain	Shanmuganatham,K.; Feeroz,M.; Jones-Engel,L.; Smith,G.J.D.; Fourment,M.; Walker,D.; McClenaghan,L.; Rabiul Alam,S.M.; Hasan,K.; Seiler,P.; Franks,J.; Danner,A.; Barman,S.; McKenzie,P.; Krauss,S.; Webby,R.J.; Webster,R.G.; Engel,L.J.; Alam,S.M.R.; Ferguson,A.
EPI_ISL_144278	Bangladesh	2010-Aug-06	NA	H9	Import from public-domain	Shanmuganatham,K.; Feeroz,M.; Jones-Engel,L.; Smith,G.J.D.; Fourment,M.; Walker,D.; McClenaghan,L.; Rabiul Alam,S.M.; Hasan,K.; Seiler,P.; Franks,J.; Danner,A.; Barman,S.; McKenzie,P.; Krauss,S.; Webby,R.J.; Webster,R.G.; Engel,L.J.; Alam,S.M.R.; Ferguson,A.
EPI_ISL_144276	Bangladesh	2010-Aug-06	NA	H9	Import from public-domain	Shanmuganatham,K.; Feeroz,M.; Jones-Engel,L.; Smith,G.J.D.; Fourment,M.; Walker,D.; McClenaghan,L.; Rabiul Alam,S.M.; Hasan,K.; Seiler,P.; Franks,J.; Danner,A.; Barman,S.; McKenzie,P.; Krauss,S.; Webby,R.J.; Webster,R.G.; Engel,L.J.; Alam,S.M.R.; Ferguson,A.
EPI_ISL_144234	Bangladesh	2010-Aug-06	NA	H9	Import from public-domain	Shanmuganatham,K.; Feeroz,M.; Jones-Engel,L.; Smith,G.J.D.; Fourment,M.; Walker,D.; McClenaghan,L.; Rabiul Alam,S.M.; Hasan,K.; Seiler,P.; Franks,J.; Danner,A.; Barman,S.; McKenzie,P.; Krauss,S.; Webby,R.J.; Webster,R.G.; Engel,L.J.; Alam,S.M.R.; Ferguson,A.
EPI_ISL_144273	Bangladesh	2010-Aug-07	NA	H9	Import from public-domain	Shanmuganatham,K.; Feeroz,M.; Jones-Engel,L.; Smith,G.J.D.; Fourment,M.; Walker,D.; McClenaghan,L.; Rabiul Alam,S.M.; Hasan,K.; Seiler,P.; Franks,J.; Danner,A.; Barman,S.; McKenzie,P.; Krauss,S.; Webby,R.J.; Webster,R.G.; Engel,L.J.; Alam,S.M.R.; Ferguson,A.
EPI_ISL_144274	Bangladesh	2010-Sep-01	NA	H9	Import from public-domain	Shanmuganatham,K.; Feeroz,M.; Jones-Engel,L.; Smith,G.J.D.; Fourment,M.; Walker,D.; McClenaghan,L.; Rabiul Alam,S.M.; Hasan,K.; Seiler,P.; Franks,J.; Danner,A.; Barman,S.; McKenzie,P.; Krauss,S.; Webby,R.J.; Webster,R.G.; Engel,L.J.; Alam,S.M.R.; Ferguson,A.
EPI_ISL_144263	Bangladesh	2010-Sep-01	NA	H9	Import from public-domain	Shanmuganatham,K.; Feeroz,M.; Jones-Engel,L.; Smith,G.J.D.; Fourment,M.; Walker,D.; McClenaghan,L.; Rabiul Alam,S.M.; Hasan,K.; Seiler,P.; Franks,J.; Danner,A.; Barman,S.; McKenzie,P.; Krauss,S.; Webby,R.J.; Webster,R.G.; Engel,L.J.; Alam,S.M.R.; Ferguson,A.
EPI_ISL_144272	Bangladesh	2010-Sep-01	NA	H9	Import from public-domain	Shanmuganatham,K.; Feeroz,M.; Jones-Engel,L.; Smith,G.J.D.; Fourment,M.; Walker,D.; McClenaghan,L.; Rabiul Alam,S.M.; Hasan,K.; Seiler,P.; Franks,J.; Danner,A.; Barman,S.; McKenzie,P.; Krauss,S.; Webby,R.J.; Webster,R.G.; Engel,L.J.; Alam,S.M.R.; Ferguson,A.
EPI_ISL_144229	Bangladesh	2010-Sep-01	NA	H9	Import from public-domain	Shanmuganatham,K.; Feeroz,M.; Jones-Engel,L.; Smith,G.J.D.; Fourment,M.; Walker,D.; McClenaghan,L.; Rabiul Alam,S.M.; Hasan,K.; Seiler,P.; Franks,J.; Danner,A.; Barman,S.; McKenzie,P.; Krauss,S.; Webby,R.J.; Webster,R.G.; Engel,L.J.; Alam,S.M.R.; Ferguson,A.
EPI_ISL_144266	Bangladesh	2010-Oct-15	NA	H9	Import from public-domain	Shanmuganatham,K.; Feeroz,M.; Jones-Engel,L.; Smith,G.J.D.; Fourment,M.; Walker,D.; McClenaghan,L.; Rabiul Alam,S.M.; Hasan,K.; Seiler,P.; Franks,J.; Danner,A.; Barman,S.; McKenzie,P.; Krauss,S.; Webby,R.J.; Webster,R.G.; Engel,L.J.; Alam,S.M.R.; Ferguson,A.
EPI_ISL_144261	Bangladesh	2010-Oct-15	NA	H9	Import from public-domain	Shanmuganatham,K.; Feeroz,M.; Jones-Engel,L.; Smith,G.J.D.; Fourment,M.; Walker,D.; McClenaghan,L.; Rabiul Alam,S.M.; Hasan,K.; Seiler,P.; Franks,J.; Danner,A.; Barman,S.; McKenzie,P.; Krauss,S.; Webby,R.J.; Webster,R.G.; Engel,L.J.; Alam,S.M.R.; Ferguson,A.
EPI_ISL_144279	Bangladesh	2010-Nov-15	NA	H9	Import from public-domain	Shanmuganatham,K.; Feeroz,M.; Jones-Engel,L.; Smith,G.J.D.; Fourment,M.; Walker,D.; McClenaghan,L.; Rabiul Alam,S.M.; Hasan,K.; Seiler,P.; Franks,J.; Danner,A.; Barman,S.; McKenzie,P.; Krauss,S.; Webby,R.J.; Webster,R.G.; Engel,L.J.; Alam,S.M.R.; Ferguson,A.
EPI_ISL_144237	Bangladesh	2010-Nov-15	NA	H9	Import from public-domain	Shanmuganatham,K.; Feeroz,M.; Jones-Engel,L.; Smith,G.J.D.; Fourment,M.; Walker,D.; McClenaghan,L.; Rabiul Alam,S.M.; Hasan,K.; Seiler,P.; Franks,J.; Danner,A.; Barman,S.; McKenzie,P.; Krauss,S.; Webby,R.J.; Webster,R.G.; Engel,L.J.; Alam,S.M.R.; Ferguson,A.
EPI_ISL_144236	Bangladesh	2010-Nov-15	NA	H9	Import from public-domain	Shanmuganatham,K.; Feeroz,M.; Jones-Engel,L.; Smith,G.J.D.; Fourment,M.; Walker,D.; McClenaghan,L.; Rabiul Alam,S.M.; Hasan,K.; Seiler,P.; Franks,J.; Danner,A.; Barman,S.; McKenzie,P.; Krauss,S.; Webby,R.J.; Webster,R.G.; Engel,L.J.; Alam,S.M.R.; Ferguson,A.
EPI_ISL_144259	Bangladesh	2010-Dec-09	NA	H9	Import from public-domain	Shanmuganatham,K.; Feeroz,M.; Jones-Engel,L.; Smith,G.J.D.; Fourment,M.; Walker,D.; McClenaghan,L.; Rabiul Alam,S.M.; Hasan,K.; Seiler,P.; Franks,J.; Danner,A.; Barman,S.; McKenzie,P.; Krauss,S.; Webby,R.J.; Webster,R.G.; Engel,L.J.; Alam,S.M.R.; Ferguson,A.
EPI_ISL_144281	Bangladesh	2011-Feb-08	NA	H9	Import from public-domain	Shanmuganatham,K.; Feeroz,M.; Jones-Engel,L.; Smith,G.J.D.; Fourment,M.; Walker,D.; McClenaghan,L.; Rabiul Alam,S.M.; Hasan,K.; Seiler,P.; Franks,J.; Danner,A.; Barman,S.; McKenzie,P.; Krauss,S.; Webby,R.J.; Webster,R.G.; Engel,L.J.; Alam,S.M.R.; Ferguson,A.

EPI_ISL_144285	Bangladesh	2011-Feb-19	NA	H9	Import from public-domain	Shanmuganatham,K.; Feeroz,M.; Jones-Engel,L.; Smith,G.J.D.; Fourment,M.; Walker,D.; McClenaghan,L.; Rabiul Alam,S.M.; Hasan,K.; Seiler,P.; Franks,J.; Danner,A.; Barman,S.; McKenzie,P.; Krauss,S.; Webby,R.J.; Webster,R.G.; Engel,L.J.; Alam,S.M.R.; Ferguson,A.
EPI_ISL_144282	Bangladesh	2011-Feb-19	NA	H9	Import from public-domain	Shanmuganatham,K.; Feeroz,M.; Jones-Engel,L.; Smith,G.J.D.; Fourment,M.; Walker,D.; McClenaghan,L.; Rabiul Alam,S.M.; Hasan,K.; Seiler,P.; Franks,J.; Danner,A.; Barman,S.; McKenzie,P.; Krauss,S.; Webby,R.J.; Webster,R.G.; Engel,L.J.; Alam,S.M.R.; Ferguson,A.
EPI_ISL_144267	Bangladesh	2011-Feb-19	NA	H9	Import from public-domain	Shanmuganatham,K.; Feeroz,M.; Jones-Engel,L.; Smith,G.J.D.; Fourment,M.; Walker,D.; McClenaghan,L.; Rabiul Alam,S.M.; Hasan,K.; Seiler,P.; Franks,J.; Danner,A.; Barman,S.; McKenzie,P.; Krauss,S.; Webby,R.J.; Webster,R.G.; Engel,L.J.; Alam,S.M.R.; Ferguson,A.
EPI_ISL_144260	Bangladesh	2011-Feb-19	NA	H9	Import from public-domain	Shanmuganatham,K.; Feeroz,M.; Jones-Engel,L.; Smith,G.J.D.; Fourment,M.; Walker,D.; McClenaghan,L.; Rabiul Alam,S.M.; Hasan,K.; Seiler,P.; Franks,J.; Danner,A.; Barman,S.; McKenzie,P.; Krauss,S.; Webby,R.J.; Webster,R.G.; Engel,L.J.; Alam,S.M.R.; Ferguson,A.
EPI_ISL_144255	Bangladesh	2011-Feb-19	NA	H9	Import from public-domain	Shanmuganatham,K.; Feeroz,M.; Jones-Engel,L.; Smith,G.J.D.; Fourment,M.; Walker,D.; McClenaghan,L.; Rabiul Alam,S.M.; Hasan,K.; Seiler,P.; Franks,J.; Danner,A.; Barman,S.; McKenzie,P.; Krauss,S.; Webby,R.J.; Webster,R.G.; Engel,L.J.; Alam,S.M.R.; Ferguson,A.
EPI_ISL_144235	Bangladesh	2011-Feb-19	NA	H9	Import from public-domain	Shanmuganatham,K.; Feeroz,M.; Jones-Engel,L.; Smith,G.J.D.; Fourment,M.; Walker,D.; McClenaghan,L.; Rabiul Alam,S.M.; Hasan,K.; Seiler,P.; Franks,J.; Danner,A.; Barman,S.; McKenzie,P.; Krauss,S.; Webby,R.J.; Webster,R.G.; Engel,L.J.; Alam,S.M.R.; Ferguson,A.
EPI_ISL_144271	Bangladesh	2011-May-29	NA	H9	Import from public-domain	Shanmuganatham,K.; Feeroz,M.; Jones-Engel,L.; Smith,G.J.D.; Fourment,M.; Walker,D.; McClenaghan,L.; Rabiul Alam,S.M.; Hasan,K.; Seiler,P.; Franks,J.; Danner,A.; Barman,S.; McKenzie,P.; Krauss,S.; Webby,R.J.; Webster,R.G.; Engel,L.J.; Alam,S.M.R.; Ferguson,A.
EPI_ISL_144283	Bangladesh	2011-Jun-28	NA	H9	Import from public-domain	Shanmuganatham,K.; Feeroz,M.; Jones-Engel,L.; Smith,G.J.D.; Fourment,M.; Walker,D.; McClenaghan,L.; Rabiul Alam,S.M.; Hasan,K.; Seiler,P.; Franks,J.; Danner,A.; Barman,S.; McKenzie,P.; Krauss,S.; Webby,R.J.; Webster,R.G.; Engel,L.J.; Alam,S.M.R.; Ferguson,A.
EPI_ISL_144242	Bangladesh	2011-Jun-28	NA	H9	Import from public-domain	Shanmuganatham,K.; Feeroz,M.; Jones-Engel,L.; Smith,G.J.D.; Fourment,M.; Walker,D.; McClenaghan,L.; Rabiul Alam,S.M.; Hasan,K.; Seiler,P.; Franks,J.; Danner,A.; Barman,S.; McKenzie,P.; Krauss,S.; Webby,R.J.; Webster,R.G.; Engel,L.J.; Alam,S.M.R.; Ferguson,A.
EPI_ISL_144284	Bangladesh	2011-Jun-30	NA	H9	Import from public-domain	Shanmuganatham,K.; Feeroz,M.; Jones-Engel,L.; Smith,G.J.D.; Fourment,M.; Walker,D.; McClenaghan,L.; Rabiul Alam,S.M.; Hasan,K.; Seiler,P.; Franks,J.; Danner,A.; Barman,S.; McKenzie,P.; Krauss,S.; Webby,R.J.; Webster,R.G.; Engel,L.J.; Alam,S.M.R.; Ferguson,A.
EPI_ISL_144238	Bangladesh	2011-Jun-30	NA	H9	Import from public-domain	Shanmuganatham,K.; Feeroz,M.; Jones-Engel,L.; Smith,G.J.D.; Fourment,M.; Walker,D.; McClenaghan,L.; Rabiul Alam,S.M.; Hasan,K.; Seiler,P.; Franks,J.; Danner,A.; Barman,S.; McKenzie,P.; Krauss,S.; Webby,R.J.; Webster,R.G.; Engel,L.J.; Alam,S.M.R.; Ferguson,A.
EPI_ISL_144280	Bangladesh	2011-Jul-20	NA	H9	Import from public-domain	Shanmuganatham,K.; Feeroz,M.; Jones-Engel,L.; Smith,G.J.D.; Fourment,M.; Walker,D.; McClenaghan,L.; Rabiul Alam,S.M.; Hasan,K.; Seiler,P.; Franks,J.; Danner,A.; Barman,S.; McKenzie,P.; Krauss,S.; Webby,R.J.; Webster,R.G.; Engel,L.J.; Alam,S.M.R.; Ferguson,A.
EPI_ISL_144264	Bangladesh	2011-Aug-14	NA	H9	Import from public-domain	Shanmuganatham,K.; Feeroz,M.; Jones-Engel,L.; Smith,G.J.D.; Fourment,M.; Walker,D.; McClenaghan,L.; Rabiul Alam,S.M.; Hasan,K.; Seiler,P.; Franks,J.; Danner,A.; Barman,S.; McKenzie,P.; Krauss,S.; Webby,R.J.; Webster,R.G.; Engel,L.J.; Alam,S.M.R.; Ferguson,A.
EPI_ISL_144262	Bangladesh	2011-Aug-14	NA	H9	Import from public-domain	Shanmuganatham,K.; Feeroz,M.; Jones-Engel,L.; Smith,G.J.D.; Fourment,M.; Walker,D.; McClenaghan,L.; Rabiul Alam,S.M.; Hasan,K.; Seiler,P.; Franks,J.; Danner,A.; Barman,S.; McKenzie,P.; Krauss,S.; Webby,R.J.; Webster,R.G.; Engel,L.J.; Alam,S.M.R.; Ferguson,A.
EPI_ISL_144258	Bangladesh	2011-Aug-14	NA	H9	Import from public-domain	Shanmuganatham,K.; Feeroz,M.; Jones-Engel,L.; Smith,G.J.D.; Fourment,M.; Walker,D.; McClenaghan,L.; Rabiul Alam,S.M.; Hasan,K.; Seiler,P.; Franks,J.; Danner,A.; Barman,S.; McKenzie,P.; Krauss,S.; Webby,R.J.; Webster,R.G.; Engel,L.J.; Alam,S.M.R.; Ferguson,A.
EPI_ISL_144240	Bangladesh	2011-Aug-14	NA	H9	Import from public-domain	Shanmuganatham,K.; Feeroz,M.; Jones-Engel,L.; Smith,G.J.D.; Fourment,M.; Walker,D.; McClenaghan,L.; Rabiul Alam,S.M.; Hasan,K.; Seiler,P.; Franks,J.; Danner,A.; Barman,S.; McKenzie,P.; Krauss,S.; Webby,R.J.; Webster,R.G.; Engel,L.J.; Alam,S.M.R.; Ferguson,A.
EPI_ISL_144233	Bangladesh	2011-Aug-14	NA	H9	Import from public-domain	Shanmuganatham,K.; Feeroz,M.; Jones-Engel,L.; Smith,G.J.D.; Fourment,M.; Walker,D.; McClenaghan,L.; Rabiul Alam,S.M.; Hasan,K.; Seiler,P.; Franks,J.; Danner,A.; Barman,S.; McKenzie,P.; Krauss,S.; Webby,R.J.; Webster,R.G.; Engel,L.J.; Alam,S.M.R.; Ferguson,A.
EPI_ISL_144231	Bangladesh	2011-Aug-14	NA	H9	Import from public-domain	Shanmuganatham,K.; Feeroz,M.; Jones-Engel,L.; Smith,G.J.D.; Fourment,M.; Walker,D.; McClenaghan,L.; Rabiul Alam,S.M.; Hasan,K.; Seiler,P.; Franks,J.; Danner,A.; Barman,S.; McKenzie,P.; Krauss,S.; Webby,R.J.; Webster,R.G.; Engel,L.J.; Alam,S.M.R.; Ferguson,A.
EPI_ISL_144275	Bangladesh	2011-Nov-24	NA	H9	Import from public-domain	Shanmuganatham,K.; Feeroz,M.; Jones-Engel,L.; Smith,G.J.D.; Fourment,M.; Walker,D.; McClenaghan,L.; Rabiul Alam,S.M.; Hasan,K.; Seiler,P.; Franks,J.; Danner,A.; Barman,S.; McKenzie,P.; Krauss,S.; Webby,R.J.; Webster,R.G.; Engel,L.J.; Alam,S.M.R.; Ferguson,A.
EPI_ISL_144230	Bangladesh	2011-Nov-24	NA	H9	./	Shanmuganatham,K.; Feeroz,M.; Jones-Engel,L.; Smith,G.J.D.; Fourment,M.; Walker,D.; McClenaghan,L.; Rabiul Alam,S.M.; Hasan,K.; Seiler,P.; Franks,J.; Danner,A.; Barman,S.; McKenzie,P.; Krauss,S.; Webby,R.J.; Webster,R.G.; Engel,L.J.; Alam,S.M.R.; Ferguson,A.
EPI_ISL_161232	Bangladesh	2012-Mar-22	NA	H9	Import from public-domain	Shanmuganatham,K.; Feeroz,M.; Jones Engel,L.; Walker,D.; McClenaghan,L.; Alam,R.S.; Hasan,K.; McKenzie,P.; Webby,R.J.; Webster,R.G.
EPI_ISL_161203	Bangladesh	2012-Oct-21	NA	H9	Import from public-domain	Shanmuganatham,K.; Feeroz,M.; Jones Engel,L.; Walker,D.; McClenaghan,L.; Alam,R.S.; Hasan,K.; McKenzie,P.; Webby,R.J.; Webster,R.G.
EPI_ISL_161233	Bangladesh	2012-Oct-21	NA	H9	Import from public-domain	Shanmuganatham,K.; Feeroz,M.; Jones Engel,L.; Walker,D.; McClenaghan,L.; Alam,R.S.; Hasan,K.; McKenzie,P.; Webby,R.J.; Webster,R.G.
EPI_ISL_161227	Bangladesh	2012-Oct-21	NA	H9	Import from public-domain	Shanmuganatham,K.; Feeroz,M.; Jones Engel,L.; Walker,D.; McClenaghan,L.; Alam,R.S.; Hasan,K.; McKenzie,P.; Webby,R.J.; Webster,R.G.

EPI_ISL_161201	Bangladesh	2012-Oct-21	NA	H9	Import from public-domain	Shanmuganatham,K.; Feeroz,M.; Jones Engel,L.; Walker,D.; McClenaghan,L.; Alam,R.S.; Hasan,K.; McKenzie,P.; Webby,R.J.; Webster,R.G.
EPI_ISL_161236	Bangladesh	2012-Oct-21	NA	H9	Import from public-domain	Shanmuganatham,K.; Feeroz,M.; Jones Engel,L.; Walker,D.; McClenaghan,L.; Alam,R.S.; Hasan,K.; McKenzie,P.; Webby,R.J.; Webster,R.G.
EPI_ISL_161235	Bangladesh	2012-Oct-21	NA	H9	Import from public-domain	Shanmuganatham,K.; Feeroz,M.; Jones Engel,L.; Walker,D.; McClenaghan,L.; Alam,R.S.; Hasan,K.; McKenzie,P.; Webby,R.J.; Webster,R.G.
EPI_ISL_161224	Bangladesh	2012-Oct-21	NA	H9	Import from public-domain	Shanmuganatham,K.; Feeroz,M.; Jones Engel,L.; Walker,D.; McClenaghan,L.; Alam,R.S.; Hasan,K.; McKenzie,P.; Webby,R.J.; Webster,R.G.
EPI_ISL_161216	Bangladesh	2012-Oct-21	NA	H9	Import from public-domain	Shanmuganatham,K.; Feeroz,M.; Jones Engel,L.; Walker,D.; McClenaghan,L.; Alam,R.S.; Hasan,K.; McKenzie,P.; Webby,R.J.; Webster,R.G.
EPI_ISL_161231	Bangladesh	2012-Nov-15	NA	H9	Import from public-domain	Shanmuganatham,K.; Feeroz,M.; Jones Engel,L.; Walker,D.; McClenaghan,L.; Alam,R.S.; Hasan,K.; McKenzie,P.; Webby,R.J.; Webster,R.G.
EPI_ISL_161221	Bangladesh	2012-Nov-15	NA	H9	Import from public-domain	Shanmuganatham,K.; Feeroz,M.; Jones Engel,L.; Walker,D.; McClenaghan,L.; Alam,R.S.; Hasan,K.; McKenzie,P.; Webby,R.J.; Webster,R.G.
EPI_ISL_161199	Bangladesh	2012-Nov-20	NA	H9	Import from public-domain	Shanmuganatham,K.; Feeroz,M.; Jones Engel,L.; Walker,D.; McClenaghan,L.; Alam,R.S.; Hasan,K.; McKenzie,P.; Webby,R.J.; Webster,R.G.
EPI_ISL_161228	Bangladesh	2012-Nov-25	NA	H9	Import from public-domain	Shanmuganatham,K.; Feeroz,M.; Jones Engel,L.; Walker,D.; McClenaghan,L.; Alam,R.S.; Hasan,K.; McKenzie,P.; Webby,R.J.; Webster,R.G.
EPI_ISL_161202	Bangladesh	2012-Nov-25	NA	H9	Import from public-domain	Shanmuganatham,K.; Feeroz,M.; Jones Engel,L.; Walker,D.; McClenaghan,L.; Alam,R.S.; Hasan,K.; McKenzie,P.; Webby,R.J.; Webster,R.G.
EPI_ISL_161229	Bangladesh	2012-Nov-25	NA	H9	Import from public-domain	Shanmuganatham,K.; Feeroz,M.; Jones Engel,L.; Walker,D.; McClenaghan,L.; Alam,R.S.; Hasan,K.; McKenzie,P.; Webby,R.J.; Webster,R.G.
EPI_ISL_161237	Bangladesh	2013-Jan-28	NA	H9	Import from public-domain	Shanmuganatham,K.; Feeroz,M.; Jones Engel,L.; Walker,D.; McClenaghan,L.; Alam,R.S.; Hasan,K.; McKenzie,P.; Webby,R.J.; Webster,R.G.
EPI_ISL_161234	Bangladesh	2013-Jan-28	NA	H9	Import from public-domain	Shanmuganatham,K.; Feeroz,M.; Jones Engel,L.; Walker,D.; McClenaghan,L.; Alam,R.S.; Hasan,K.; McKenzie,P.; Webby,R.J.; Webster,R.G.
EPI_ISL_161200	Bangladesh	2013-Jan-28	NA	H9	Import from public-domain	Shanmuganatham,K.; Feeroz,M.; Jones Engel,L.; Walker,D.; McClenaghan,L.; Alam,R.S.; Hasan,K.; McKenzie,P.; Webby,R.J.; Webster,R.G.
EPI_ISL_161230	Bangladesh	2013-Jan-28	NA	H9	Import from public-domain	Shanmuganatham,K.; Feeroz,M.; Jones Engel,L.; Walker,D.; McClenaghan,L.; Alam,R.S.; Hasan,K.; McKenzie,P.; Webby,R.J.; Webster,R.G.
EPI_ISL_161226	Bangladesh	2013-Feb-14	NA	H9	Import from public-domain	Shanmuganatham,K.; Feeroz,M.; Jones Engel,L.; Walker,D.; McClenaghan,L.; Alam,R.S.; Hasan,K.; McKenzie,P.; Webby,R.J.; Webster,R.G.
EPI_ISL_161198	Bangladesh	2013-Mar-30	NA	H9	Import from public-domain	Shanmuganatham,K.; Feeroz,M.; Jones Engel,L.; Walker,D.; McClenaghan,L.; Alam,R.S.; Hasan,K.; McKenzie,P.; Webby,R.J.; Webster,R.G.
EPI_ISL_161220	Bangladesh	2013-Mar-30	NA	H9	Import from public-domain	Shanmuganatham,K.; Feeroz,M.; Jones Engel,L.; Walker,D.; McClenaghan,L.; Alam,R.S.; Hasan,K.; McKenzie,P.; Webby,R.J.; Webster,R.G.
EPI_ISL_161219	Bangladesh	2013-Apr-29	NA	H9	Import from public-domain	Shanmuganatham,K.; Feeroz,M.; Jones Engel,L.; Walker,D.; McClenaghan,L.; Alam,R.S.; Hasan,K.; McKenzie,P.; Webby,R.J.; Webster,R.G.
EPI_ISL_161223	Bangladesh	2013-Apr-29	NA	H9	Import from public-domain	Shanmuganatham,K.; Feeroz,M.; Jones Engel,L.; Walker,D.; McClenaghan,L.; Alam,R.S.; Hasan,K.; McKenzie,P.; Webby,R.J.; Webster,R.G.
EPI_ISL_161222	Bangladesh	2013-Apr-29	NA	H9	Import from public-domain	Shanmuganatham,K.; Feeroz,M.; Jones Engel,L.; Walker,D.; McClenaghan,L.; Alam,R.S.; Hasan,K.; McKenzie,P.; Webby,R.J.; Webster,R.G.
EPI_ISL_161217	Bangladesh	2013-Apr-29	NA	H9	Import from public-domain	Shanmuganatham,K.; Feeroz,M.; Jones Engel,L.; Walker,D.; McClenaghan,L.; Alam,R.S.; Hasan,K.; McKenzie,P.; Webby,R.J.; Webster,R.G.
EPI_ISL_161225	Bangladesh	2013-May-25	NA	H9	Import from public-domain	Shanmuganatham,K.; Feeroz,M.; Jones Engel,L.; Walker,D.; McClenaghan,L.; Alam,R.S.; Hasan,K.; McKenzie,P.; Webby,R.J.; Webster,R.G.
EPI_ISL_161197	Bangladesh	2013-May-25	NA	H9	Import from public-domain	Shanmuganatham,K.; Feeroz,M.; Jones Engel,L.; Walker,D.; McClenaghan,L.; Alam,R.S.; Hasan,K.; McKenzie,P.; Webby,R.J.; Webster,R.G.
EPI_ISL_161218	Bangladesh	2013-Jul-08	NA	H9	Import from public-domain	Shanmuganatham,K.; Feeroz,M.; Jones Engel,L.; Walker,D.; McClenaghan,L.; Alam,R.S.; Hasan,K.; McKenzie,P.; Webby,R.J.; Webster,R.G.
EPI_ISL_161238	Bangladesh	2013-Jul-08	NA	H9	Import from public-domain	Shanmuganatham,K.; Feeroz,M.; Jones Engel,L.; Walker,D.; McClenaghan,L.; Alam,R.S.; Hasan,K.; McKenzie,P.; Webby,R.J.; Webster,R.G.
EPI_ISL_161685	Bangladesh	2013-Jul-08	NA	H9	Import from public-domain	Shanmuganatham,K.; Feeroz,M.; Jones Engel,L.; Walker,D.; McClenaghan,L.; Alam,R.S.; Hasan,K.; McKenzie,P.; Webby,R.J.; Webster,R.G.
EPI_ISL_161688	Bangladesh	2013-Oct-25	NA	H9	Import from public-domain	Shanmuganatham,K.; Feeroz,M.; Jones Engel,L.; Walker,D.; McClenaghan,L.; Alam,R.S.; Hasan,K.; McKenzie,P.; Webby,R.J.; Webster,R.G.
EPI_ISL_161686	Bangladesh	2013-Nov-24	NA	H9	Import from public-domain	Shanmuganatham,K.; Feeroz,M.; Jones Engel,L.; Walker,D.; McClenaghan,L.; Alam,R.S.; Hasan,K.; McKenzie,P.; Webby,R.J.; Webster,R.G.
EPI_ISL_161684	Bangladesh	2013-Dec-20	NA	H9	Import from public-domain	Shanmuganatham,K.; Feeroz,M.; Jones Engel,L.; Walker,D.; McClenaghan,L.; Alam,R.S.; Hasan,K.; McKenzie,P.; Webby,R.J.; Webster,R.G.

EPI_ISL_161687	Bangladesh	2013-Dec-27	NA	H9	Import from public-domain	Shanmuganatham,K.; Feeroz,M.; Jones Engel,L.; Walker,D.; McClenaghan,L.; Alam,R.S.; Hasan,K.; McKenzie,P.; Webby,R.J.; Webster,R.G.
EPI_ISL_256790	Bangladesh	2014-Oct-18	NA	H9	Import from public-domain	Shanmuganatham,K.; Barman,S.; Marinova-Petkova,A.; Hasan,M.K.; Akhtar,S.; Turner,J.C.; Franks,J.; Walker,D.; Seiler,P.; Friedman,K.; Jones-Engel,L.; McKenzie,P.; Krauss,S.; Webby,R.J.; Feeroz,M.M.; Webster,R.G.
EPI_ISL_256787	Bangladesh	2014-Oct-19	NA	H9	Import from public-domain	Shanmuganatham,K.; Barman,S.; Marinova-Petkova,A.; Hasan,M.K.; Akhtar,S.; Turner,J.C.; Franks,J.; Walker,D.; Seiler,P.; Friedman,K.; Jones-Engel,L.; McKenzie,P.; Krauss,S.; Webby,R.J.; Feeroz,M.M.; Webster,R.G.
EPI_ISL_256786	Bangladesh	2014-Oct-19	NA	H9	Import from public-domain	Shanmuganatham,K.; Barman,S.; Marinova-Petkova,A.; Hasan,M.K.; Akhtar,S.; Turner,J.C.; Franks,J.; Walker,D.; Seiler,P.; Friedman,K.; Jones-Engel,L.; McKenzie,P.; Krauss,S.; Webby,R.J.; Feeroz,M.M.; Webster,R.G.
EPI_ISL_256791	Bangladesh	2014-Dec-29	NA	H9	Import from public-domain	Shanmuganatham,K.; Barman,S.; Marinova-Petkova,A.; Hasan,M.K.; Akhtar,S.; Turner,J.C.; Franks,J.; Walker,D.; Seiler,P.; Friedman,K.; Jones-Engel,L.; McKenzie,P.; Krauss,S.; Webby,R.J.; Feeroz,M.M.; Webster,R.G.
EPI_ISL_256789	Bangladesh	2014-Dec-29	NA	H9	Import from public-domain	Shanmuganatham,K.; Barman,S.; Marinova-Petkova,A.; Hasan,M.K.; Akhtar,S.; Turner,J.C.; Franks,J.; Walker,D.; Seiler,P.; Friedman,K.; Jones-Engel,L.; McKenzie,P.; Krauss,S.; Webby,R.J.; Feeroz,M.M.; Webster,R.G.
EPI_ISL_257103	Bangladesh	2015-Jan-23	NA	H9	Import from public-domain	Shanmuganatham,K.; Barman,S.; Marinova-Petkova,A.; Hasan,M.K.; Akhtar,S.; Turner,J.C.; Franks,J.; Walker,D.; Seiler,P.; Friedman,K.; Jones-Engel,L.; McKenzie,P.; Krauss,S.; Webby,R.J.; Feeroz,M.M.; Webster,R.G.
EPI_ISL_257094	Bangladesh	2015-Jan-23	NA	H9	Import from public-domain	Shanmuganatham,K.; Barman,S.; Marinova-Petkova,A.; Hasan,M.K.; Akhtar,S.; Turner,J.C.; Franks,J.; Walker,D.; Seiler,P.; Friedman,K.; Jones-Engel,L.; McKenzie,P.; Krauss,S.; Webby,R.J.; Feeroz,M.M.; Webster,R.G.
EPI_ISL_257091	Bangladesh	2015-Jan-23	NA	H9	Import from public-domain	Shanmuganatham,K.; Barman,S.; Marinova-Petkova,A.; Hasan,M.K.; Akhtar,S.; Turner,J.C.; Franks,J.; Walker,D.; Seiler,P.; Friedman,K.; Jones-Engel,L.; McKenzie,P.; Krauss,S.; Webby,R.J.; Feeroz,M.M.; Webster,R.G.
EPI_ISL_257102	Bangladesh	2015-Mar-22	NA	H9	Import from public-domain	Shanmuganatham,K.; Barman,S.; Marinova-Petkova,A.; Hasan,M.K.; Akhtar,S.; Turner,J.C.; Franks,J.; Walker,D.; Seiler,P.; Friedman,K.; Jones-Engel,L.; McKenzie,P.; Krauss,S.; Webby,R.J.; Feeroz,M.M.; Webster,R.G.
EPI_ISL_257100	Bangladesh	2015-Mar-22	NA	H9	Import from public-domain	Shanmuganatham,K.; Barman,S.; Marinova-Petkova,A.; Hasan,M.K.; Akhtar,S.; Turner,J.C.; Franks,J.; Walker,D.; Seiler,P.; Friedman,K.; Jones-Engel,L.; McKenzie,P.; Krauss,S.; Webby,R.J.; Feeroz,M.M.; Webster,R.G.
EPI_ISL_257068	Bangladesh	2015-Mar-22	NA	H9	Import from public-domain	Shanmuganatham,K.; Barman,S.; Marinova-Petkova,A.; Hasan,M.K.; Akhtar,S.; Turner,J.C.; Franks,J.; Walker,D.; Seiler,P.; Friedman,K.; Jones-Engel,L.; McKenzie,P.; Krauss,S.; Webby,R.J.; Feeroz,M.M.; Webster,R.G.
EPI_ISL_257106	Bangladesh	2015-Aug-25	NA	H9	Import from public-domain	Barman,S.; Marinova-Petkova,A.; Hasan,M.K.; Akhtar,S.; Turner,J.C.; Franks,J.; Walker,D.; Seiler,P.; Friedman,K.; Kercher,L.; Kayali,G.; Jones-Engel,L.; McKenzie,P.; Krauss,S.; Webby,R.J.; Feeroz,M.M.; Webster,R.G.
EPI_ISL_257101	Bangladesh	2015-Aug-25	NA	H9	Import from public-domain	Barman,S.; Marinova-Petkova,A.; Hasan,M.K.; Akhtar,S.; Turner,J.C.; Franks,J.; Walker,D.; Seiler,P.; Friedman,K.; Kercher,L.; Kayali,G.; Jones-Engel,L.; McKenzie,P.; Krauss,S.; Webby,R.J.; Feeroz,M.M.; Webster,R.G.
EPI_ISL_257083	Bangladesh	2015-Aug-25	NA	H9	Import from public-domain	Barman,S.; Marinova-Petkova,A.; Hasan,M.K.; Akhtar,S.; Turner,J.C.; Franks,J.; Walker,D.; Seiler,P.; Friedman,K.; Kercher,L.; Kayali,G.; Jones-Engel,L.; McKenzie,P.; Krauss,S.; Webby,R.J.; Feeroz,M.M.; Webster,R.G.
EPI_ISL_257107	Bangladesh	2015-Aug-25	NA	H9	Import from public-domain	Barman,S.; Marinova-Petkova,A.; Hasan,M.K.; Akhtar,S.; Turner,J.C.; Franks,J.; Walker,D.; Seiler,P.; Friedman,K.; Kercher,L.; Kayali,G.; Jones-Engel,L.; McKenzie,P.; Krauss,S.; Webby,R.J.; Feeroz,M.M.; Webster,R.G.
EPI_ISL_257086	Bangladesh	2015-Aug-25	NA	H9	Import from public-domain	Barman,S.; Marinova-Petkova,A.; Hasan,M.K.; Akhtar,S.; Turner,J.C.; Franks,J.; Walker,D.; Seiler,P.; Friedman,K.; Kercher,L.; Kayali,G.; Jones-Engel,L.; McKenzie,P.; Krauss,S.; Webby,R.J.; Feeroz,M.M.; Webster,R.G.
EPI_ISL_257099	Bangladesh	2015-Aug-25	NA	H9	Import from public-domain	Barman,S.; Marinova-Petkova,A.; Hasan,M.K.; Akhtar,S.; Turner,J.C.; Franks,J.; Walker,D.; Seiler,P.; Friedman,K.; Kercher,L.; Kayali,G.; Jones-Engel,L.; McKenzie,P.; Krauss,S.; Webby,R.J.; Feeroz,M.M.; Webster,R.G.
EPI_ISL_257063	Bangladesh	2015-Aug-25	NA	H9	Import from public-domain	Barman,S.; Marinova-Petkova,A.; Hasan,M.K.; Akhtar,S.; Turner,J.C.; Franks,J.; Walker,D.; Seiler,P.; Friedman,K.; Kercher,L.; Kayali,G.; Jones-Engel,L.; McKenzie,P.; Krauss,S.; Webby,R.J.; Feeroz,M.M.; Webster,R.G.
EPI_ISL_257045	Bangladesh	2015-Aug-27	NA	H9	Import from public-domain	Barman,S.; Marinova-Petkova,A.; Hasan,M.K.; Akhtar,S.; Turner,J.C.; Franks,J.; Walker,D.; Seiler,P.; Friedman,K.; Kercher,L.; Kayali,G.; Jones-Engel,L.; McKenzie,P.; Krauss,S.; Webby,R.J.; Feeroz,M.M.; Webster,R.G.
EPI_ISL_257059	Bangladesh	2015-Aug-27	NA	H9	Import from public-domain	Barman,S.; Marinova-Petkova,A.; Hasan,M.K.; Akhtar,S.; Turner,J.C.; Franks,J.; Walker,D.; Seiler,P.; Friedman,K.; Kercher,L.; Kayali,G.; Jones-Engel,L.; McKenzie,P.; Krauss,S.; Webby,R.J.; Feeroz,M.M.; Webster,R.G.
EPI_ISL_257051	Bangladesh	2015-Aug-27	NA	H9	Import from public-domain	Barman,S.; Marinova-Petkova,A.; Hasan,M.K.; Akhtar,S.; Turner,J.C.; Franks,J.; Walker,D.; Seiler,P.; Friedman,K.; Kercher,L.; Kayali,G.; Jones-Engel,L.; McKenzie,P.; Krauss,S.; Webby,R.J.; Feeroz,M.M.; Webster,R.G.
EPI_ISL_257081	Bangladesh	2015-Sep-12	NA	H9	Import from public-domain	Barman,S.; Marinova-Petkova,A.; Hasan,M.K.; Akhtar,S.; Turner,J.C.; Franks,J.; Walker,D.; Seiler,P.; Friedman,K.; Kercher,L.; Kayali,G.; Jones-Engel,L.; McKenzie,P.; Krauss,S.; Webby,R.J.; Feeroz,M.M.; Webster,R.G.

EPI_ISL_257044	Bangladesh	2015-Sep-12	NA	H9	Import from public-domain	Barman,S.; Marinova-Petkova,A.; Hasan,M.K.; Akhtar,S.; Turner,J.C.; Franks,J.; Walker,D.; Seiler,P.; Friedman,K.; Kercher,L.; Kayali,G.; Jones-Engel,L.; McKenzie,P.; Krauss,S.; Webby,R.J.; Feeroz,M.M.; Webster,R.G.
EPI_ISL_257085	Bangladesh	2015-Dec-23	NA	H9	Import from public-domain	Barman,S.; Marinova-Petkova,A.; Hasan,M.K.; Akhtar,S.; Turner,J.C.; Franks,J.; Walker,D.; Seiler,P.; Friedman,K.; Kercher,L.; Kayali,G.; Jones-Engel,L.; McKenzie,P.; Krauss,S.; Webby,R.J.; Feeroz,M.M.; Webster,R.G.
EPI_ISL_257061	Bangladesh	2015-Dec-23	NA	H9	Import from public-domain	Barman,S.; Marinova-Petkova,A.; Hasan,M.K.; Akhtar,S.; Turner,J.C.; Franks,J.; Walker,D.; Seiler,P.; Friedman,K.; Kercher,L.; Kayali,G.; Jones-Engel,L.; McKenzie,P.; Krauss,S.; Webby,R.J.; Feeroz,M.M.; Webster,R.G.
EPI_ISL_257084	Bangladesh	2016-Feb-03	NA	H9	Import from public-domain	Barman,S.; Marinova-Petkova,A.; Hasan,M.K.; Akhtar,S.; Turner,J.C.; Franks,J.; Walker,D.; Seiler,P.; Friedman,K.; Kercher,L.; Kayali,G.; Jones-Engel,L.; McKenzie,P.; Krauss,S.; Webby,R.J.; Feeroz,M.M.; Webster,R.G.
EPI_ISL_257062	Bangladesh	2016-Feb-05	NA	H9	Import from public-domain	Barman,S.; Marinova-Petkova,A.; Hasan,M.K.; Akhtar,S.; Turner,J.C.; Franks,J.; Walker,D.; Seiler,P.; Friedman,K.; Kercher,L.; Kayali,G.; Jones-Engel,L.; McKenzie,P.; Krauss,S.; Webby,R.J.; Feeroz,M.M.; Webster,R.G.
EPI_ISL_284800	Bangladesh	2016-Mar-28	NA	H9	Import from public-domain	Barman,S.; Turner,J.C.; Hasan,M.K.; Akhtar,S.; Franks,J.; El-Shesheny,R.; Walker,D.; Seiler,P.; Friedman,K.; Kercher,L.; Kayali,G.; Jones-Engel,L.; McKenzie,P.; Krauss,S.; Webby,R.J.; Feeroz,M.M.; Webster,R.G.
EPI_ISL_284713	Bangladesh	2016-Mar-28	NA	H9	Import from public-domain	Barman,S.; Turner,J.C.; Hasan,M.K.; Akhtar,S.; Franks,J.; El-Shesheny,R.; Walker,D.; Seiler,P.; Friedman,K.; Kercher,L.; Kayali,G.; Jones-Engel,L.; McKenzie,P.; Krauss,S.; Webby,R.J.; Feeroz,M.M.; Webster,R.G.
EPI_ISL_284811	Bangladesh	2016-May-26	NA	H9	Import from public-domain	Barman,S.; Turner,J.C.; Hasan,M.K.; Akhtar,S.; Franks,J.; El-Shesheny,R.; Walker,D.; Seiler,P.; Friedman,K.; Kercher,L.; Kayali,G.; Jones-Engel,L.; McKenzie,P.; Krauss,S.; Webby,R.J.; Feeroz,M.M.; Webster,R.G.
EPI_ISL_284796	Bangladesh	2016-May-26	NA	H9	Import from public-domain	Barman,S.; Turner,J.C.; Hasan,M.K.; Akhtar,S.; Franks,J.; El-Shesheny,R.; Walker,D.; Seiler,P.; Friedman,K.; Kercher,L.; Kayali,G.; Jones-Engel,L.; McKenzie,P.; Krauss,S.; Webby,R.J.; Feeroz,M.M.; Webster,R.G.
EPI_ISL_284805	Bangladesh	2016-Jul-22	NA	H9	Import from public-domain	Barman,S.; Turner,J.C.; Hasan,M.K.; Akhtar,S.; Franks,J.; El-Shesheny,R.; Walker,D.; Seiler,P.; Friedman,K.; Kercher,L.; Kayali,G.; Jones-Engel,L.; McKenzie,P.; Krauss,S.; Webby,R.J.; Feeroz,M.M.; Webster,R.G.
EPI_ISL_284711	Bangladesh	2016-Jul-22	NA	H9	Import from public-domain	Barman,S.; Turner,J.C.; Hasan,M.K.; Akhtar,S.; Franks,J.; El-Shesheny,R.; Walker,D.; Seiler,P.; Friedman,K.; Kercher,L.; Kayali,G.; Jones-Engel,L.; McKenzie,P.; Krauss,S.; Webby,R.J.; Feeroz,M.M.; Webster,R.G.
EPI_ISL_284798	Bangladesh	2016-Aug-21	NA	H9	Import from public-domain	Barman,S.; Turner,J.C.; Hasan,M.K.; Akhtar,S.; Franks,J.; El-Shesheny,R.; Walker,D.; Seiler,P.; Friedman,K.; Kercher,L.; Kayali,G.; Jones-Engel,L.; McKenzie,P.; Krauss,S.; Webby,R.J.; Feeroz,M.M.; Webster,R.G.
EPI_ISL_284810	Bangladesh	2016-Sep-27	NA	H9	Import from public-domain	Barman,S.; Turner,J.C.; Hasan,M.K.; Akhtar,S.; Franks,J.; El-Shesheny,R.; Walker,D.; Seiler,P.; Friedman,K.; Kercher,L.; Kayali,G.; Jones-Engel,L.; McKenzie,P.; Krauss,S.; Webby,R.J.; Feeroz,M.M.; Webster,R.G.
EPI_ISL_284772	Bangladesh	2016-Sep-27	NA	H9	Import from public-domain	Barman,S.; Turner,J.C.; Hasan,M.K.; Akhtar,S.; Franks,J.; El-Shesheny,R.; Walker,D.; Seiler,P.; Friedman,K.; Kercher,L.; Kayali,G.; Jones-Engel,L.; McKenzie,P.; Krauss,S.; Webby,R.J.; Feeroz,M.M.; Webster,R.G.
EPI_ISL_284809	Bangladesh	2016-Oct-24	NA	H9	Import from public-domain	Barman,S.; Turner,J.C.; Hasan,M.K.; Akhtar,S.; Franks,J.; El-Shesheny,R.; Walker,D.; Seiler,P.; Friedman,K.; Kercher,L.; Kayali,G.; Jones-Engel,L.; McKenzie,P.; Krauss,S.; Webby,R.J.; Feeroz,M.M.; Webster,R.G.
EPI_ISL_284791	Bangladesh	2016-Oct-24	NA	H9	Import from public-domain	Barman,S.; Turner,J.C.; Hasan,M.K.; Akhtar,S.; Franks,J.; El-Shesheny,R.; Walker,D.; Seiler,P.; Friedman,K.; Kercher,L.; Kayali,G.; Jones-Engel,L.; McKenzie,P.; Krauss,S.; Webby,R.J.; Feeroz,M.M.; Webster,R.G.
EPI_ISL_284797	Bangladesh	2016-Nov-25	NA	H9	Import from public-domain	Barman,S.; Turner,J.C.; Hasan,M.K.; Akhtar,S.; Franks,J.; El-Shesheny,R.; Walker,D.; Seiler,P.; Friedman,K.; Kercher,L.; Kayali,G.; Jones-Engel,L.; McKenzie,P.; Krauss,S.; Webby,R.J.; Feeroz,M.M.; Webster,R.G.
EPI_ISL_284715	Bangladesh	2016-Dec-28	NA	H9	Import from public-domain	Barman,S.; Turner,J.C.; Hasan,M.K.; Akhtar,S.; Franks,J.; El-Shesheny,R.; Walker,D.; Seiler,P.; Friedman,K.; Kercher,L.; Kayali,G.; Jones-Engel,L.; McKenzie,P.; Krauss,S.; Webby,R.J.; Feeroz,M.M.; Webster,R.G.
EPI_ISL_306668	Bangladesh	2017-Feb-15	NA	H9	Import from public-domain	Barman,S.; Turner,J.C.; Hasan,M.K.; Akhtar,S.; Franks,J.; El-Shesheny,R.; Walker,D.; Seiler,P.; Friedman,K.; Kercher,L.; Kayali,G.; Jones-Engel,L.; McKenzie,P.; Krauss,S.; Webby,R.J.; Feeroz,M.M.; Webster,R.G.
EPI_ISL_306654	Bangladesh	2017-Mar-28	NA	H9	Import from public-domain	Barman,S.; Turner,J.C.; Hasan,M.K.; Akhtar,S.; Franks,J.; El-Shesheny,R.; Walker,D.; Seiler,P.; Friedman,K.; Kercher,L.; Kayali,G.; Jones-Engel,L.; McKenzie,P.; Krauss,S.; Webby,R.J.; Feeroz,M.M.; Webster,R.G.
EPI_ISL_306665	Bangladesh	2017-Mar-29	NA	H9	Import from public-domain	Barman,S.; Turner,J.C.; Hasan,M.K.; Akhtar,S.; Franks,J.; El-Shesheny,R.; Walker,D.; Seiler,P.; Friedman,K.; Kercher,L.; Kayali,G.; Jones-Engel,L.; McKenzie,P.; Krauss,S.; Webby,R.J.; Feeroz,M.M.; Webster,R.G.
EPI_ISL_306670	Bangladesh	2017-Apr-19	NA	H9	Import from public-domain	Barman,S.; Turner,J.C.; Hasan,M.K.; Akhtar,S.; Franks,J.; El-Shesheny,R.; Walker,D.; Seiler,P.; Friedman,K.; Kercher,L.; Kayali,G.; Jones-Engel,L.; McKenzie,P.; Krauss,S.; Webby,R.J.; Feeroz,M.M.; Webster,R.G.
EPI_ISL_329594	Bangladesh	2017-May-20	NA	H9	Import from public-domain	Barman,S.; Turner,J.C.; Hasan,M.K.; Akhtar,S.; Franks,J.; El-Shesheny,R.; Walker,D.; Seiler,P.; Friedman,K.; Kercher,L.; Kayali,G.; Jones-Engel,L.; McKenzie,P.; Krauss,S.; Webby,R.J.; Feeroz,M.; Webster,R.G.

EPI_ISL_329596	Bangladesh	2017-Jun-11	NA	H9	Import from public-domain	Barman,S.; Turner,J.C.; Hasan,M.K.; Akhtar,S.; Franks,J.; El-Shesheny,R.; Walker,D.; Seiler,P.; Friedman,K.; Kercher,L.; Kayali,G.; Jones-Engel,L.; McKenzie,P.; Krauss,S.; Webby,R.J.; Feeroz,M.; Webster,R.G.
EPI_ISL_329593	Bangladesh	2017-Jun-11	NA	H9	Import from public-domain	Barman,S.; Turner,J.C.; Hasan,M.K.; Akhtar,S.; Franks,J.; El-Shesheny,R.; Walker,D.; Seiler,P.; Friedman,K.; Kercher,L.; Kayali,G.; Jones-Engel,L.; McKenzie,P.; Krauss,S.; Webby,R.J.; Feeroz,M.; Webster,R.G.
EPI_ISL_397430	Bangladesh	2017-Jun-11	NA	H9	Import from public-domain	Barman,S.; Turner,J.C.; Hasan,M.K.; Akhtar,S.; Franks,J.; El-Shesheny,R.; Walker,D.; Seiler,P.; Friedman,K.; Kercher,L.; Kayali,G.; Jones-Engel,L.; McKenzie,P.; Krauss,S.; Webby,R.J.; Feeroz,M.; Webster,R.G.
EPI_ISL_329578	Bangladesh	2017-Jul-21	NA	H9	Import from public-domain	Barman,S.; Turner,J.C.; Hasan,M.K.; Akhtar,S.; Franks,J.; El-Shesheny,R.; Walker,D.; Seiler,P.; Friedman,K.; Kercher,L.; Kayali,G.; Jones-Engel,L.; McKenzie,P.; Krauss,S.; Webby,R.J.; Feeroz,M.; Webster,R.G.
EPI_ISL_329577	Bangladesh	2017-Jul-21	NA	H9	Import from public-domain	Barman,S.; Turner,J.C.; Hasan,M.K.; Akhtar,S.; Franks,J.; El-Shesheny,R.; Walker,D.; Seiler,P.; Friedman,K.; Kercher,L.; Kayali,G.; Jones-Engel,L.; McKenzie,P.; Krauss,S.; Webby,R.J.; Feeroz,M.; Webster,R.G.
EPI_ISL_329574	Bangladesh	2017-Jul-21	NA	H9	Import from public-domain	Barman,S.; Turner,J.C.; Hasan,M.K.; Akhtar,S.; Franks,J.; El-Shesheny,R.; Walker,D.; Seiler,P.; Friedman,K.; Kercher,L.; Kayali,G.; Jones-Engel,L.; McKenzie,P.; Krauss,S.; Webby,R.J.; Feeroz,M.; Webster,R.G.
EPI_ISL_329567	Bangladesh	2017-Jul-21	NA	H9	Import from public-domain	Barman,S.; Turner,J.C.; Hasan,M.K.; Akhtar,S.; Franks,J.; El-Shesheny,R.; Walker,D.; Seiler,P.; Friedman,K.; Kercher,L.; Kayali,G.; Jones-Engel,L.; McKenzie,P.; Krauss,S.; Webby,R.J.; Feeroz,M.; Webster,R.G.
EPI_ISL_329589	Bangladesh	2017-Jul-21	NA	H9	Import from public-domain	Barman,S.; Turner,J.C.; Hasan,M.K.; Akhtar,S.; Franks,J.; El-Shesheny,R.; Walker,D.; Seiler,P.; Friedman,K.; Kercher,L.; Kayali,G.; Jones-Engel,L.; McKenzie,P.; Krauss,S.; Webby,R.J.; Feeroz,M.; Webster,R.G.
EPI_ISL_329585	Bangladesh	2017-Jul-21	NA	H9	Import from public-domain	Barman,S.; Turner,J.C.; Hasan,M.K.; Akhtar,S.; Franks,J.; El-Shesheny,R.; Walker,D.; Seiler,P.; Friedman,K.; Kercher,L.; Kayali,G.; Jones-Engel,L.; McKenzie,P.; Krauss,S.; Webby,R.J.; Feeroz,M.; Webster,R.G.
EPI_ISL_329583	Bangladesh	2017-Jul-21	NA	H9	Import from public-domain	Barman,S.; Turner,J.C.; Hasan,M.K.; Akhtar,S.; Franks,J.; El-Shesheny,R.; Walker,D.; Seiler,P.; Friedman,K.; Kercher,L.; Kayali,G.; Jones-Engel,L.; McKenzie,P.; Krauss,S.; Webby,R.J.; Feeroz,M.; Webster,R.G.
EPI_ISL_329579	Bangladesh	2017-Jul-21	NA	H9	Import from public-domain	Barman,S.; Turner,J.C.; Hasan,M.K.; Akhtar,S.; Franks,J.; El-Shesheny,R.; Walker,D.; Seiler,P.; Friedman,K.; Kercher,L.; Kayali,G.; Jones-Engel,L.; McKenzie,P.; Krauss,S.; Webby,R.J.; Feeroz,M.; Webster,R.G.
EPI_ISL_397434	Bangladesh	2017-Jul-21	NA	H9	Import from public-domain	Barman,S.; Turner,J.C.; Hasan,M.K.; Akhtar,S.; Franks,J.; El-Shesheny,R.; Walker,D.; Seiler,P.; Friedman,K.; Kercher,L.; Kayali,G.; Jones-Engel,L.; McKenzie,P.; Krauss,S.; Webby,R.J.; Feeroz,M.; Webster,R.G.
EPI_ISL_329576	Bangladesh	2017-Aug-17	NA	H9	Import from public-domain	Barman,S.; Turner,J.C.; Hasan,M.K.; Akhtar,S.; Franks,J.; El-Shesheny,R.; Walker,D.; Seiler,P.; Friedman,K.; Kercher,L.; Kayali,G.; Jones-Engel,L.; McKenzie,P.; Krauss,S.; Webby,R.J.; Feeroz,M.; Webster,R.G.
EPI_ISL_329572	Bangladesh	2017-Aug-17	NA	H9	Import from public-domain	Barman,S.; Turner,J.C.; Hasan,M.K.; Akhtar,S.; Franks,J.; El-Shesheny,R.; Walker,D.; Seiler,P.; Friedman,K.; Kercher,L.; Kayali,G.; Jones-Engel,L.; McKenzie,P.; Krauss,S.; Webby,R.J.; Feeroz,M.; Webster,R.G.
EPI_ISL_329580	Bangladesh	2017-Aug-17	NA	H9	Import from public-domain	Barman,S.; Turner,J.C.; Hasan,M.K.; Akhtar,S.; Franks,J.; El-Shesheny,R.; Walker,D.; Seiler,P.; Friedman,K.; Kercher,L.; Kayali,G.; Jones-Engel,L.; McKenzie,P.; Krauss,S.; Webby,R.J.; Feeroz,M.; Webster,R.G.
EPI_ISL_397432	Bangladesh	2017-Aug-17	NA	H9	Import from public-domain	Barman,S.; Turner,J.C.; Hasan,M.K.; Akhtar,S.; Franks,J.; El-Shesheny,R.; Walker,D.; Seiler,P.; Friedman,K.; Kercher,L.; Kayali,G.; Jones-Engel,L.; McKenzie,P.; Krauss,S.; Webby,R.J.; Feeroz,M.; Webster,R.G.
EPI_ISL_329569	Bangladesh	2017-Sep-28	NA	H9	Import from public-domain	Barman,S.; Turner,J.C.; Hasan,M.K.; Akhtar,S.; Franks,J.; El-Shesheny,R.; Walker,D.; Seiler,P.; Friedman,K.; Kercher,L.; Kayali,G.; Jones-Engel,L.; McKenzie,P.; Krauss,S.; Webby,R.J.; Feeroz,M.; Webster,R.G.
EPI_ISL_329561	Bangladesh	2017-Sep-28	NA	H9	Import from public-domain	Barman,S.; Turner,J.C.; Hasan,M.K.; Akhtar,S.; Franks,J.; El-Shesheny,R.; Walker,D.; Seiler,P.; Friedman,K.; Kercher,L.; Kayali,G.; Jones-Engel,L.; McKenzie,P.; Krauss,S.; Webby,R.J.; Feeroz,M.; Webster,R.G.
EPI_ISL_329559	Bangladesh	2017-Sep-28	NA	H9	Import from public-domain	Barman,S.; Turner,J.C.; Hasan,M.K.; Akhtar,S.; Franks,J.; El-Shesheny,R.; Walker,D.; Seiler,P.; Friedman,K.; Kercher,L.; Kayali,G.; Jones-Engel,L.; McKenzie,P.; Krauss,S.; Webby,R.J.; Feeroz,M.; Webster,R.G.
EPI_ISL_379724	Bangladesh	2017-Nov-28	NA	H9	Import from public-domain	Barman,S.; Turner,J.C.; Hasan,M.K.; Akhtar,S.; Franks,J.; El-Shesheny,R.; Walker,D.; Seiler,P.; Friedman,K.; Kercher,L.; Kayali,G.; Jones-Engel,L.; McKenzie,P.; Krauss,S.; Webby,R.J.; Feeroz,M.M.; Webster,R.G.
EPI_ISL_379706	Bangladesh	2017-Nov-28	NA	H9	Import from public-domain	Barman,S.; Turner,J.C.; Hasan,M.K.; Akhtar,S.; Franks,J.; El-Shesheny,R.; Walker,D.; Seiler,P.; Friedman,K.; Kercher,L.; Kayali,G.; Jones-Engel,L.; McKenzie,P.; Krauss,S.; Webby,R.J.; Feeroz,M.M.; Webster,R.G.
EPI_ISL_379705	Bangladesh	2017-Nov-28	NA	H9	Import from public-domain	Barman,S.; Turner,J.C.; Hasan,M.K.; Akhtar,S.; Franks,J.; El-Shesheny,R.; Walker,D.; Seiler,P.; Friedman,K.; Kercher,L.; Kayali,G.; Jones-Engel,L.; McKenzie,P.; Krauss,S.; Webby,R.J.; Feeroz,M.M.; Webster,R.G.
EPI_ISL_379694	Bangladesh	2017-Nov-28	NA	H9	Import from public-domain	Barman,S.; Turner,J.C.; Hasan,M.K.; Akhtar,S.; Franks,J.; El-Shesheny,R.; Walker,D.; Seiler,P.; Friedman,K.; Kercher,L.; Kayali,G.; Jones-Engel,L.; McKenzie,P.; Krauss,S.; Webby,R.J.; Feeroz,M.M.; Webster,R.G.

EPI_ISL_379688	Bangladesh	2017-Nov-28	NA	H9	Import from public-domain	Barman,S.; Turner,J.C.; Hasan,M.K.; Akhtar,S.; Franks,J.; El-Shesheny,R.; Walker,D.; Seiler,P.; Friedman,K.; Kercher,L.; Kayali,G.; Jones-Engel,L.; McKenzie,P.; Krauss,S.; Webby,R.J.; Feeroz,M.M.; Webster,R.G.
EPI_ISL_379971	Bangladesh	2017-Nov-28	NA	H9	Import from public-domain	Barman,S.; Turner,J.C.; Hasan,M.K.; Akhtar,S.; Franks,J.; El-Shesheny,R.; Walker,D.; Seiler,P.; Friedman,K.; Kercher,L.; Kayali,G.; Jones-Engel,L.; McKenzie,P.; Krauss,S.; Webby,R.J.; Feeroz,M.M.; Webster,R.G.
EPI_ISL_379701	Bangladesh	2017-Dec-25	NA	H9	Import from public-domain	Barman,S.; Turner,J.C.; Hasan,M.K.; Akhtar,S.; Franks,J.; El-Shesheny,R.; Walker,D.; Seiler,P.; Friedman,K.; Kercher,L.; Kayali,G.; Jones-Engel,L.; McKenzie,P.; Krauss,S.; Webby,R.J.; Feeroz,M.M.; Webster,R.G.
EPI_ISL_379698	Bangladesh	2017-Dec-25	NA	H9	Import from public-domain	Barman,S.; Turner,J.C.; Hasan,M.K.; Akhtar,S.; Franks,J.; El-Shesheny,R.; Walker,D.; Seiler,P.; Friedman,K.; Kercher,L.; Kayali,G.; Jones-Engel,L.; McKenzie,P.; Krauss,S.; Webby,R.J.; Feeroz,M.M.; Webster,R.G.
EPI_ISL_379689	Bangladesh	2017-Dec-25	NA	H9	Import from public-domain	Barman,S.; Turner,J.C.; Hasan,M.K.; Akhtar,S.; Franks,J.; El-Shesheny,R.; Walker,D.; Seiler,P.; Friedman,K.; Kercher,L.; Kayali,G.; Jones-Engel,L.; McKenzie,P.; Krauss,S.; Webby,R.J.; Feeroz,M.M.; Webster,R.G.
EPI_ISL_379687	Bangladesh	2017-Dec-25	NA	H9	Import from public-domain	Barman,S.; Turner,J.C.; Hasan,M.K.; Akhtar,S.; Franks,J.; El-Shesheny,R.; Walker,D.; Seiler,P.; Friedman,K.; Kercher,L.; Kayali,G.; Jones-Engel,L.; McKenzie,P.; Krauss,S.; Webby,R.J.; Feeroz,M.M.; Webster,R.G.
EPI_ISL_379728	Bangladesh	2017-Dec-25	NA	H9	Import from public-domain	Barman,S.; Turner,J.C.; Hasan,M.K.; Akhtar,S.; Franks,J.; El-Shesheny,R.; Walker,D.; Seiler,P.; Friedman,K.; Kercher,L.; Kayali,G.; Jones-Engel,L.; McKenzie,P.; Krauss,S.; Webby,R.J.; Feeroz,M.M.; Webster,R.G.
EPI_ISL_379973	Bangladesh	2017-Dec-25	NA	H9	Import from public-domain	Barman,S.; Turner,J.C.; Hasan,M.K.; Akhtar,S.; Franks,J.; El-Shesheny,R.; Walker,D.; Seiler,P.; Friedman,K.; Kercher,L.; Kayali,G.; Jones-Engel,L.; McKenzie,P.; Krauss,S.; Webby,R.J.; Feeroz,M.M.; Webster,R.G.
EPI_ISL_379731	Bangladesh	2018-Jan-23	NA	H9	Import from public-domain	Barman,S.; Turner,J.C.; Hasan,M.K.; Akhtar,S.; Franks,J.; El-Shesheny,R.; Walker,D.; Seiler,P.; Friedman,K.; Kercher,L.; Kayali,G.; Jones-Engel,L.; McKenzie,P.; Krauss,S.; Webby,R.J.; Feeroz,M.M.; Webster,R.G.
EPI_ISL_379713	Bangladesh	2018-Jan-23	NA	H9	Import from public-domain	Barman,S.; Turner,J.C.; Hasan,M.K.; Akhtar,S.; Franks,J.; El-Shesheny,R.; Walker,D.; Seiler,P.; Friedman,K.; Kercher,L.; Kayali,G.; Jones-Engel,L.; McKenzie,P.; Krauss,S.; Webby,R.J.; Feeroz,M.M.; Webster,R.G.
EPI_ISL_379707	Bangladesh	2018-Jan-23	NA	H9	Import from public-domain	Barman,S.; Turner,J.C.; Hasan,M.K.; Akhtar,S.; Franks,J.; El-Shesheny,R.; Walker,D.; Seiler,P.; Friedman,K.; Kercher,L.; Kayali,G.; Jones-Engel,L.; McKenzie,P.; Krauss,S.; Webby,R.J.; Feeroz,M.M.; Webster,R.G.
EPI_ISL_379690	Bangladesh	2018-Jan-23	NA	H9	Import from public-domain	Barman,S.; Turner,J.C.; Hasan,M.K.; Akhtar,S.; Franks,J.; El-Shesheny,R.; Walker,D.; Seiler,P.; Friedman,K.; Kercher,L.; Kayali,G.; Jones-Engel,L.; McKenzie,P.; Krauss,S.; Webby,R.J.; Feeroz,M.M.; Webster,R.G.
EPI_ISL_379972	Bangladesh	2018-Jan-23	NA	H9	Import from public-domain	Barman,S.; Turner,J.C.; Hasan,M.K.; Akhtar,S.; Franks,J.; El-Shesheny,R.; Walker,D.; Seiler,P.; Friedman,K.; Kercher,L.; Kayali,G.; Jones-Engel,L.; McKenzie,P.; Krauss,S.; Webby,R.J.; Feeroz,M.M.; Webster,R.G.
EPI_ISL_388024	Bangladesh	2018-Apr-26	NA	H9	Import from public-domain	Barman,S.; Turner,J.C.; Hasan,M.; Akhtar,S.; Franks,J.; El-Shesheny,R.; Walker,D.; Seiler,P.; Friedman,K.; Kercher,L.; McKenzie,P.; Webby,R.J.; Feeroz,M.; Webster,R.G.
EPI_ISL_388023	Bangladesh	2018-Apr-26	NA	H9	Import from public-domain	Barman,S.; Turner,J.C.; Hasan,M.; Akhtar,S.; Franks,J.; El-Shesheny,R.; Walker,D.; Seiler,P.; Friedman,K.; Kercher,L.; McKenzie,P.; Webby,R.J.; Feeroz,M.; Webster,R.G.
EPI_ISL_388026	Bangladesh	2018-Apr-28	NA	H9	Import from public-domain	Barman,S.; Turner,J.C.; Hasan,M.; Akhtar,S.; Franks,J.; El-Shesheny,R.; Walker,D.; Seiler,P.; Friedman,K.; Kercher,L.; McKenzie,P.; Webby,R.J.; Feeroz,M.; Webster,R.G.
EPI_ISL_388032	Bangladesh	2018-Jun-05	NA	H9	Import from public-domain	Barman,S.; Turner,J.C.; Hasan,M.; Akhtar,S.; Franks,J.; El-Shesheny,R.; Walker,D.; Seiler,P.; Friedman,K.; Kercher,L.; McKenzie,P.; Webby,R.J.; Feeroz,M.; Webster,R.G.
EPI_ISL_388031	Bangladesh	2018-Jun-05	NA	H9	Import from public-domain	Barman,S.; Turner,J.C.; Hasan,M.; Akhtar,S.; Franks,J.; El-Shesheny,R.; Walker,D.; Seiler,P.; Friedman,K.; Kercher,L.; McKenzie,P.; Webby,R.J.; Feeroz,M.; Webster,R.G.
EPI_ISL_388022	Bangladesh	2018-Jun-05	NA	H9	Import from public-domain	Barman,S.; Turner,J.C.; Hasan,M.; Akhtar,S.; Franks,J.; El-Shesheny,R.; Walker,D.; Seiler,P.; Friedman,K.; Kercher,L.; McKenzie,P.; Webby,R.J.; Feeroz,M.; Webster,R.G.
EPI_ISL_388012	Bangladesh	2018-Jun-05	NA	H9	Import from public-domain	Barman,S.; Turner,J.C.; Hasan,M.; Akhtar,S.; Franks,J.; El-Shesheny,R.; Walker,D.; Seiler,P.; Friedman,K.; Kercher,L.; McKenzie,P.; Webby,R.J.; Feeroz,M.; Webster,R.G.
EPI_ISL_387991	Bangladesh	2018-Aug-09	NA	H9	Import from public-domain	Barman,S.; Turner,J.C.; Hasan,M.; Akhtar,S.; Franks,J.; El-Shesheny,R.; Walker,D.; Seiler,P.; Friedman,K.; Kercher,L.; McKenzie,P.; Webby,R.J.; Feeroz,M.; Webster,R.G.
EPI_ISL_388010	Bangladesh	2018-Oct-30	NA	H9	Import from public-domain	Barman,S.; Turner,J.C.; Hasan,M.; Akhtar,S.; Franks,J.; El-Shesheny,R.; Walker,D.; Seiler,P.; Friedman,K.; Kercher,L.; McKenzie,P.; Webby,R.J.; Feeroz,M.; Webster,R.G.
EPI_ISL_387995	Bangladesh	2018-Dec-15	NA	H9	Import from public-domain	Barman,S.; Turner,J.C.; Hasan,M.; Akhtar,S.; Franks,J.; El-Shesheny,R.; Walker,D.; Seiler,P.; Friedman,K.; Kercher,L.; McKenzie,P.; Webby,R.J.; Feeroz,M.; Webster,R.G.

EPI_ISL_387985	Bangladesh	2018-Dec-18	NA	H9	Import from public-domain	Barman,S.; Turner,J.C.; Hasan,M.; Akhtar,S.; Franks,J.; El-Shesheny,R.; Walker,D.; Seiler,P.; Friedman,K.; Kercher,L.; McKenzie,P.; Webby,R.J.; Feeroz,M.; Webster,R.G.
EPI_ISL_387978	Bangladesh	2018-Dec-18	NA	H9	Import from public-domain	Barman,S.; Turner,J.C.; Hasan,M.; Akhtar,S.; Franks,J.; El-Shesheny,R.; Walker,D.; Seiler,P.; Friedman,K.; Kercher,L.; McKenzie,P.; Webby,R.J.; Feeroz,M.; Webster,R.G.
EPI_ISL_387979	Bangladesh	2018-Dec-18	NA	H9	Import from public-domain	Barman,S.; Turner,J.C.; Hasan,M.; Akhtar,S.; Franks,J.; El-Shesheny,R.; Walker,D.; Seiler,P.; Friedman,K.; Kercher,L.; McKenzie,P.; Webby,R.J.; Feeroz,M.; Webster,R.G.
EPI_ISL_387983	Bangladesh	2018-Dec-18	NA	H9	Import from public-domain	Barman,S.; Turner,J.C.; Hasan,M.; Akhtar,S.; Franks,J.; El-Shesheny,R.; Walker,D.; Seiler,P.; Friedman,K.; Kercher,L.; McKenzie,P.; Webby,R.J.; Feeroz,M.; Webster,R.G.
EPI_ISL_503506	Bangladesh	2019-Feb-19	NA	H9	Import from public-domain	Barman,S.; Turner,J.C.; Hasan,M.; Akhtar,S.; Franks,J.; El-Shesheny,R.; Walker,D.; Seiler,P.; Mukherjee,N.; Kercher,L.; McKenzie,P.; Feeroz,M.; Webby,R.J.
EPI_ISL_503494	Bangladesh	2019-Feb-19	NA	H9	Import from public-domain	Barman,S.; Turner,J.C.; Hasan,M.; Akhtar,S.; Franks,J.; El-Shesheny,R.; Walker,D.; Seiler,P.; Mukherjee,N.; Kercher,L.; McKenzie,P.; Feeroz,M.; Webby,R.J.
EPI_ISL_503518	Bangladesh	2019-Jul-10	NA	H9	Import from public-domain	Barman,S.; Turner,J.C.; Hasan,M.; Akhtar,S.; Franks,J.; El-Shesheny,R.; Walker,D.; Seiler,P.; Mukherjee,N.; Kercher,L.; McKenzie,P.; Feeroz,M.; Webby,R.J.
EPI_ISL_503512	Bangladesh	2019-Jul-11	NA	H9	Import from public-domain	Barman,S.; Turner,J.C.; Hasan,M.; Akhtar,S.; Franks,J.; El-Shesheny,R.; Walker,D.; Seiler,P.; Mukherjee,N.; Kercher,L.; McKenzie,P.; Feeroz,M.; Webby,R.J.
EPI_ISL_503517	Bangladesh	2019-Aug-19	NA	H9	Import from public-domain	Barman,S.; Turner,J.C.; Hasan,M.; Akhtar,S.; Franks,J.; El-Shesheny,R.; Walker,D.; Seiler,P.; Mukherjee,N.; Kercher,L.; McKenzie,P.; Feeroz,M.; Webby,R.J.
EPI_ISL_503493	Bangladesh	2019-Aug-19	NA	H9	Import from public-domain	Barman,S.; Turner,J.C.; Hasan,M.; Akhtar,S.; Franks,J.; El-Shesheny,R.; Walker,D.; Seiler,P.; Mukherjee,N.; Kercher,L.; McKenzie,P.; Feeroz,M.; Webby,R.J.
EPI_ISL_503536	Bangladesh	2019-Sep-13	NA	H9	Import from public-domain	Barman,S.; Turner,J.C.; Hasan,M.; Akhtar,S.; Franks,J.; El-Shesheny,R.; Walker,D.; Seiler,P.; Mukherjee,N.; Kercher,L.; McKenzie,P.; Feeroz,M.; Webby,R.J.
EPI_ISL_503529	Bangladesh	2019-Sep-13	NA	H9	Import from public-domain	Barman,S.; Turner,J.C.; Hasan,M.; Akhtar,S.; Franks,J.; El-Shesheny,R.; Walker,D.; Seiler,P.; Mukherjee,N.; Kercher,L.; McKenzie,P.; Feeroz,M.; Webby,R.J.
EPI_ISL_503515	Bangladesh	2019-Sep-13	NA	H9	Import from public-domain	Barman,S.; Turner,J.C.; Hasan,M.; Akhtar,S.; Franks,J.; El-Shesheny,R.; Walker,D.; Seiler,P.; Mukherjee,N.; Kercher,L.; McKenzie,P.; Feeroz,M.; Webby,R.J.
EPI_ISL_503486	Bangladesh	2019-Sep-13	NA	H9	Import from public-domain	Barman,S.; Turner,J.C.; Hasan,M.; Akhtar,S.; Franks,J.; El-Shesheny,R.; Walker,D.; Seiler,P.; Mukherjee,N.; Kercher,L.; McKenzie,P.; Feeroz,M.; Webby,R.J.
EPI_ISL_503496	Bangladesh	2019-Sep-14	NA	H9	Import from public-domain	Barman,S.; Turner,J.C.; Hasan,M.; Akhtar,S.; Franks,J.; El-Shesheny,R.; Walker,D.; Seiler,P.; Mukherjee,N.; Kercher,L.; McKenzie,P.; Feeroz,M.; Webby,R.J.
EPI_ISL_503504	Bangladesh	2019-Oct-16	NA	H9	Import from public-domain	Barman,S.; Turner,J.C.; Hasan,M.; Akhtar,S.; Franks,J.; El-Shesheny,R.; Walker,D.; Seiler,P.; Mukherjee,N.; Kercher,L.; McKenzie,P.; Feeroz,M.; Webby,R.J.
